# Punic people were genetically diverse with almost no Levantine ancestors

**DOI:** 10.1038/s41586-025-08913-3

**Published:** 2025-04-23

**Authors:** Harald Ringbauer, Ayelet Salman-Minkov, Dalit Regev, Iñigo Olalde, Tomer Peled, Luca Sineo, Gioacchino Falsone, Peter van Dommelen, Alissa Mittnik, Iosif Lazaridis, Davide Pettener, Maria Bofill, Ana Mezquida, Benjamí Costa, Helena Jiménez, Patricia Smith, Stefania Vai, Alessandra Modi, Arie Shaus, Kim Callan, Elizabeth Curtis, Aisling Kearns, Ann Marie Lawson, Matthew Mah, Adam Micco, Jonas Oppenheimer, Liju Qiu, Kristin Stewardson, J. Noah Workman, Nicholas Márquez-Grant, Antonio M. Sáez Romero, María Luisa Lavado Florido, Juan Manuel Jimenez-Arenas, Isidro Jorge Toro Moyano, Enrique Viguera, Jose Suarez Padilla, Sonia López Chamizo, Tomas Marques-Bonet, Esther Lizano, Alicia Rodero Riaza, Francesca Olivieri, Pamela Toti, Valentina Giuliana, Alon Barash, Liran Carmel, Elisabetta Boaretto, Marina Faerman, Michaela Lucci, Francesco La Pastina, Alessia Nava, Francesco Genchi, Carla Del Vais, Gabriele Lauria, Francesca Meli, Paola Sconzo, Giulio Catalano, Elisabetta Cilli, Anna Chiara Fariselli, Francesco Fontani, Donata Luiselli, Brendan J. Culleton, Swapan Mallick, Nadin Rohland, Lorenzo Nigro, Alfredo Coppa, David Caramelli, Ron Pinhasi, Carles Lalueza-Fox, Ilan Gronau, David Reich

**Affiliations:** 1 Department of Human Evolutionary Biology, Harvard University, Cambridge, MA, USA; 2 Max Planck Harvard Research Center for the Archaeoscience of the Ancient Mediterranean (MHAAM), Leipzig, Germany; 3 Department of Archaeogenetics, Max Planck Institute for Evolutionary Anthropology, Leipzig, Germany; 4 Efi Arazi School of Computer Science, Reichman University, Herzliya, Israel; 5 Israel Antiquities Authority, Jerusalem, Israel; 6 BIOMICs Research Group, Department of Zoology and Animal Cell Biology, University of the Basque Country UPV/EHU, Vitoria-Gasteiz, Spain; 7 Dept. STEBICEF, Laboratory of Anthropology, University of Palermo, Palermo, Italy; 8 Dept. Culture e Società, University of Palermo, Palermo, Italy; 9 Brown University, Providence, RI, USA; 10 University of Bologna, Bologna, Italy; 11 Archaeological Museum of Ibiza and Formentera, Eivissa, Spain; 12 Faculties of Medicine and Dental Medicine, The Hebrew University of Jerusalem, Jerusalem, Israel; 13 University of Florence, Florence, Italy; 14 Department of Genetics, Harvard Medical School, Boston, MA, USA; 15 Broad Institute of MIT and Harvard, Cambridge, MA, USA; 16 Howard Hughes Medical Institute, Harvard Medical School, Boston, MA, USA; 17 Cranfield Forensic Institute, Cranfield University, UK; 18 Universidad de Sevilla, Seville, Spain; 19 Universidad de Granada, Granada, Spain; 20 Museo Arqueológico de Granada, Granada, Spain; 21 Universidad de Málaga, Málaga, Spain; 22 Unidad de Paleobiología, ICP-CERCA, Unidad Asociada al CSIC por el IBE UPF-CSIC, Cerdanyola del Vallès, Barcelona, Spain.; 23 Museo Arqueológico Nacional Madrid, Madrid, Spain; 24 Ministry of cultural heritage, Sicily region, Palermo, Italy; 25 The Giuseppe Whitaker Foundation, Motya, Italy; 26 University of Palermo, Department of Archaeology, Palermo, Italy; 27 Bar Ilan University, The Azrieli Faculty of Medicine, Safed, Israel; 28 Department of Genetics, The Alexander Silberman Institute of Life Sciences, The Hebrew University of Jerusalem, Jerusalem, Israel; 29 Weizmann Institute of Science, Scientific Archaeology Unit, D-REAMS Radiocarbon Dating Laboratory, Rehovot, Israel; 30 The National Natural History Collections, The Hebrew University of Jerusalem, Jerusalem, Israel; 31 Department of Environmental Biology, Sapienza University of Rome, Rome, Italy; 32 Department of Odontostomatological and Maxillofacial Sciences, Sapienza University of Rome, Rome, Italy; 33 Italian Institute of Oriental Studies, Sapienza University of Rome, Rome, Italy; 34 Università degli Studi di Cagliari, Cagliari, Italy; 35 Dept. STEBICEF, Laboratory of Anthropology, University of Palermo, Palermo, Italy; 36 Dept. Culture e Società, University of Palermo, Palermo, Italy; 37 Dept. STEBICEF, Laboratory of Anthropology, University of Palermo, Palermo, Italy; 38 Department of Cultural Heritage, University of Bologna, Ravenna, Italy; 39 Institute of Energy and the Environment, Penn State University, Pennsylvania, USA; 40 Department of Environmental Biology, Sapienza University of Rome, Rome, Italy; 41 Department of Ancient World Studies, Sapienza University of Rome, Italy; 42 Department of Evolutionary Anthropology, University of Vienna, Vienna, Austria; 43 Natural Sciences Museum of Barcelona, Barcelona, Spain; 44 Institute of Evolutionary Biology (UPF-CSIC), PRBB, Barcelona, Spain; 45 Ikerbasque—Basque Foundation of Science, Bilbao, Spain; 46 Department of Evolution and Ecology, University of California, Davis, CA, USA; 47 Department of Archaeology and Ancient Near Eastern Civilizations, Tel Aviv University, Tel Aviv, Israel; 48 Department of Data Science, Mount Holyoke College, South Hadley, MA, USA

## Abstract

The maritime Phoenician civilization from the Levant transformed the entire Mediterranean during the first millennium BCE^1–3^. However, the extent of human movement between the Levantine Phoenician homeland and Phoenician-Punic settlements in the central and western Mediterranean has been unclear in the absence of comprehensive ancient DNA studies. We generated genome-wide data for 210 individuals, including 196 from 14 sites traditionally identified as Phoenician and Punic in the Levant, North Africa, Iberia, Sicily, Sardinia, and Ibiza, and an early Iron Age individual from Algeria. Levantine Phoenicians made little genetic contribution to Punic settlements in the central and western Mediterranean between the 6th and 2nd centuries BCE, despite abundant archaeological evidence of cultural, historical, linguistic, and religious links^4^. Instead, these inheritors of Levantine Phoenician culture derived most of their ancestry from a genetic profile similar to that of Sicily and the Aegean. Much of the remaining ancestry originated from North Africa, reflecting the growing influence of Carthage^5^; however, this was a minority contributor of ancestry in all sampled sites, including in Carthage itself. Different Punic sites across the central and western Mediterranean show similar patterns of high genetic diversity. We also detect genetic relationships across the Mediterranean, reflecting shared demographic processes shaped the Punic world.

Canaanite-Phoenician culture emerged in the city-states of the Bronze Age Levant. In the early first millennium BCE, Phoenicians established a vast network of trading posts as far as southwest Iberia^1–4^, spreading their culture, religion, and language to the central-western Mediterranean^6^. By the mid-6th century BCE, Carthage, a Phoenician coastal colony in present-day Tunisia, emerged as the dominant power in the central-western Mediterranean while the influence of Levantine cities declined as they fell under neo-Assyrian and neo-Babylonian control. Carthage came into large-scale violent conflicts with Greeks in the 5th-4th centuries BCE and with Rome in the 3rd-2nd centuries BCE, culminating in its destruction in 146 BCE and annexation by the Roman Republic^7,8^.

Many historians and archaeologists use the Roman term “Punic” to refer to culturally Phoenician communities in the West Mediterranean that were associated with or even ruled by Carthage after the mid-6th century BCE^5,9–11^. While problems of such a simplifying label have been highlighted^12^, we follow the recent *Handbook of the Phoenician and Punic Mediterranean*^6^ and use “Punic” to refer to sites in the central and western Mediterranean associated with material culture with Phoenician antecedents or connotations between the late 6th and 2nd centuries BCE, a time frame of Carthaginian hegemony in this region. We also use the term to refer to the people buried at these sites. However, we caution that by labeling sites and people as “Punic” we do not make any claim of a homogeneous culture or ethnicity. Moreover, our distinction between Phoenician and Punic sites does not question the Levantine cultural origins of their inhabitants, nor does it address the question of whether the founders of Phoenician-Punic sites such as Carthage and Cádiz were Levantine^13^ we do not have the early samples from these sites needed to address these issues. We use “Punic” in this paper simply as a means to refer to a group of sites and their inhabitants as defined above, for which our analysis finds shared genetic signatures.

Ancient DNA (aDNA) reveals information regarding genetic ancestry, which can help resolve questions beyond the reach of conventional historical and archaeological data. Previous aDNA studies of Punic remains focused on mitochondrial DNA (mtDNA), revealing diverse maternal lineages^14–16^. However, as mtDNA only traces a single lineage, those analyses provide limited statistical resolution compared to genome-wide data. Whole-genome analysis of five Bronze Age individuals from Sidon^17^ and twelve Iron Age individuals from Beirut^18^ indicates that Phoenicians in the Levant were genetically similar to local Bronze Age Canaanite people. However, a mitochondrial genome from Carthage^14^ and whole-genome data from twelve individuals from the nearby rural Punic site of Kerkouane^19^ show substantial Southern European ancestry as well as indigenous North African ancestry. Partial North African ancestry was also found in genome-wide data from eight individuals from two Punic sites in Sardinia, combined with a broad eastern Mediterranean ancestry^20^. Together with analysis of the whole genome sequence of an individual from Ibiza^21^, which was also interpreted to harbor eastern Mediterranean ancestry, this suggested that Punic people had complex ancestry. However, these observations were based on analyzing genome-wide data from a combined total of 21 individuals and did not include the key regions of Iberia or Sicily or settlements with early Phoenician foundations, such as Carthage, Motya, or Cádiz. Due to the small sample size and complex combination of ancestries, it has not been possible to study ancestry across Punic sites systematically.

## Data set assembly

**Figure 1:**
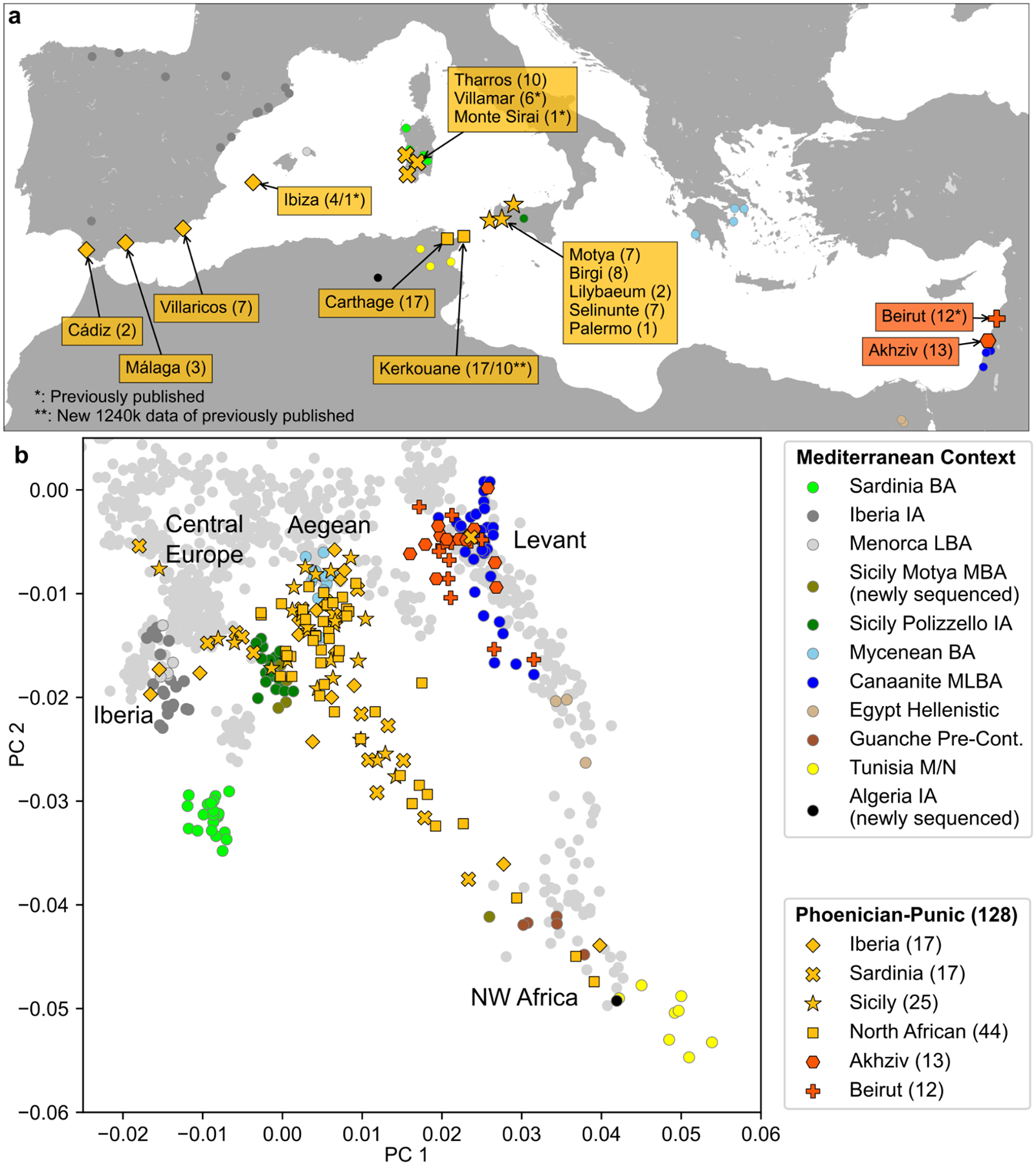
Sample Overview and Principal Component Analysis. **(a)** Locations of archaeological sites for which we analyzed aDNA. We indicate the number of individuals with newly generated aDNA data (>20,000 SNPs) and high-confidence archaeological association (see Methods, Supplementary Information S2) for each site. We mark the numbers of previously published ancient individuals with a “*” and depict locations of aDNA context using circles. (**b)** Principal component analysis (PCA). We first computed PCs using 1196 present-day individuals from Western Eurasia and North Africa (Methods). We then projected 128 individuals with >20,000 SNPs covered and confidently assigned to a Phoenician-Punic context (see Methods, Supplementary Table 5) onto the first two PCs. For context, we also projected various Bronze and Iron Age individuals across the Mediterranean (colored circles matching panel a, Supplementary Table 6). Extended Data Figure 1 depicts the PCA projections broken up according to archeological sites and dates. Extended Data Figure 10 depicts a projection of the reference populations only and a zoomed-in version of the Levantine individuals. Abbreviations: BA - Bronze Age, IA - Iron Age, M/N - Meso/Neolithic, L - Late, M - Middle, E - Early.

We screened human skeletal remains from 398 individuals excavated from 14 sites in Iberia, Sardinia, Sicily, North Africa, and the Levant, using in-solution enrichment for more than 1.2 million single nucleotide polymorphisms (SNPs)^22^ and the mitochondrial genome^23^ (Methods, Supplementary Tables 1–2). Sampled sites cover diverse cultural, temporal, and geographic contexts (Supplementary Information S1). They include the Levantine Phoenician settlement of Akhziv, large urban settlements with probable Levantine foundations, such as Cádiz, Motya, and Carthage, and sites with evident cultural ties to Carthage, such as Villaricos or the rural settlement of Kerkouane (Figure 1a). We published results for 210 unique individuals that produced genome-wide data that met minimal standards for aDNA authenticity, of which 196 came from Phoenician and Punic contexts (Supplementary Table 3). We carried out population genetic analyses on a subset of 157 individuals with sufficient data, as described below.

We generated 111 direct accelerator mass spectrometry-based dates on bone for 99 of these individuals (Supplementary Table 4). These radiocarbon dates were crucial to confirming archaeological context since several sampled Punic sites were occupied in later periods and since excavations for a subset of analyzed samples pre-date the establishment of modern archaeological methods (in some cases more than a century ago). Due to fluctuations in the proportion of atmospheric carbon 14 in the period of interest, we could typically assign samples only to around 800–400 BCE (the so-called “Hallstatt Plateau” in the calibration curve used for radiocarbon dating) or 400–200 BCE (Supplementary Information S2). Nevertheless, this allowed us to distinguish within Punic sites between people living during the earlier stages of the Carthaginian expansion (before 400 BCE), people living after this time but still in Punic contexts, and people plausibly living after the transition to Roman control around 250–150 BCE. A significant limitation of our sampling is the absence of sampled individuals who predate 600 BCE (based on archaeological dating of the tombs), because cremation was the dominant form of burial in Phoenician settlements in the central and western Mediterranean before this time^24^ (see Discussion).

We filtered to individuals with higher quality data (>20,000 SNPs and no evidence of substantial contamination) and with verified archaeological context, obtaining a set of 157 individuals for population genetic analysis. Within this dataset, we identified 131 individuals with Phoenician or Punic context, out of which 108 had a firm archaeological context indicating that they can confidently be classified as Phoenician or Punic (Figure 1a, Supplementary Information S2). We added previously published sequences for eight individuals from two Punic sites in Sardinia^20^ and one from Ibiza^21^. A recent study reported whole genome shotgun sequencing data for 12 individuals from the North African site of Kerkouane^19^. We used in-solution enrichment (which enabled us to convert samples with low proportions of endogenous DNA to analyzable data) to generate data for 17 new individuals from Kerkouane and additional data for 10 individuals with previously reported data. We analyzed the data we generated with that from previously reported ancient individuals from other relevant times and regions, as well as 26 newly sequenced individuals from relevant contexts (which are not Phoenician or Punic). Among these are an Early Iron Age individual from the inland northeast Algerian site Khenkela, which we use in our model of North African ancestry, and 20 individuals from originally Punic sites in Sicily, Sardinia, and Iberia whose radiocarbon dates place them within the period of Roman hegemony (Supplementary Information S2).

## Genetic ancestry and admixture modeling

**Extended Data Figure 1:**
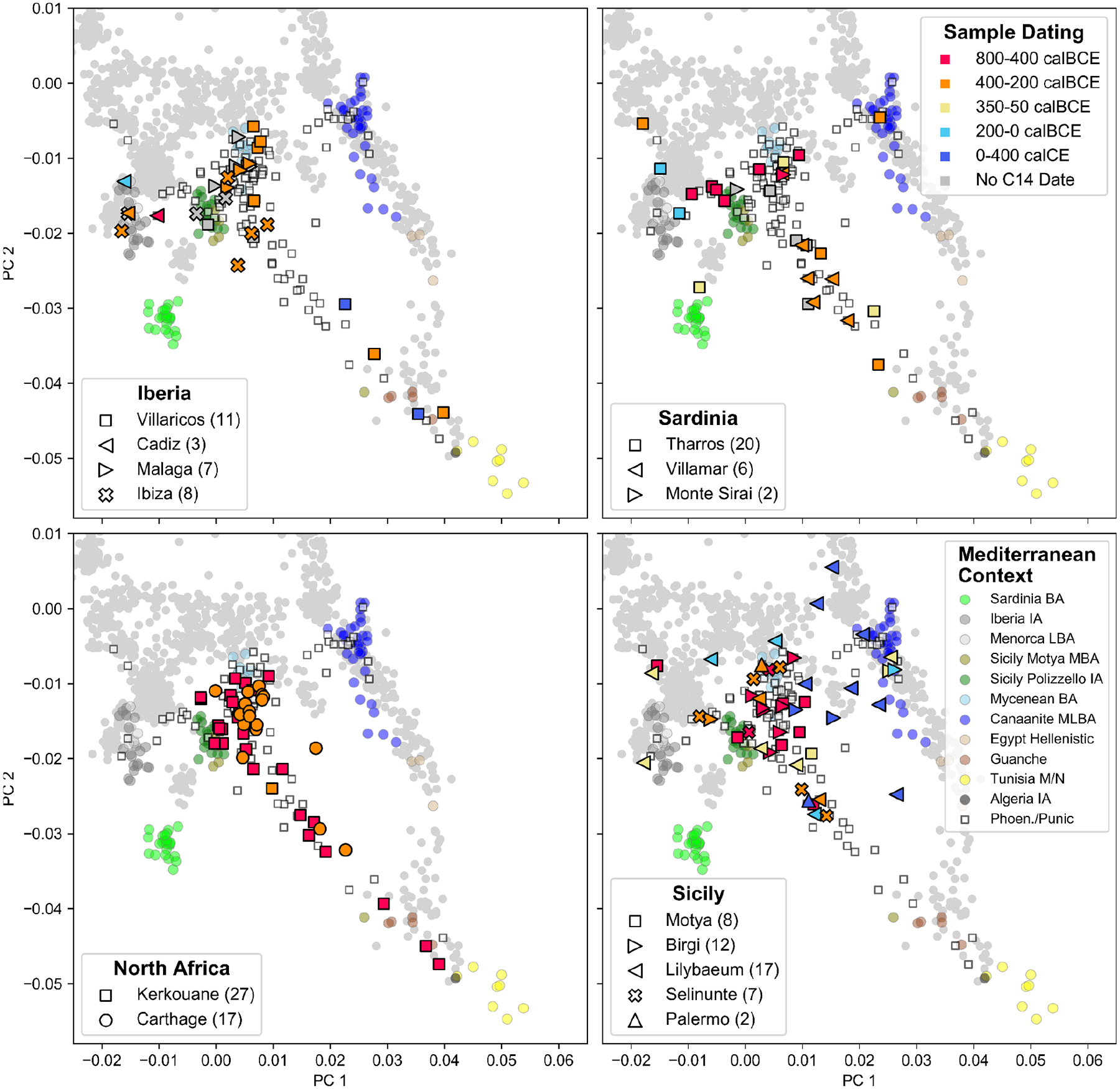
PCA of Punic individuals grouped according to site and chronology. We project individuals sequenced on over 20,000 SNPs onto the same two PCs as in Figure 1 (calculated from modern individuals, gray dots). Here, we plot all individuals from Punic archeological sites, excluding the Phoenician site of Akhziv and including the 20 individuals dated to the Roman period (Supplementary Table 5). We split the sample into panels representing our four major geographic regions: Iberia, Sardinia, North Africa, and Sicily. The shape of the symbols indicates the site (lower left legend), and the color indicates the date range of each individual (as described in the upper right legend).

To explore the genetic variation in our newly reported data, we projected whole genomes from the ancient individuals onto the first two principal components (PCs) computed from 1,196 present-day West Eurasian and North African individuals (Figure 1b, Extended Data Figure 1, Methods). For context, we also projected Bronze and Iron Age individuals from relevant archaeological cultures across the Mediterranean, which broadly cluster based on their geographic distribution. We find that individuals from the Levantine Phoenician site of Akhziv cluster together with previously published Bronze and Iron Age Levantine individuals, including from Megiddo in present-day Israel^25^ and the Phoenician cities of Sidon and Beirut in present-day Lebanon^17,18^. In contrast, individuals from Punic sites in the central and western Mediterranean do not cluster with Levantine individuals. Instead, they are broadly distributed with a primary mode overlapping Bronze and Iron Age individuals from Sicily and the Aegean, regardless of sampled location. In addition to this cluster, a narrow cline of individuals points from this mode towards Northwest African individuals, which include pre-Contact Guanche individuals from the Canary Islands^26^ and the newly sequenced Early Iron Age individual from Khenkela (inland Algeria). This suggests that the Phoenician people of Akhziv derived most of their ancestry from previous populations in the Levant, while the people from Punic sites in the central and western Mediterranean shared recent common ancestry with populations from Sicily and the Aegean, with additional recent admixture with North African populations.

**Extended Data Figure 2:**
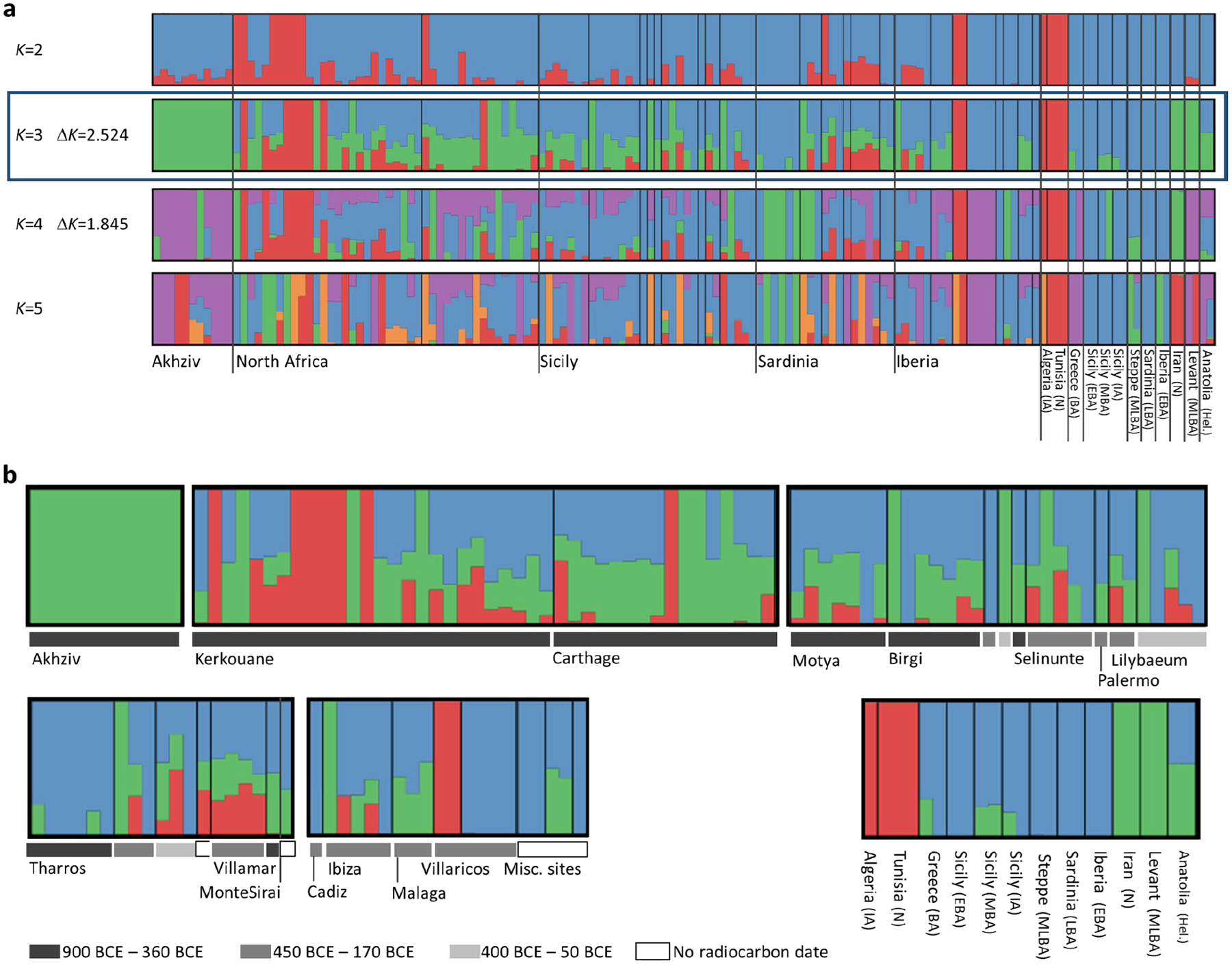
Ancestry models inferred for 122 Phoenician-Punic individuals using unsupervised *ADMIXTURE* with *K*=2, 3, 4 and 5 latent ancestry components. The 122 Phoenician-Punic individuals sequenced for more than 100,000 SNPs were jointly analyzed with 24 individuals from related ancient populations across the Mediterranean (Supplementary Table 12). **(a)** The model with the highest likelihood was obtained for each value of *K* among 50 replicate runs. Individuals are grouped based on region. Values of the Δ*K* score of^58^ are specified for *K*=3,4, with a higher score obtained for *K*=3, suggesting optimal fit. **(b)** A more detailed depiction of the best model obtained with *K*=3 latent ancestry components corresponding to North African ancestry (red), eastern ancestry (green), and central/western Mediterranean ancestry (blue). Individuals are partitioned within each region according to site and time range (see legend). The unsupervised *ADMIXTURE* model does not adequately differentiate between Levantine ancestry and ancestry found in other Mediterranean locations (e.g., Anatolia and Sicily), unlike the *qpAdm* models of Extended Data Figure 3.

**Extended Data Figure 3:**
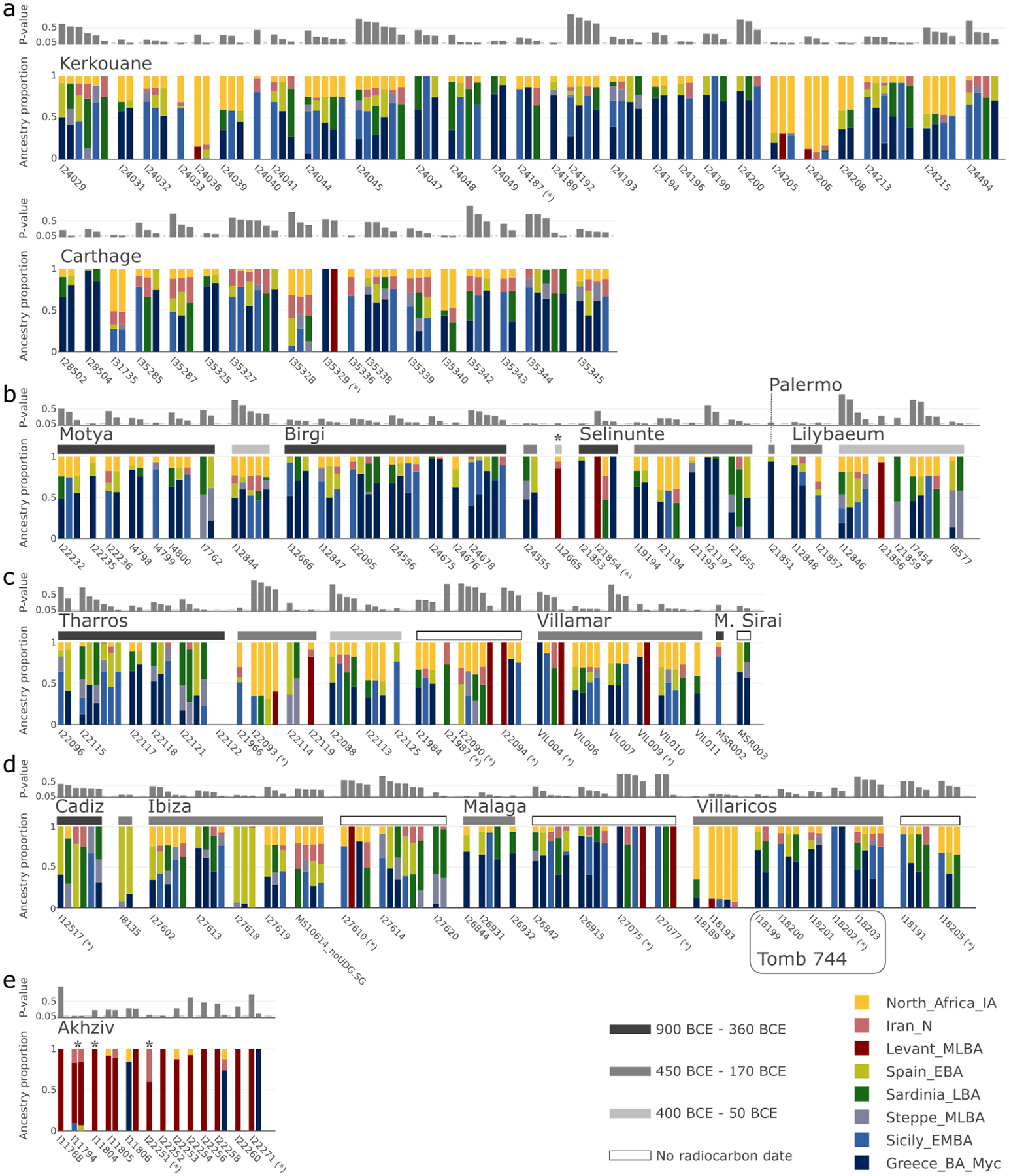
Ancestry models inferred for the 140 Phoenician-Punic individuals in our data set by qpAdm. We partitioned individuals by region: **(a)** North Africa, **(b)** Sicily, **(c)** Sardinia, **(d)** Iberia, and **(e)** the Levant. Within each region, we grouped individuals by site, and for sites in Sicily, Sardinia, and Iberia, also by broad date ranges (see legend for color code). We ordered the models of each individual according to their P-values (gray bar above each model). We report P-values assuming that the LRT statistic is chi-squared distributed with degrees of freedom determined by the number of populations and of contributing source populations. We did not correct these P-values for multiple testing, but this approach is conservative since we report models with comparatively high P-values (those that are not rejected by the test). Individuals with low coverage (fewer than 100,000 SNPs) are indicated by an asterisk (*) next to the sample ID. Eastern ancestry models are indicated by a contribution of the proxy sources Levant MLBA. In contrast, western ancestry models are indicated by contributions from either Greece BA (Myc), Sicily EBA, Sardinia LBA, Iberia LBA, or Steppe MLBA. There are five individuals for whom no valid eastern or western model was inferred. For four of them, we inferred valid models under the broad ancestry scheme (marked by an asterisk above the vertical bar), and for one (I22122 from Tharros, Sardinia), we could not infer any valid model.

**Figure 2:**
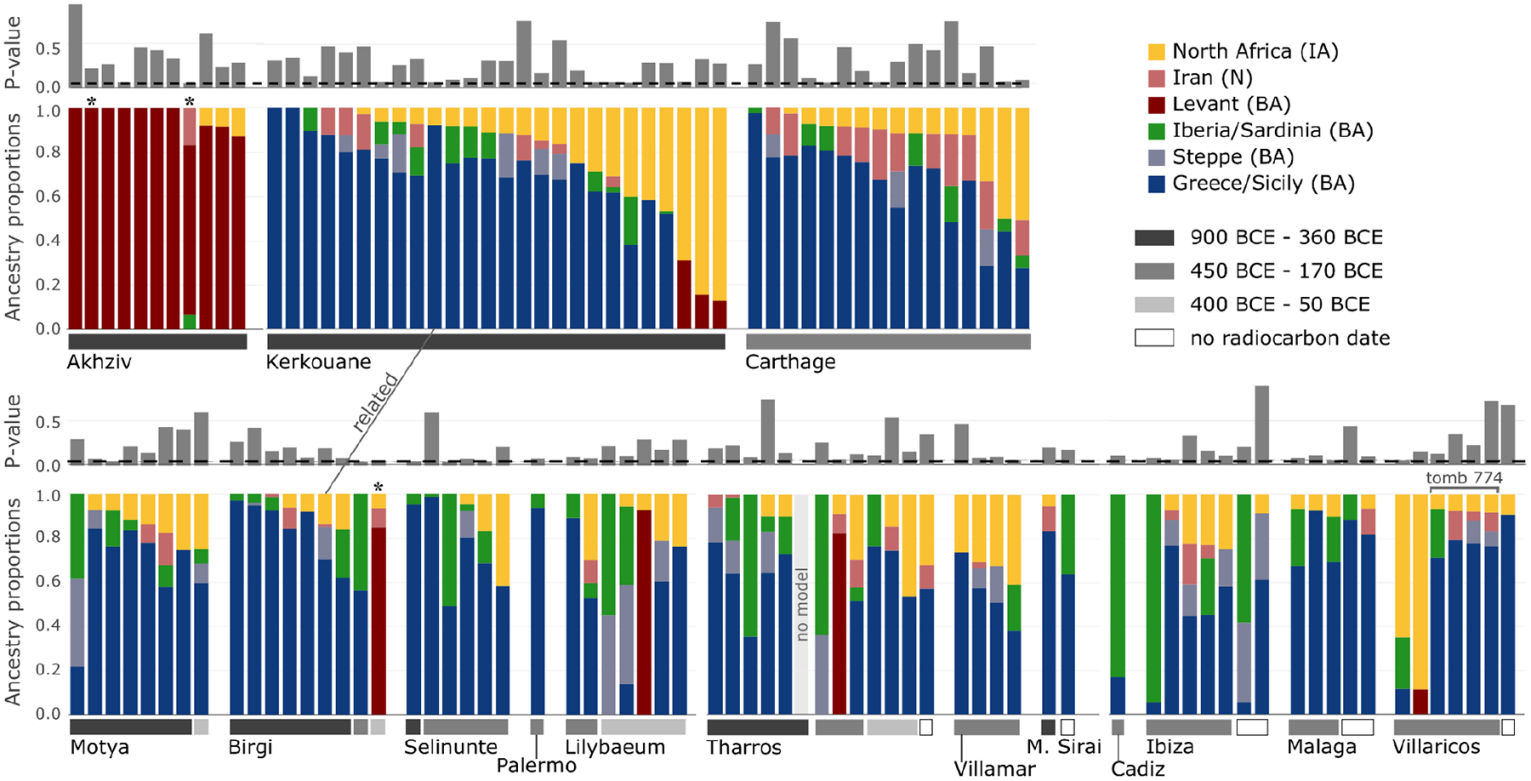
Ancestry models inferred using qpAdm. The figure depicts the representative admixture models for 122 Phoenician-Punic individuals in our data set sequenced at more than 100,000 SNPs. We sorted individuals by region (Levant, North Africa, Sicily, Sardinia, and Iberia), then by site, estimated date range (gray horizontal bars above the site names), and, finally, by inferred North African ancestry. We combined the ancestry proportions inferred for Greece BA (Myc) and Sicily EMBA (Sicilian-Aegean ancestry), and the ancestry proportions inferred for Sardinia LBA and Iberia EBA (western Mediterranean ancestry). We combined these ancestries due to the limited ability of our qpAdm models to distinguish between them (Supplementary Information S3). Since individuals typically had several valid admixture models, we selected the model that maximized Sicilian-Aegean or Levantine ancestry (Methods). For three individuals (marked by an asterisk), we could only fit a broad ancestry model (Methods), and one individual from Tharros could not be fit by any of the ancestry models we considered. The figure highlights a pair of related individuals from Birgi and Kerkouane (see Figure 4) and related individuals buried in the same tomb in Villaricos (see Figure 5). The complete set of valid models for all 140 individuals in the data set (including those with fewer than 100,000 SNPs) is specified in Extended Data Figure 3.

Other methods useful for learning about population mixture (see Methods) confirmed the main hypotheses suggested by the PCA. Using *ADMIXTURE*, we inferred a three-component model with clusters maximized in North Africans, eastern populations (Levant/Iran), and central and western Mediterranean groups (Extended Data Figure 2). Individuals from Akhziv exhibit similar ancestry patterns to those of Bronze Age individuals from the Levant. In contrast, individuals from Punic sites are assigned clusters more typical of Bronze and Iron Age populations from the central and western Mediterranean and North Africa (see Supplementary Information S3). To carry out formal tests of fits for alternative models and to quantify ancestry proportion under the fitting models, we used *qpAdm*^27,28^, with 78 ancient individuals from eight different genetically homogeneous groups as proxies for the ancestry sources (Supplementary Table 7; Methods). While multiple ancestry models could fit most individuals (Extended Data Figure 3), several robust observations emerged. First, all individuals from the Phoeniciean site Akhziv derived more than 80% of their ancestry from a Levantine Bronze Age population (Figure 2). In contrast, only three individuals from Punic sites were assigned substantial Levantine ancestry: two from Sicily (whose radiocarbon dates overlap with the period of Roman control in Sicily) and one from Sardinia, all of whom clustered in the PCA with Levantine individuals (Extended Data Figure 1). The primary source of ancestry in most Punic individuals is best proxied in our analysis using Bronze Age individuals from Sicily and Greece. In every region, including North Africa, and every time period, some Punic individuals derive nearly all of their ancestry from this source. However, our analysis could not more precisely identify the geographic origin of this source population as it could not confidently distinguish between Bronze Age Sicilian and Aegean sources, which had only subtle ancestry differences from each other (see Discussion).

**Extended Data Figure 4:**
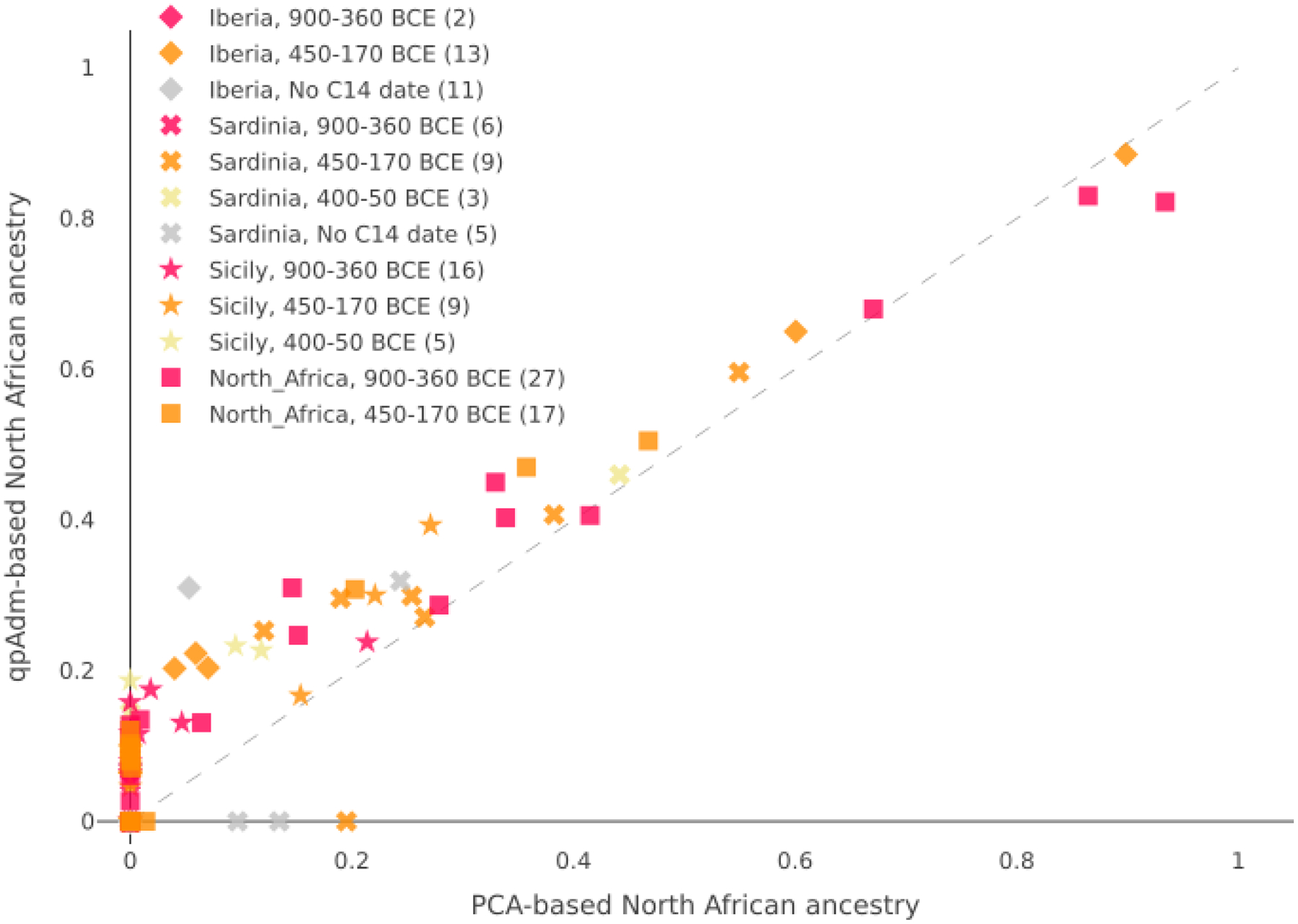
Proportions of North African ancestry inferred using the 2D PCA and qpAdm for 123 Punic individuals. We exclude from this analysis the Akhziv sample, the three individuals from Sicily and Sardinia that cluster near Levantine individuals in the 2D PCA, and one individual for which we could not fit a qpAdm model. The qpAdm estimates are based on the smallest proportions estimated for the individual in a valid qpAdm model (see Methods). The PCA-based estimates of North African ancestry were computed by projecting the location of each sample in the PCA onto a cline from the cluster defined by Bronze Age individuals from Sicily to the cluster defined by North African individuals (see Supplementary Information S3 for more details). The two approaches yield similar estimates, with qpAdm being more sensitive to low ancestry proportions. Individuals from Kerkouane (depicted as squares) appear to have a broad range of North African ancestry (0 – 94%). Individuals from Sicily typically have lower proportions of North African ancestry (<20%), and we observe no significant shift in time. On the other hand, in Sardinia, none of the 12 individuals for which we inferred more than 10% North African ancestry (according to at least one of the two approaches) dated before 400 BCE, suggesting that North African ancestry was likely introduced around that time (Supplementary Information S3). We see a similar pattern in Iberia, but since we only have one individual from Iberia dating before 400 BCE, we cannot confidently infer the absence of North African ancestry during this time.

The second most common ancestry source in Punic sites is consistent with deriving from indigenous North Africans, which we define as a population genetically similar to the Early Iron Age individual from inland northeast Algeria (Extended Data Figure 3 and Supplementary Information S3). North African ancestry appears as early as the 6th and 5th centuries BCE in a substantial subset of individuals from Kerkouane, and sporadically, in low fraction at Sicilian sites (Motya and Birgi), and not at all in sampled Iberian and Sardinian individuals before the 4th century BCE (Figure 2; Extended Data Figures 1 and 4). The rise in North African ancestry after 400 BCE outside of North Africa is particularly striking in Tharros, Sardinia (Extended Data Figures 1 and 3). Although indigenous North African ancestry was widespread after 400 BCE in the Punic world, it remained a minority ancestry component (below 50%) in all individuals except for three from Kerkouane, two from Villaricos, and one from Tharros. Even in North Africa, ten of the 27 individuals from Kerkouane and five of the 17 individuals from Carthage can be modeled with no indigenous North African ancestry, and 84% of individuals from these sites have more than 50% Sicilian-Aegean ancestry, making it the dominant ancestry component also in North African Punic sites (Extended Data Figure 3).

Many individuals in Punic sites in Sicily and North Africa harbored substantial proportions of ancestry consistent with that observed in earlier people from the same geographic regions (this ancestry not necessarily being local). In contrast, we do not observe evidence of a genetic contribution of Bronze Age people of Sardinia and Iberia to individuals in nearby Punic sites either in clustering analysis or in formal modeling analysis (Figures 1b and 2). Only two Iberian individuals, from Ibiza and Cádiz, had confidently high proportions of Bronze Age Iberian ancestry (Figure 2, Extended Data Figure 3). Instead, Punic sites in the western Mediterranean share similar distributions of patterns of ancestry of predominantly Sicilian-Aegean or North African origin (Figures 1 and 2, Extended Data Figure 1; see detailed discussion in Supplementary Information S3).

## Genetic heterogeneity and connectedness

Five lines of population genetic analysis reveal that individuals in Punic sites were part of a genetically heterogeneous and interconnected Mediterranean population.

**Extended Data Figure 5:**
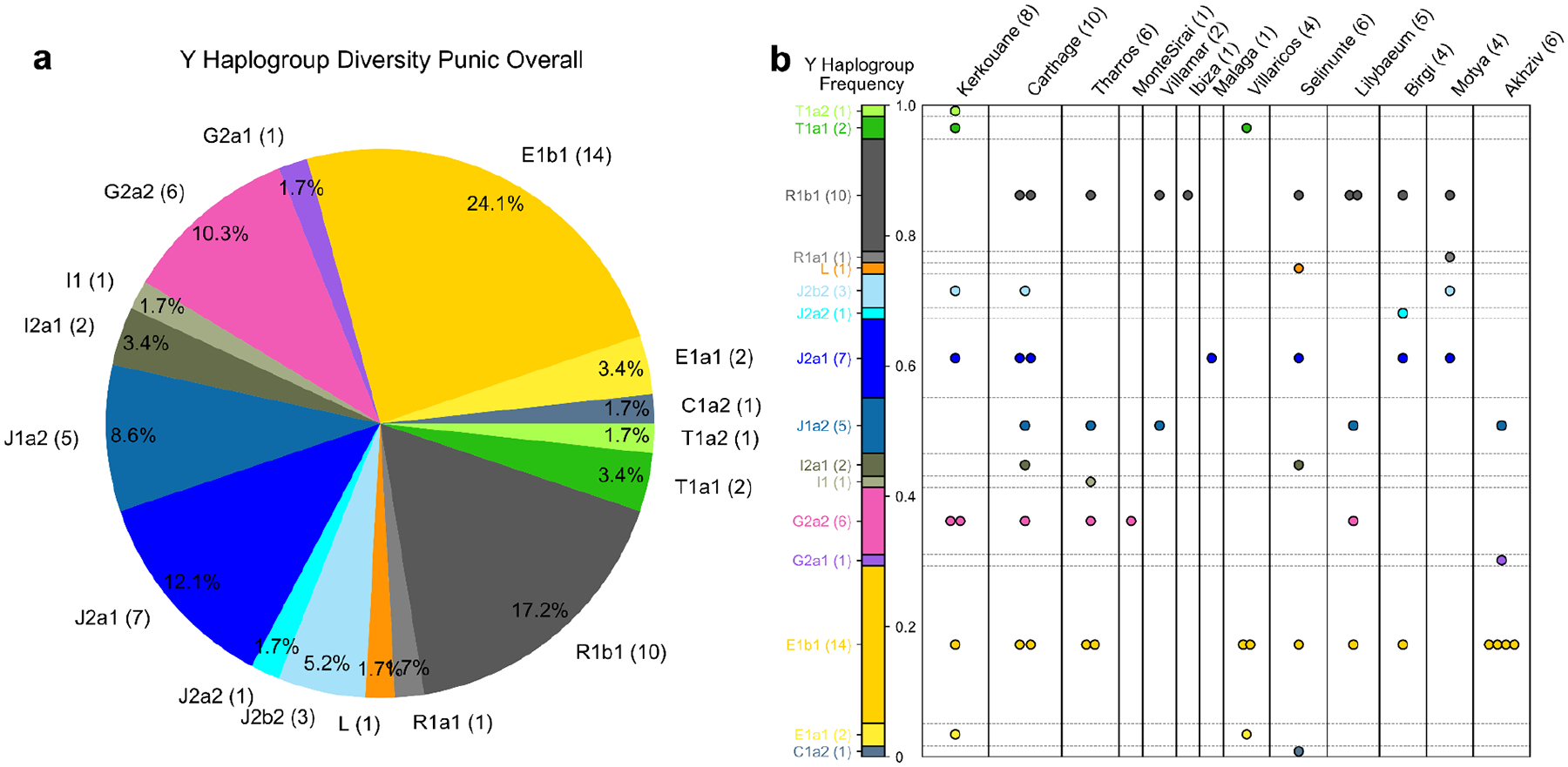
Y Haplogroup Diversity in male individuals from Phoenician and Punic contexts. We inferred the first four characters of the ISOGG 2019 Y haplogroup classification for all Phoenician and Punic males with more than 100,000 autosomal SNPs covered (as those in almost all cases have sufficient coverage on the Y chromosome; see Methods). **(a)** Pie chart of Y haplogroup frequencies. **(b)** We visualize the Y Haplogroup diversity partitioned per Phoenician or Punic site and denote each individual’s haplotype by one circle. We set the height of the bar to the overall frequency (as depicted in panel **a**). The numbers in brackets indicate the total Y haplotype sample size.

**Figure 3:**
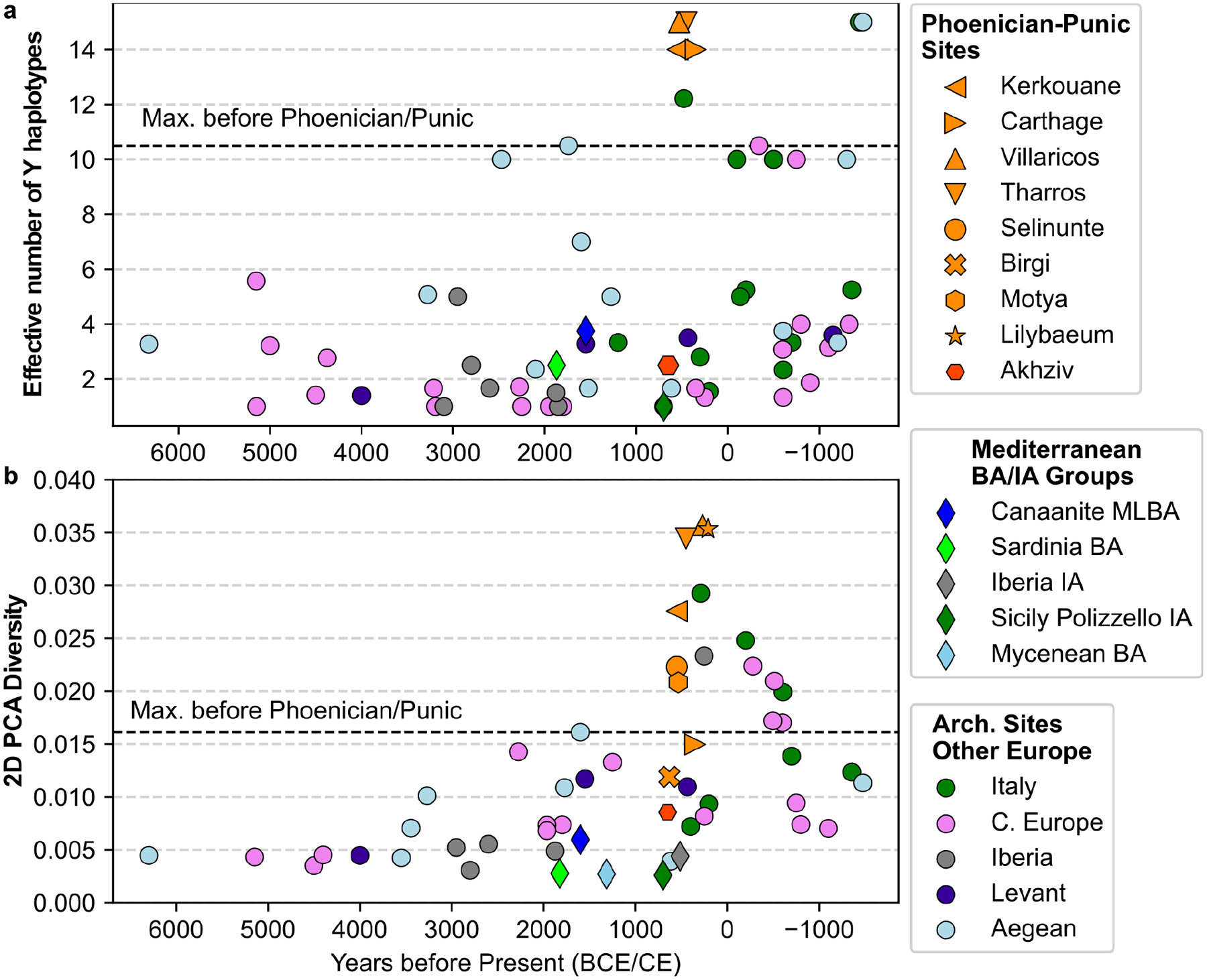
Autosomal and Y Diversity per site in Phoenician-Punic contexts and the published aDNA record. (**a)** We calculated the Y haplogroup diversity for contexts with at least five males with sufficient data using Y haplogroups at the level of the first four characters of the ISOGG classification and the Inverse Simpson index, also known as the effective number of types (see Methods). **(b)** We calculated the 2D PCA diversity for contexts with at least ten individuals with sufficient data using the first two PCs from Figure 1 and the mean pairwise distance of those coordinates (see Methods, values listed in Supplementary Information S5). The dashed horizontal bar in both panels indicates the maximum diversity (autosomal or Y-based) observed in sites before 500 BCE. The Mediterranean Bronze Age (BA) and Iron Age (IA) groups (diamonds) correspond to the ones depicted in Fig. 1. In each of these five groups, we included individuals from different archaeological sites.

First, we observe a substantially higher Y haplogroup diversity per archaeological site compared to earlier people (Figure 3a; Extended Data Figure 5). In each sampled Punic site, most males have differing Y haplogroups (using the first four letters of the ISOGG classification, see Methods). Overall, no single lineage dominates the sample across sites, including most commonly E1b (14 out of 58 individuals), R1b (10), J2a (8), G2a (7), and J1a (5). The high Y chromosome diversity is notable in light of the prior proposal of J2 (J-M172) as a footprint of Phoenician demographic expansions and their genetic legacy today^29^. We observe J2 haplogroups in only 20% of the Punic males, and at a similar low frequency in Levantine males from the Bronze and Iron Age and at even higher frequencies in Bronze Age Aegean and Minoan populations (J2a) and Bronze and Iron Age Balkan populations (J2b) (see Supplementary Information S4). This signal challenges the hypothesis that J2 is a haplogroup specifically diagnostic for Phoenician expansions from the Levant. Instead, the high chromosome Y diversity suggests that no single Y haplogroup can serve as an effective marker for Phoenician expansion.

**Extended Data Figure 6:**
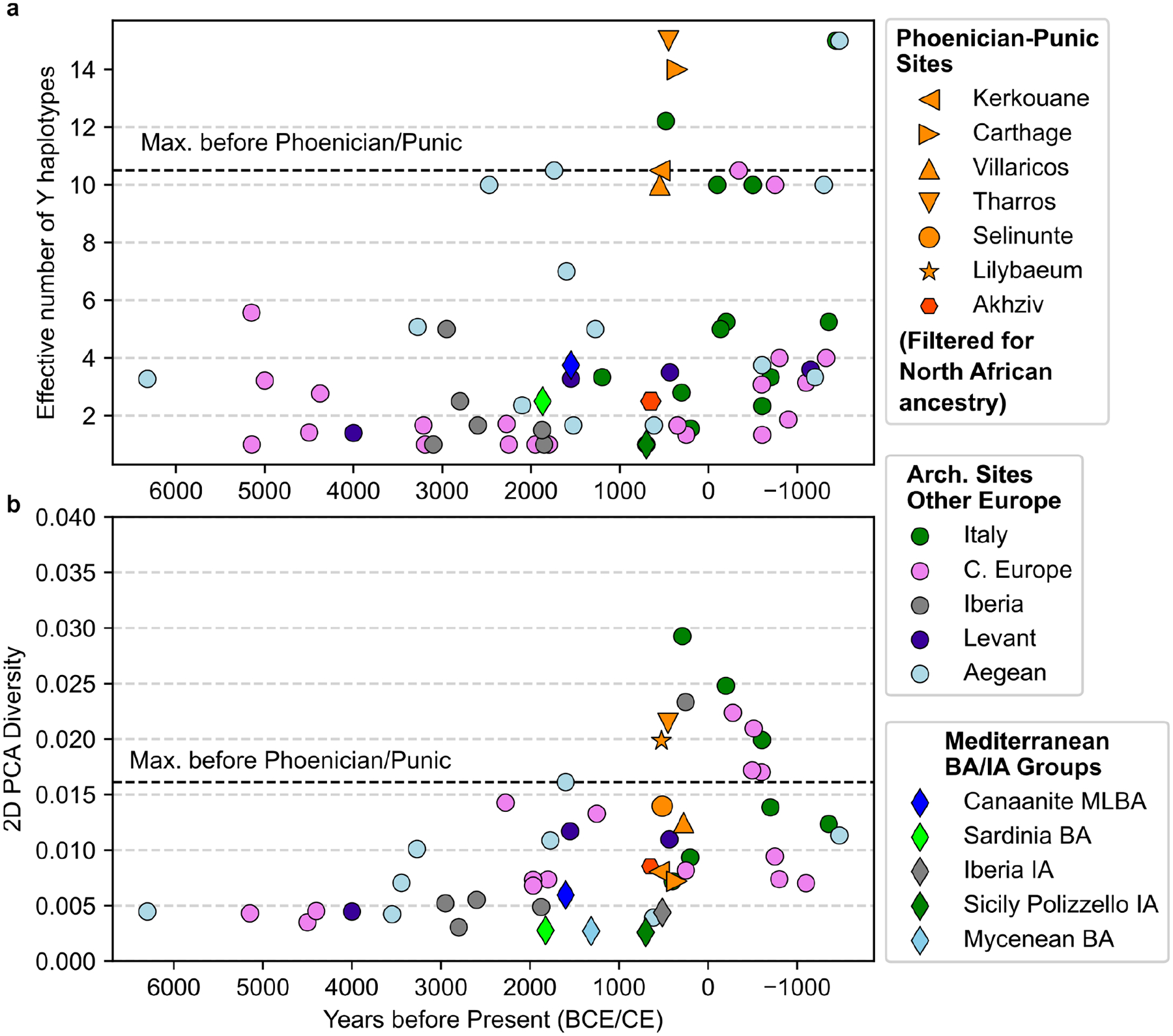
Autosomal and Y Diversity without the contribution of filtered North African ancestry, per site in Phoenician-Punic contexts and the published aDNA record. **(a)** Y haplogroup diversity measured using the Inverse Simpson index. This value is computed as in Figure 3a, excluding the three Punic individuals (from Kerkouane, Villaricos and Selinunte) with distinct North African Y haplogroups E1a and L (see Extended Data Figure 5). **(b)** autosomal diversity measured using the first two PCs from Figure 1 and the mean pairwise distance of those coordinates. This value is computed as in Figure 3b, excluding individuals with more than 10% North African ancestry based on qpAdm in Phoenician-Punic sites (see Extended Data Figure 4). Here, we combined individuals from the nearby Sicilian sites of Birgi, Motya, and Lilybaeum into one group (labeled Lilybaeum here). In both panels, the diversity measures for the context populations are as in Figure 3 (without any additional filtering), and the dashed horizontal bar in both panels indicates the maximum diversity observed in sites dating before 500 BCE. See Supplementary Information S5 for a more detailed description of this analysis.

Second, we observe high heterogeneity in genome-wide ancestry. The genetic variation in most Punic sites, as measured by the diversity of their positions in the PCA (see Methods), is higher than in earlier sampled sites from all across the Mediterranean locations since the 5th millennium BCE (Figure 3b). While varying North African ancestry is a primary driver of the high diversity we observe in Punic sites, Y haplogroup diversity levels in several sites remain higher than in earlier populations, even when this specific contribution is factored out (Extended Data Figure 6 and Supplementary Information S5).

Third, our genetic data document that the individual ancestry variation was high within Punic sites, but this variation was overall similar between different regions, namely Sardinia, Iberia, Sicily, and North Africa (Figures 2 and Extended Data Figure 1). The most parsimonious explanation for this overlap is that populations in different Punic sites drew from the same (or very similar) ancestry sources and had a high degree of interconnectedness, as it is unlikely that such a similar pattern of ancestry variation across individuals would be observed in geographically distant regions with differing local ancestries unless they shared a common ancestral source.

**Extended Data Figure 7:**
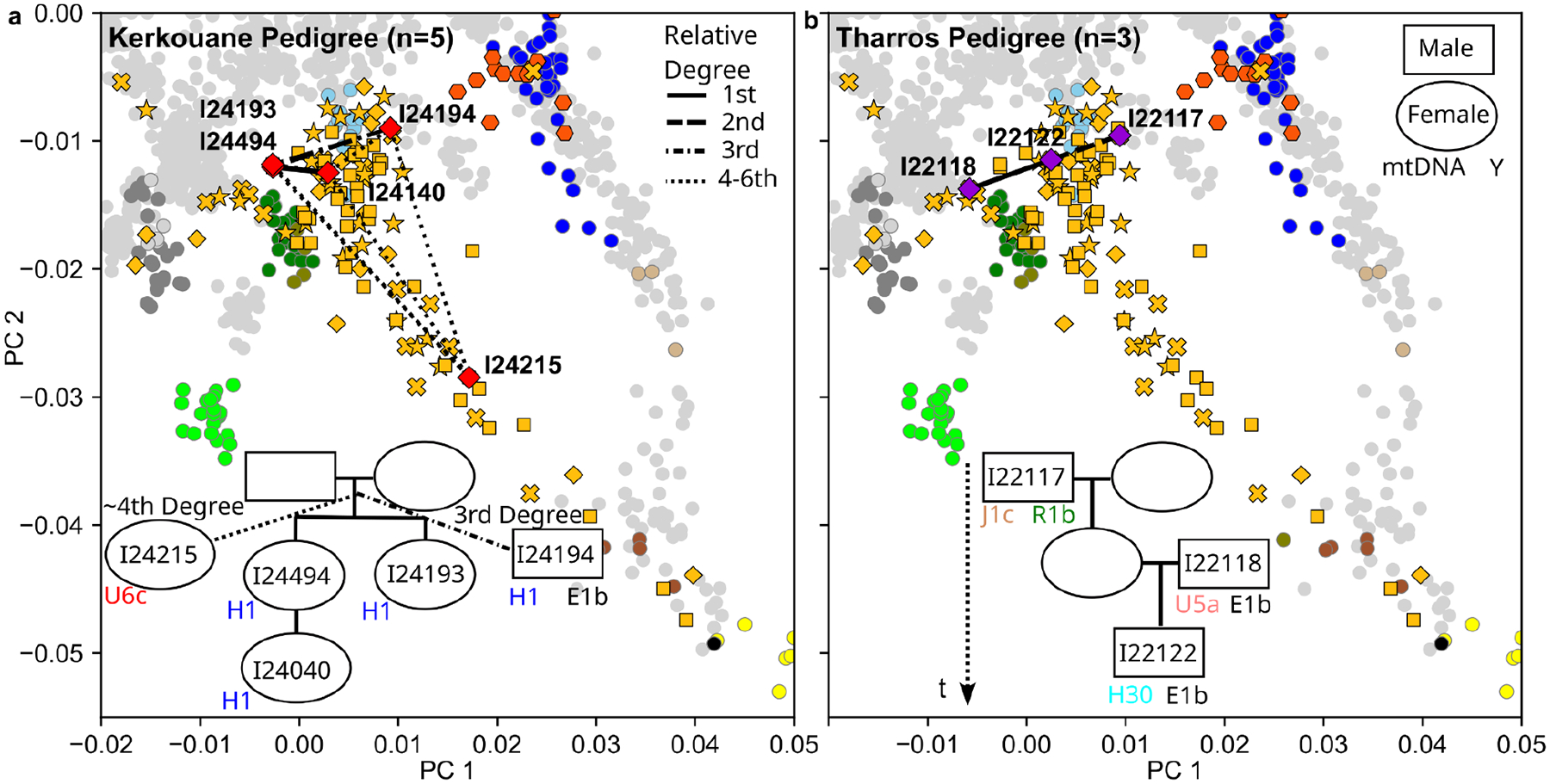
Two reconstructed pedigrees of Punic individuals. We reconstructed two pedigrees based on inferring biological relatives with pairwise kinship (using IBD segment sharing) and uniparental haplogroups: **(a)** A pedigree linking five individuals from Kerkouane, North Africa; **(b)** A pedigree linking three individuals from Tharros, Sardinia. In the Kerkouane pedigree in (a), individuals I24215 and I24194 are inferred to be 3rd-4th degree relatives of the two siblings I24494 and I24193, but the exact pedigree relationship cannot be resolved. Each panel depicts the projection of the related individuals onto the two major PCs used in Figure 1. Each pedigree specifies the sample IDs for all individuals, the mitochondrial (maternal) haplogroup and the Y (paternal) haplogroup for males. Both pedigrees contain individuals dating to 800–400 calBCE and link several individuals via the maternal lineage: We infer four identical maternal haplogroups in Kerkouane and a maternal grandfather in Tharrosーtwo observations that are inconsistent with strict patrilocality.

Fourth, we find evidence for mixing of people with different genetic backgrounds within extended pedigrees. We used long genomic segments that are identical by descent (IBD) among pairs of individuals to screen for biologically related individuals. In particular, multiple shared long IBD segments indicate biological relatives up to the 5th-7th degree, and we could use this to infer a pedigree of five individuals from Kerkouane and a pedigree of three individuals from Tharros (Extended Data Figure 7). Both pedigrees revealed extended families that integrated individuals with diverse ancestries, showing that ancestry mixing was ongoing in some Punic populations.

**Figure 4:**
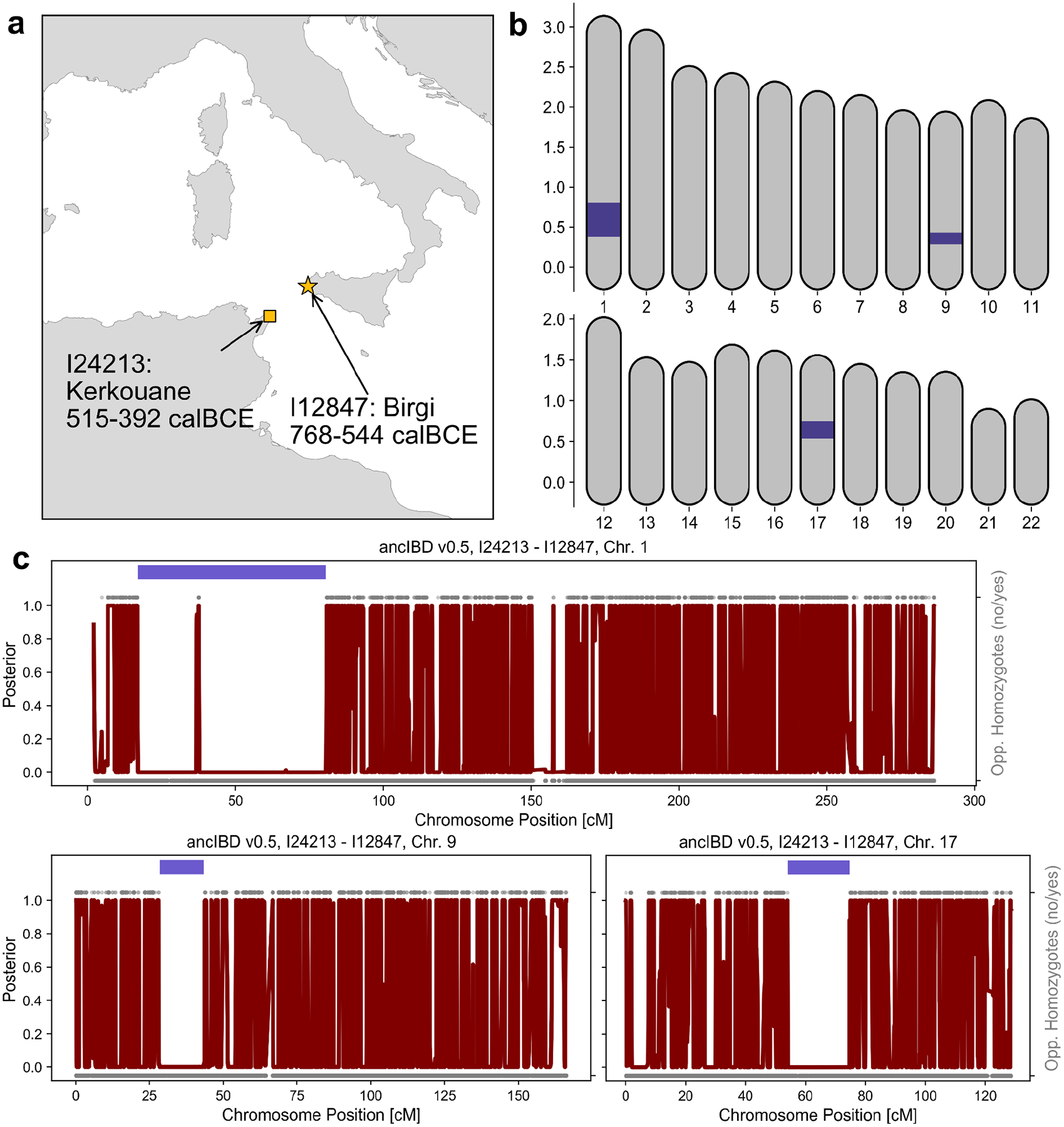
IBD segments shared between individuals from Sicily and North Africa indicate they were 5–7th degree biological relatives. **(a)** The sampling locations and radiocarbon date estimates of the two individuals. **(b)** Genomic location of the position of three long IBD segments on the 22 autosomes. **(c)** Posterior of not being in IBD along chromosomes 1,9, and 17 as calculated with *ancIBD* (red). We also visualize opposing homozygotes (upper versus lower points: yes or no) for all SNPs where the genomes of the two individuals have an imputed genotype posterior probability greater than 0.99. The dark blue segments indicate the inferred IBD segments. Our analysis suggests that both individuals had ca. 85% Sicilian-Aegean ancestry and 15% North African ancestry (highlighted in Figure 2). We list all pairs of related Punic individuals in Supplementary Table 8, including five additional pairs linking sites separated by the Mediterranean Sea.

Fifth, we document multiple pairs of genealogical relatives separated by the Mediterranean Sea. We identify 31 pairs of individuals who share an IBD segment of length >16 cM, which indicates a shared ancestor likely within the last 20 generations (Supplementary Table 8; see Methods). Most pairs (21 of 31) are from within the same site (including the two pedigrees above) or two adjacent sites, such as Birgi and Motya, but ten were pairs of individuals from two distinct sites. These sites are typically close to each other and within the same geographic region (such as Málaga and Villaricos or Birgi and Selinunte). However, in six cases, we find IBD sharing across the Mediterranean, between an individual from Birgi or Motya (in Sicily) and an individual from Kerkouane or Carthage (North Africa), or Malaga (Iberia). This IBD sharing indicates a high enough rate of maritime mobility between Punic communities that we could observe it multiple times in our dataset. This is particularly striking since the Mediterranean Sea is a geographic barrier that maintained high levels of genetic differentiation between populations in Sicily and North Africa before Punic expansion and also in modern groups. One individual from Birgi and one individual from Kerkouane shared three long IBD segments, implying that they were 5th-7th-degree relatives, such as second to third-degree cousins (Figure 4; see Methods). Thus, these individuals or their immediate ancestors traveled at least once between North Africa and Sicily, making this relative pair a concrete case of population interconnectedness.

## Sporadic endogamy within Punic sites

**Extended Data Figure 8:**
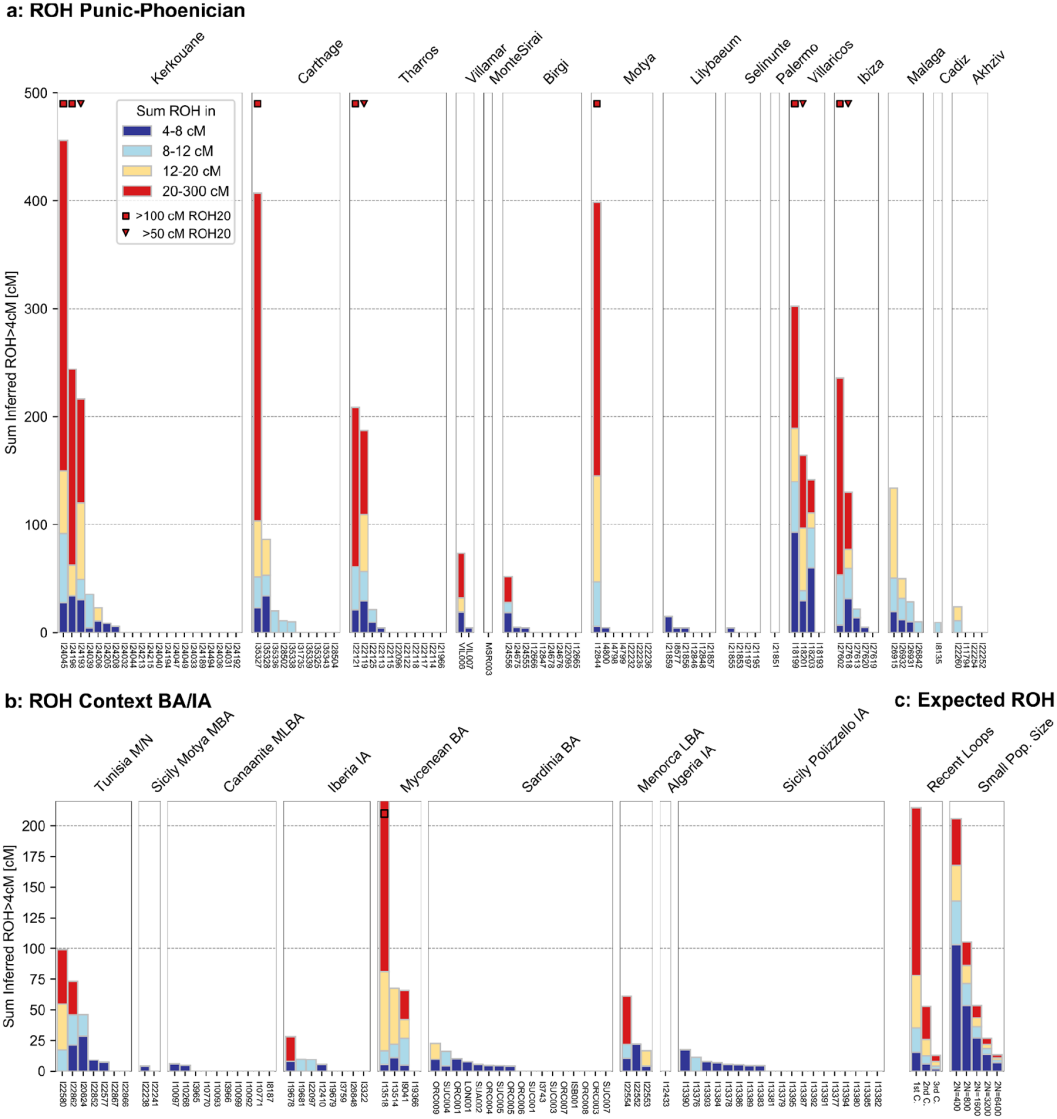
Runs of homozygosity inferred in Phoenician and Punic individuals and ancient individuals of relevant Bronze and Iron Age contexts. We computed runs of homozygosity (ROH) in all individuals with more than 400,000 SNPs covered and recorded the total length (in cM) of ROHs binned by length into four categories (see legend). We label individuals with at least 50 and 100 cM of their genome in long ROH (> 20cM) with triangle and square marks as in^30^ - to indicate offspring of close biological parental relatives. **(a)** ROH in Phoenician and Punic individuals, grouped by site. **(b)** ROH in individuals from relevant Bronze and Iron Age contexts (as depicted in Figure 1). **(c)** Expected ROH for offspring of various cousin matings (according to the degree of relation between parents) and for individuals sampled in populations with small effective size (calculated as described in^30^).

By analyzing Runs of Homozygosity (ROHs) in an individual genome, we could measure the relatedness of the individual’s parents and quantify the frequency of endogamy within Punic sites. Among the 90 individuals in our dataset with adequate data for such inference (see Methods), we found eleven that have more than 50 cM of their genome covered by ROHs >20 cM long (Extended Data Figure 8a), indicating parental relatedness on the level of first or second cousins^30–32^. Three individuals from Kerkouane, Carthage, and Motya even had >350 cM of their genome covered by ROHs, suggesting their parents were second-degree relatives (such as niece-uncle or half-siblings). This rate of detected close-kin unions (11 out of 86 analyzed genomes) is higher than that observed in almost every other Bronze and Iron Age Mediterranean context (Extended Data Figure 8b), except for Late Bronze Age “Mycenaeans” who had similarly elevated rates of close kin unions^33^. The fact that we find cases of close parental relatedness for individuals from six different Punic sites in all four sampled regions points to a geographically widespread practice of mating between close relatives. However, the overall low levels of ROHs in the large majority of analyzed individuals imply that this practice remained sporadic in most regions (with the possible exception of Iberia; Extended Data Figure 8a), and indicates a large recent effective population size, incompatible with the hypothesis that a substantial fraction of the ancestry of Punic people derived from populations that had experienced an intense founder event^30,31^.

**Figure 5:**
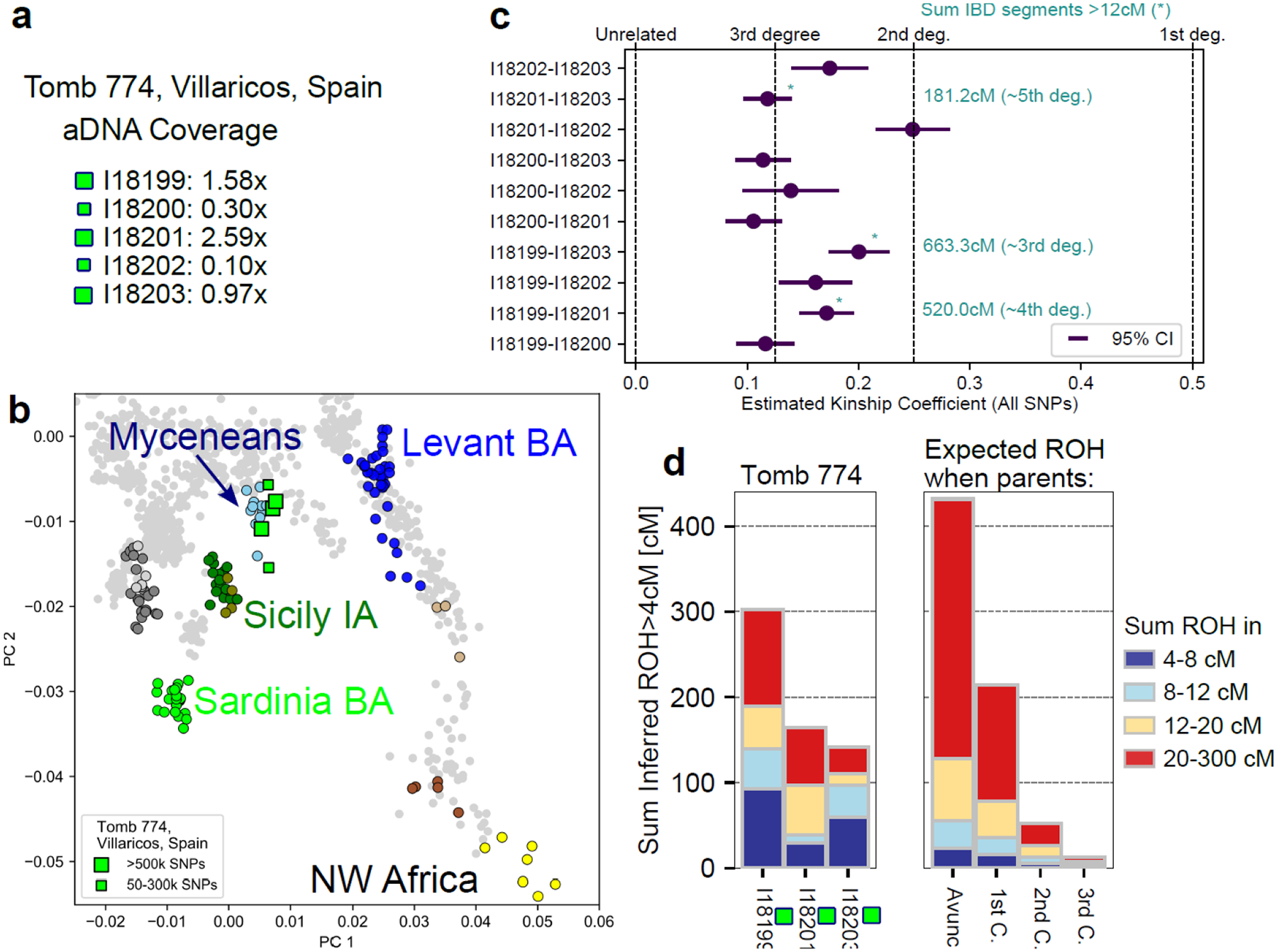
A Punic tomb from Villaricos containing remains of an endogamous community. **(a)** We show the mean sequencing depths for five individuals from tomb 774 in Villaricos (Iberia) for which we obtained aDNA (we indicate coverage > 0.9x by large squares). **(b)** This PCA plot (as in Figure 1) shows that the five individuals cluster close to Bronze Age Mycenaeans. **(c)** We depict the estimated kinship coefficients and their 95% confidence intervals (calculated using average pairwise mismatch rates) for all ten pairs of individuals (Methods), revealing a pattern of second-to-third-degree relatives. For the three individuals sequenced at sufficient coverage for IBD analysis, we specify the total length of long IBD segments (>12cM) shared by the three pairs, confirming that those pairs were closely related. **(d)** The three individuals with high coverage exhibit exceptionally high levels of ROH, close to the level expected for the offspring of first cousins.

A case study that provides insight into social practices among people in Iberian Punic sites who had offspring with their close relatives comes from Tomb 774 in Villaricos, which housed the remains of 18 individuals. We produced viable sequence data for five individuals and dated them to the 5th-4th centuries BCE (see Supplementary Information S2). Our analysis shows that these five individuals cluster on the PCA with the primary mode of Punic ancestry (Sicilian-Aegean ancestry) and near Bronze Age Mycenaeans (Figure 5b). The five individuals were related to each other. Every pair exhibits an average kinship coefficient typical of third to second-degree relatives. For the three individuals with sufficient sequence coverage we directly observed close relations via multiple long IBD segments (Figure 5c). Moreover, we identified exceptionally high levels of long ROH in the three individuals with sufficient coverage (Figure 5d), indicating that their parents were close biological relatives. These observations suggest that the individuals in Tomb 774 were part of an inter-mating community of Sicilian-Aegean ancestry within the diverse Punic community of Villaricos. This tomb exhibits a unique material culture, including painted ostrich eggs typical of Punic funerary contexts and an ivory plaque depicting an Ionian-style capital associated initially with Greek themes by^34^. However, there is no compelling reason to believe the latter indicated the adoption of Greek cultural traditions, since Ionian capitals were a frequent feature of Punic iconography, and ivory working was a Phoenician-Punic specialization. This specific community likely extended beyond this particular tomb in Villaricos, as we found multiple short IBD segments shared between individuals from Tomb 774 and individuals from Málaga, who also exhibit an excess of ROHs and have similar ancestry patterns (Figure 2, Extended Data Figure 8a, Supplementary Table 8). These patterns contrast with the two reconstructed pedigrees in Kerkouane and Tharros (Extended Data Figure 7), which provide examples of extended families in Punic sites incorporating diverse ancestries, highlighting the diverse social practices in Punic sites.

## Discussion

Previous analyses of aDNA from Ibiza and Sardinia hypothesized a significant proportion of eastern Mediterranean ancestry in Punic sites^20,21,35^. While a study of people of Kerkouane in North Africa^19^ did not find evidence of significant Levantine ancestry, it could not make a more general statement because it focused on a single site. Our analysis of an order of magnitude larger sample size from five times more Punic sites rules out significant Levantine ancestry in all 21 previously published genomes and more than 80 newly sequenced individuals, including from prominent urban sites with evidence of Canaanite-Phoenician culture like Carthage, Motya, and Cádiz. Our diverse sample also allows us to trace the expansion of North African ancestry in Punic sites beyond case examples of previous studies^19,20^, showing how, after around 400 BCE, North African ancestry spread to Sardinia and Iberia and suggesting that expanding Carthaginian influence facilitated this spread.

Our results suggest that the Punic demographic expansion was primarily driven by the spread of people with Aegean-like ancestry. We conclude this because the dominant ancestry observed in our Punic dataset matches the ancestry observed in Bronze Age Aegean and Sicilian populations, and Bronze and Iron Age Sicilians in turn derived a large proportion of their ancestry from Aegean populations due to expansions in the Middle to Late Bronze Age and Iron Age^36^. The proximate origin of the Sicilian-Aegean ancestry in Punic people is difficult to resolve due to the low differentiation between Bronze Age Sicilian and Aegean populations (F_ST_~0.01; Supplementary Information S6) and because of sparse aDNA sampling in the Bronze and Iron Age eastern Mediterranean, particularly on the coast of Asia Minor and Cyprus. One possibility is that Aegean-like ancestry originated from early interactions of Phoenicians in Sicily with indigenous Sicilian populations harboring such ancestry derived from earlier Bronze Age gene flow. Another possible source could be interactions between Punic people and Greek colonies established in the central Mediterranean and eastern North Africa (Cyrenaica) since the 8th century BCE. The proximity of Phoenician-Punic settlements in Sicily to some of these Greek colonies, such as Himera and Selinunte, would have provided further opportunities for gene flow.

A critical question raised by our results is how and when Canaanite-Phoenician culture and language were adopted by people without any detectable Levantine ancestry. One hypothesis is that after Levantine Phoenicians founded settlements in the central and western Mediterranean in the early 1st millennium BCE, these communities continuously incorporated people with Sicilian-Aegean ancestry. As a result, most individuals living in these Punic settlements in the 6th century BCE or later would not have had detectable levels of Levantine ancestry. Cremation, the dominant funerary practice among Phoenician communities in the central and western Mediterranean before the 6th century BCE, makes obtaining viable samples for aDNA sequencing difficult, and we consequently do not have data from this period. The shift from cremation to inhumation as the preferred burial practice in the mid to late 6th century BCE is nevertheless also a cultural transition that could reflect substantial numbers of new people integrating into these communities^24^.

**Extended Data Figure 9:**
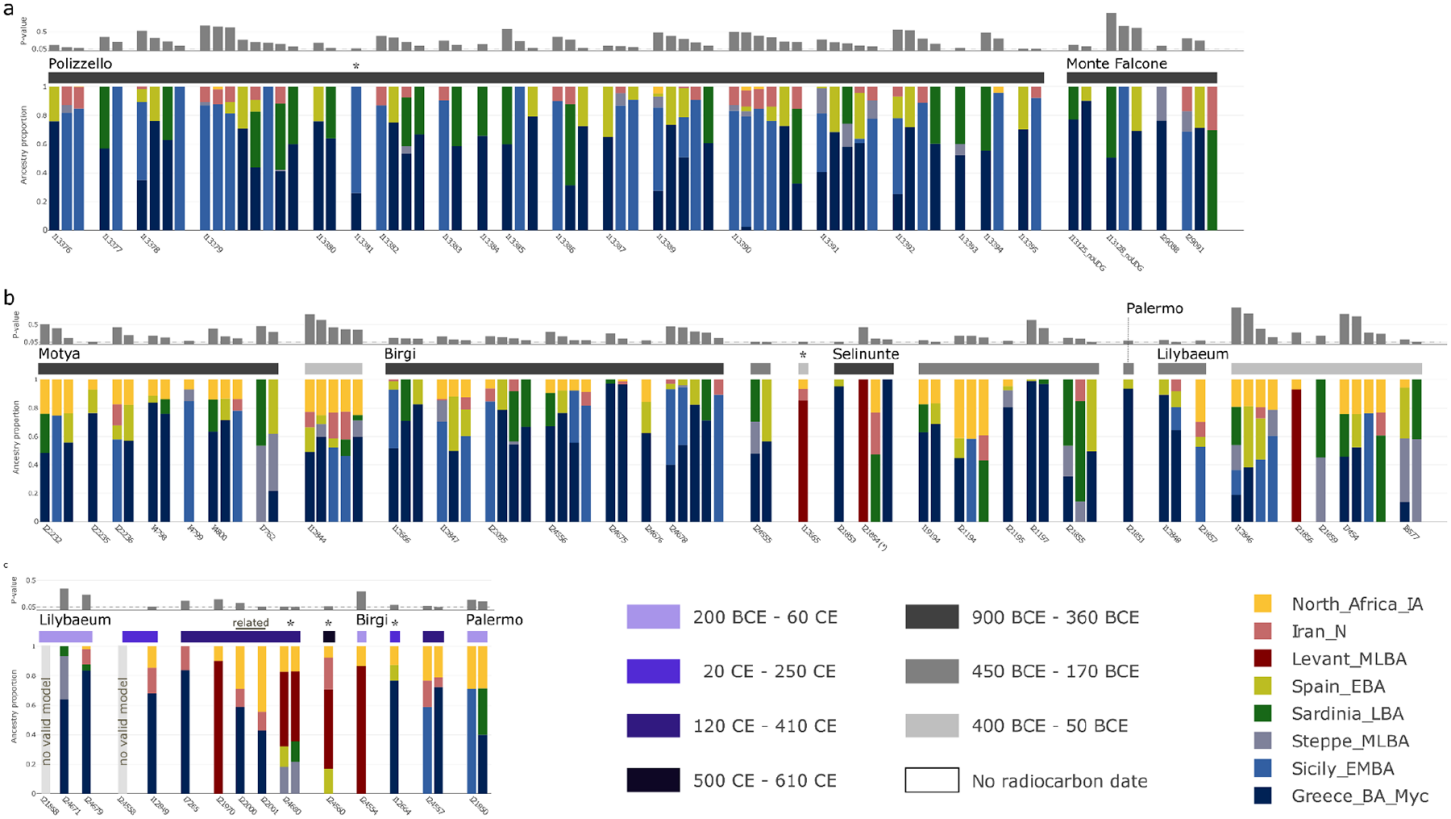
Ancestry models inferred using qpAdm for individuals from Sicily from **(a)** the indigenous Iron Age sites of Polizzello and Monte Falcone, **(b)** from Phoenician sites before Roman expansion (as shown in Extended Data Figure 3b), and **(c)** from Punic sites after Roman expansion. Color horizontal bars indicate radiocarbon dates. The models of each individual are sorted according to their P-values (gray bar above each model). We report P-values assuming that the LRT statistic is chi-squared distributed with degrees of freedom determined by the number of populations and of contributing source populations. We did not correct these P-values for multiple testing, but this approach is conservative since we report models with comparatively high P-values (those that are not rejected by the test). Eastern ancestry models are indicated by a contribution of the proxy source Levant MLBA. In contrast, western ancestry models are indicated by contributions from either Greece BA (Myc), Sicily EBA, Sardinia LBA, Iberia LBA, or Steppe MLBA. There are seven individuals for which no valid eastern or western model was inferred. We inferred valid models under the broad ancestry scheme (marked by an asterisk above the vertical bar) for five of them. Two individuals were inferred to be related through IBD-sharing and are indicated in the figure. The analysis suggests that indigenous populations in Sicily have similar ancestry patterns as observed in the Phoenician sites but without North African ancestry. In later periods, we see the introduction of diverse ancestry sources (Levantine and western Mediterranean), likely associated with the Roman expansion into Sicily. See Supplementary Information S3 for more details.

Finally, the long-term demographic impact of the ancestry we have documented here remains to be clarified. Our data from Roman-era individuals from Sicily (Extended Data Figure 9c) suggests that ancestry patterns shifted toward the eastern Mediterranean, as previously observed elsewhere in the Roman Mediterranean^37–39^. Still, they only partially replaced the ancestry profile from the Punic period in Sicily. Studies that analyze Roman, Byzantine, and Early Medieval material from Sicily, North Africa, Iberia, and Sardinia, carried out in close collaboration between geneticists, archaeologists, and historians, are critical to understanding these transitions that lastingly shaped the Mediterranean.

## Methods

### Laboratory Work, Sequencing, and Quality Control

In clean rooms where the goal was to protect the analyzed samples from contamination by environmental DNA, we sampled bones (especially petrous bones^40^ and teeth, typically aiming for 37 milligrams of powder per sample. We extracted DNA using a methodology optimized to retain smaller DNA fragments^41,42^. We applied uracil-DNA-glycosylase (UDG) treatment to reduce characteristic aDNA errors^43,44^ and then built double- and single-stranded libraries^45^. We enriched these libraries in solution for more than one million SNPs^22,46^ and the mitochondrial genome^23^. We sequenced the enriched products aiming for roughly 30 million paired sequences each. We also sequenced on the order of a couple of hundred thousand sequences for the unenriched libraries.

To analyze the data bioinformatically, we assigned sequences to individuals based on the associated barcodes and indices that we attached to them during library preparation and merged sequences that overlapped by at least 15 bases (allowing up to one mismatch) using a modified version of *SeqPrep* 1.1 (https://github.com/jstjohn/SeqPrep). At overlapping bases, we used the allele call from the higher quality base. We restricted our analysis to sequences of at least 30 base pairs in length, which aligned with a minimum mapping quality of least 10 to either the inferred ancestral mitochondrial genome sequence^47,48^ or the human reference genome sequence (hg19) (https://www.internationalgenome.org/category/grch37/), using the same command from BWA^47^. We removed duplicate sequences based on aligning to the same locations and having the same in-line barcode information. To reduce the effects of characteristic aDNA damage, we trimmed UDG-treated sequences by two base pairs on either end and non-UDG-treated sequences by ten base pairs on either end. We then represented each targeted position covered by at least one sequence with a base quality of at least 20 at that location by randomly selecting a single sequence (‘pseudo-haploid genotyping’).

We built a mitochondrial DNA consensus sequence using *bcftools* (https://github.com/samtools/bcftools) and *SAMTools*^49^, only analyzing sites with a minimum of two-fold coverage and determining allelic status by majority rule. We used *Haplogrep2*^50^ and the *phylotree* database (mtDNA tree build 17) to determine the mitochondrial haplogroup.

To assess evidence of aDNA authenticity, we estimated a 95% confidence interval for contamination on the mitochondrial genome based on the mismatch rate to the consensus sequence, using *contamMix-1.0.1051*^23^. To estimate a 95% confidence interval for contamination on the X chromosome in males (who should have no variation on the non-pseudoautosomal regions of the X chromosome in the absence of contamination), we used *ANGSD*^51^. We measured the cytosine-to-thymine mismatch rate to the consensus sequence at the terminal ends of sequences to determine if libraries had the expected damage profile for authentic aDNA. We also used the ratio of the Y chromosome to the X and Y chromosome to determine whether the individual had a ratio consistent with being from a person with two X chromosomes (molecular female: <0.03) or a person with an X and a Y chromosome (molecular male: >0.35); libraries with a ratio in between have potential evidence of contamination.

### Radiocarbon dating

We obtained 111 accelerator mass spectrometry-based dates on bone for 99 distinct human remains in specialized C14 laboratories at Pennsylvania State University (82 measurements), the D-REAMS laboratory at the Weizmann Institute of Science (24), Oxford (four), and CIRAM (one). For the CIRAM date, the different carbon isotopes were separated in a joint venture with JSC Barnas using a 250 kV accelerator mass spectrometer from FTMC in Vilnius, Lithuania. We report lab codes together with raw and calibrated measurements, including conventional radiocarbon ages and their standard errors (Supplementary Table 4). We used the calibration curve *IntCal20*^52^ to calibrate conventional radiocarbon dates using the software OxCal. Eleven individuals were analyzed by two different labs, with date ranges from the two labs being concordant in all cases. We used the R_combine method to combine date ranges into a single and more precisely constrained range for these individuals.

### Merging with published aDNA data

We merged the newly generated aDNA data with a collection of previously published ancient genomes obtained using version 54.1 of the *Allen Ancient DNA Resource*^53^. We generated a primary dataset of pseudo-haploid genotype data, with one allele picked randomly from sequences overlapping that position at the targeted SNPs (“1240k”). We used the resulting data in Eigenstrat format as the basis for our population genetic analysis.

### Data set partitions of ancient individuals

We categorized ancient individuals in the aDNA data set based on sequence coverage and the certainty of provenance. Our analysis in this study mainly focuses on 108 newly sequenced ancient individuals with more than 20,000 targeted autosomal SNPs covered by at least one sequence and its Phoenician or Punic association determined by the archeological context of a site and its radiocarbon dates. We applied a series of criteria to ensure our results did not include individuals associated with the Roman expansion following the Punic Wars (see Supplementary Information S2). We identified an additional set of 23 individuals with slightly reduced confidence in context: 10 individuals from Tharros, Birgi, Motya, and Lilybaeum that had radiocarbon date ranges that overlap the 3rd and 2nd centuries BCE, and 13 individuals from Tharros and sites in Iberia that did not have radiocarbon dates. We typically analyzed them separately because some of these individuals may post-date the Roman expansion after the Punic Wars. We added nine previously published individuals to these two groups, of which eight had radiocarbon dates^20,21,35^).

We grouped our sample into five broad geographic regions: Iberia (including the Iberian Peninsula and the island of Ibiza), Sardinia, Sicily, North Africa, and Akhziv. We also grouped our sample into two primary time ranges. The radiocarbon calibration curve for the first millennium BCE has several plateaus spanning hundreds of years, particularly the “Hallstatt plateau” 800–400 BCE and a subsequent plateau 400–200 BCE. Consequently, radiocarbon dating cannot resolve sample dates within each plateau. We thus associate each individual with one of these plateau time ranges (see Supplementary Information S2).

### Principal component analysis (PCA)

We computed Principal Components of modern individuals genotyped on the Affymetrix Human Origin SNP array using the software *smartpca* (version 18150) using default settings, combining data from a standard set of West Eurasians (HO data, widely used in human aDNA analysis) with present-day North-African individuals in AADR v54.1 (see list of 1196 individuals in Supplementary Table 6) as in^54^. We then projected onto the first two principal components ancient individuals with data for at least 20,000 SNPs on the autosomal targets, using least-square projection (setting *lsqproject: YES*) and shrinkage correction (setting *shrinkmode: YES*) and aDNA data in pseudo-haploid *eigenstrat* format.

### Ancestry modeling with ADMIXTURE

We ran *ADMIXTURE* version 1.3.0^55^ in unsupervised mode on the 122 individuals from our Phoenician-Punic data set that were sequenced for more than 100,000 SNPs, together with 24 individuals from related populations around the Mediterranean (Supplementary Table 12). Following^56^, we pruned SNPs in linkage disequilibrium by removing one SNP from every pair of SNPs that (1) were within a genomic window that contained at most 200 SNPs and (2) had an *r*^2^ value of association greater than 0.4. This pruning was done by applying *PLINK* V1.9^57^ with options --indep-pairwise 200 25 0.4. This process kept 452,215 SNPs out of the original 1,233,013. We applied *ADMIXTURE* to the pruned data set with *K* = 2, 3, 4 or 5 latent ancestry components, and for each value of *K*, we executed 50 replicate analyses with different random seeds. We used the Δ*K* score of^58^, as implemented in Clumpak^59^ to find the value of *K* that provided the best fit of the data and used *PONG* V1.5^60^ to visualize the results.

### General setup for qpAdm analysis

We estimated proportions of ancestry with *qpAdm*^27,28^, using the R package *admixr*^61^ and qpAdm version 1201 (from *ADMIXTOOLS*) with the *allsnps* flag turned on. We modeled background ancestry using 23 ancient individuals partitioned into 14 groups (Supplementary Table 7): El Miron, Ust Ishim, Kostenki, GoyetQ116–1, Vestonice, MA1, Villabruna, CHG (2 individuals), EHG (3 individuals), WHG (2 individuals), Mota, Natufian, Levant_N (4 individuals), and Tunisia_N (3 individuals). These 14 groups were used as “right populations” in all admixture models we considered, with El Miron always used as the first (anchor) right population. Considering the main clusters observed in the PCA, we determined eight groups of ancient individuals to use as proxies for potential sources of ancestry (Supplementary Table 7): Greece BA (Myc), Sicily EMBA, Sardinia LBA, Iberia EBA, Steppe MLBA, Iran N, Levant MLBA, and North Africa IA. As a proxy for North African ancestry, we use a single individual from Iron Age Algeria sequenced to high coverage (>750,000 SNPs). Since three Neolithic individuals from Tunisia are used to model background ancestry, North African ancestry inferred by our qpAdm models should reflect an African source population more closely related to the Iron Age individual from Algeria than to the Neolithic individuals from Tunisia (see Supplementary Information S3). Each of the other seven groups contains 6–24 individuals from a similar archaeological context, which were sequenced with high coverage (>100,000 SNPs) and form tight clusters in the 2D PCA (Figure 1). In each admixture model we examined, some of these eight groups were used as proxies to source populations (“left populations”), others were added to the model of background ancestry (“right populations”), and the remaining populations were left out of the analysis. An admixture model was considered *feasible* if all proxy sources in that model were associated with non-negative admixture proportions. A feasible model was considered *valid* if the P-value provided by the statistical test of qpAdm was at least 0.05 (indicating that this model cannot be rejected with high probability).

### Types of qpAdm ancestry models

In preliminary analysis with *qpAdm*, we considered broad ancestry models, which used only the basic set of 14 groups as right populations (see above). We considered all 255 non-empty subsets of the eight potential proxy sources as left populations. In these broad ancestry models we observed that each individual was fit by many valid admixture models with somewhat conflicting interpretations (see Supplementary Information S3). We thus decided to consider two separate and distinct types of admixture models. In the western ancestry models, we added the Levant MLBA group to the core set of 14 right populations. We considered the 127 non-empty subsets of the seven remaining proxy sources as left populations. In the eastern ancestry models, we considered the 7 non-empty subsets of Levant MLBA, Iran N, and North Africa IA as left populations, and the remaining five (western) proxy sources were added to the core set of 14 right populations. This approach produced interpretable valid admixture models for 135 of the 140 individuals in our Phoenician-Punic data set. To complete the picture, we provide admixture models inferred by the broad ancestry model described above to four of the remaining five individuals. This left only one individual (I22122 from Tharros) for which we could not fit a valid admixture model.

### Parsimonious ancestry models

The qpAdm analysis considered 134 different models for each individual (127 western models and 7 eastern models; see details above). As a result, many individuals obtained multiple valid ancestry models (models with non-negative ancestry proportions and P-values above 0.05). We applied the following parsimonious approach to reduce the number of valid models we report for each individual. For a given model M, let *pop*(M) denote the set of left populations used in this model. Now, consider two models, M and M’, that differ by one proxy source: *pop*(M) = *pop*(M’) U {p}. If both models are valid for a given individual, and the fit provided by model M is not significantly better than that provided by the more restrictive model M’, then we discard model M from the set of models we report for that individual. Formally, model M is considered to be *non-parsimonious* for a given individual if there is a model M’ that satisfies the following three requirements: (1) *pop*(M’) U {p} = *pop*(M); (2) model M’ is valid for the individual of interest; and (3) the P-value of M is not significantly larger than the P-value of M’. The difference between P-values is evaluated using a standard likelihood ratio test between the two nested models. Using this approach, we removed non-parsimonious models from the set of ancestry models we report for each individual (Extended Data Figure 3). Ancestry contributions in the parsimonious models we report should thus be considered significant in the sense that a model without them was rejected by qpAdm.

### Representative ancestry models

We applied several simplifications when presenting a representative admixture model for individuals in Figure 2. First, we combined the ancestry proportions inferred for Greece BA (Myc) and Sicily EMBA (Sicilian-Aegean ancestry), and the ancestry proportions inferred for Sardinia LBA and Iberia EBA (western Mediterranean ancestry). These ancestries were grouped due to the limited ability of our qpAdm models to distinguish between them (Supplementary Information S3) and their genetic similarity (Supplementary Information S6). Since individuals were typically inferred to have more than one valid admixture model, we selected a representative model for each individual that maximized the main source of ancestry. In particular, among eastern ancestry models, we selected the model with the largest proportion of Levantine ancestry. Among western ancestry models, we selected the model with the largest proportion of Sicilian-Aegean ancestry. For the six individuals for which we could fit both eastern and western models, we selected the model for which this main contribution (Sicilian-Aegean or Levantine) was the largest. Thus, the selected representative model reflects the maximum amount of ancestry attributed to the main source of ancestry.

### Inferring uniparental haplogroups

We used automatic Y haplogroup calling using a script described in^38^ based on *YFull YTree v. 8.09* phylogeny and SNPs from ISOGG *YBrowse6*. For cases with low coverage or unusually shallow assignments, we double-checked the assignments by inspecting all derived SNPs - assigning haplogroups in cases of clear “paths” to the most derived haplogroups - allowing for mismatches due to sporadic aDNA damage (i.e., C->T or G->A). We annotate all haplogroups in ISOGG19 annotation. We determined mitochondrial haplogroups using Haplogrep v2.1.1^50^ for sequences aligning to the mitochondrial genome (RSRS), utilizing all sequence data aligning to the mitochondrial genome RSRS.

### Calculating autosomal and Y haplogroup diversity per site

We calculated a proxy for the autosomal diversity at each site based on the first two PCs of our primary PCA (as described above) based on the rationale that those two PCs broadly reflect a genetic map of the Mediterranean. For each site with at least ten individuals with at least 20,000 1240k SNPs covered, we calculated the pairwise Euclidean distance of all pairs of individuals and then took the mean value as a measure for the per-site diversity.

To quantify Y haplogroup diversity per archaeological site, we considered the first three characters of the ISOGG classification for each inferred haplogroup. We used this particular cutoff for two reasons: (1) low coverage aDNA does not consistently allow assigning Y haplogroups at more refined levels due to missing data and genotyping errors caused by aDNA damage; (2) the specific 1240k SNP set enriched in these genome sequences do have little resolution for several macro-haplogroups beyond this level (e.g., I1). We calculated the inverse of the probability that two randomly selected males from a given site have the same Y haplogroups. This measure is known as the *Inverse Simpson index* or the effective number of types (i.e., the number of equally abundant types needed to obtain the same diversity index). We require at least five males for each site, using 100,000 autosomal SNPs covered as a cutoff for sufficient genomic data for Y haplogroup calls.

For both diversity measures, in Punic sites we included individuals with a date consistent with the Punic period (labeled *Punic_Early, Punic_Late, Punic_Late2, Punic_NoRC* in the column *labelPCA* in Supplementary Table 5). Including individuals dating to 350–50 calBCE and individuals without radiocarbon dates was necessary to obtain statistically meaningful sample sizes for quantifying diversity.

### Detecting shared haplotypes (IBD and ROH segments)

To infer pairwise shared identity by descent segments (IBD), we applied the software *ancIBD*^62^ designed for aDNA data. We started from processed .bam files containing all aligned reads after quality control. We then imputed genotype probabilities with the software *GLIMPSE*, using the same imputation pipeline as described in^62^ based on the 1000 Genome reference panel. Using the recommended default settings of *ancIBD*, we inferred all IBD segments longer than eight centimorgan (cM) in all pairs of individuals with the recommended quality cutoff: at least 70% of imputed SNPs on Chromosome 3 imputed with maximum genotype probability>0.99, which corresponds broadly to at least 600,000 SNPs covered for 1240k SNP capture data (Supplementary Table 9). We report summary statistics of IBD segments per pair of individuals in Supplementary Table 8. To infer degrees of relatedness, we compared inferred IBD segments >12cM long to IBD observed in simulated genomes with various degrees of relatedness when using the same filtering as when running *ancIBD*, as described in^62^. We considered a pair of individuals to be significantly related if they shared an IBD segment of length >16 cM. Two individuals with a shared segment 16 cM long have a common ancestor within 20 generations with high probability. Indeed, assuming a panmictic population of diploid individuals with constant size *N*_e_ = 1,000, 95% of such IBD segments originate from a common ancestor within up to 19.3 generation ago, with a median of 8.0 generations ago, calculated as in Note S4 of^30^. Varying *N*_e_ only has a minor effect on these numbers, and longer IBD segments originate from even more recent ancestors.

We inferred runs of homozygosity (ROH) using the software *hapROH* designed and calibrated for aDNA data^30^. We applied *hapROH* with its recommended default settings to pseudo-haploid data on 1240k SNPs using the modern 1000 Genomes haplotype panel as a reference panel. We used the recommended cutoff of at least 400,000 covered 1240k SNPs as a minimum coverage cutoff and inferred ROH >4 cM long. We report summary statistics of ROH segments per individual in Supplementary Table 10.

### Inferring pairwise genetic relatedness

We computed pairwise mismatch rates by randomly sampling one read for each individual at the autosomal SNPs of the 1240k panel and computed 95% confidence intervals using block jackknife standard errors (Supplementary Table 11). For individuals in T774 from Villaricos, we then estimated kinship coefficients r as described in^63^: r = 1 – (2*(x-(b/2))/b), where x denotes the pairwise mismatch rate for that pair and b the mismatch rate expected for two unrelated individuals from the same ancestral background. We estimated b as the mean of the mismatch rates between individuals from T774 and Bronze-Iron Age individuals with similar ancestry from Empúries^54^ and mainland Greece^38,54^, who are very unlikely to be close relatives to those from T774.

## Extended Data

**Extended Data Figure 10:**
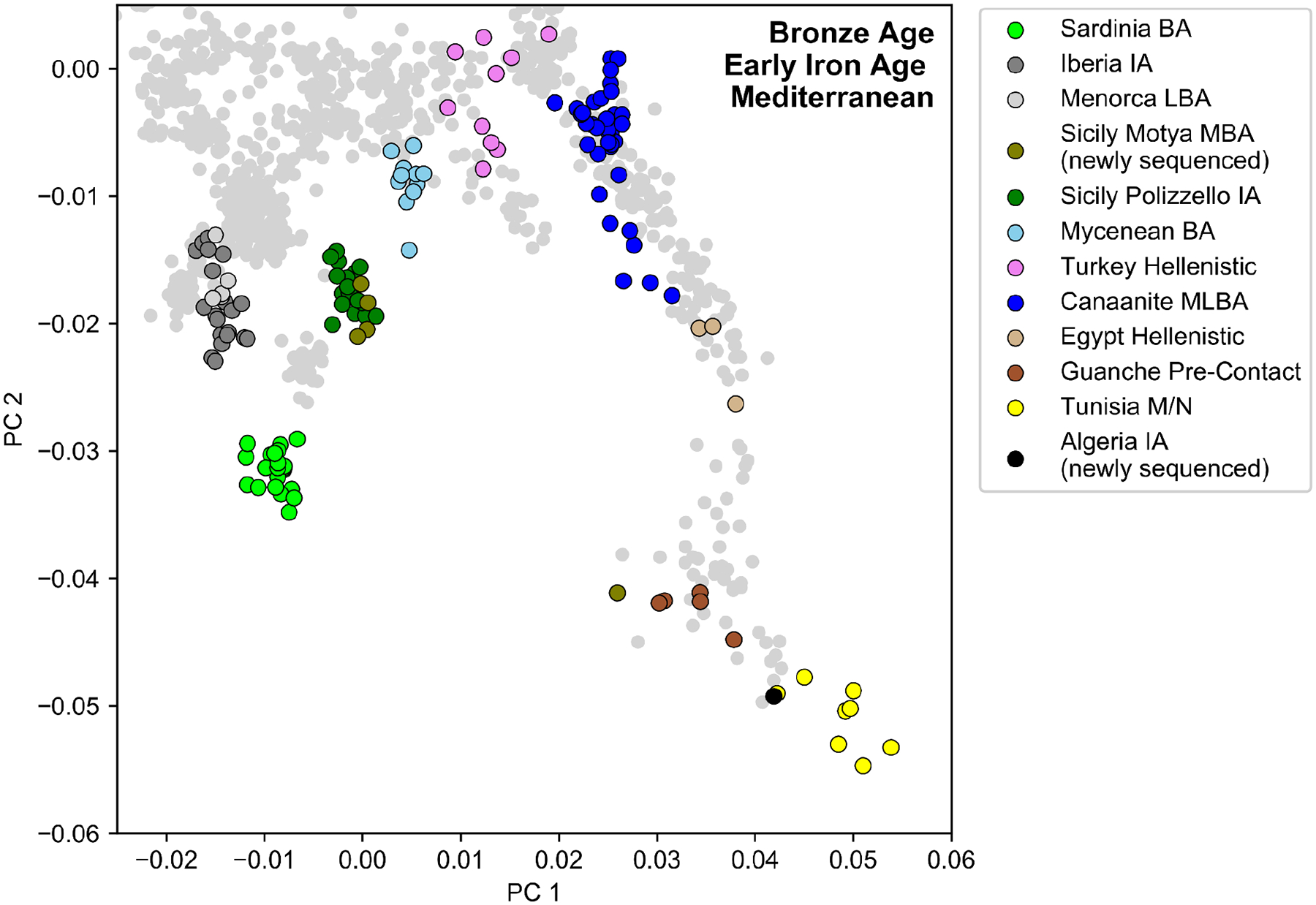
Additional PCA projections. **(a) Bronze and Iron Age reference and Levantine populations.** We show the same PCA as in Figure 1 but focus on the ancient reference populations. (b) Zoom in PCA projections of Levantine populations. We show the same PCA depicted in (a), but zooming into the region where Levantine individuals project. We also include additional Bronze and Iron Age Levant individuals not included in Figure 1. Those previously published individuals originate from Sidon in present-day Lebanon^17^ and various sites in present-day Israel (Megiddo, Yehud, Hazor, Baq’ah^25^, Tel Shadud^64^, Ashkelon^64,65^). Abbreviations: MLBA: Middle-Late Bronze Age, MBA: Middle Bronze Age, IA: Iron Age. All 13 individuals from Akhziv cluster next to other Levantine individuals, together with a single outlier individual from Tharros (I22119) inferred to have Levantine ancestry (Extended Data Figure 3). Abbreviations: M/N: Mesolithic/Neolithic, MLBA: Middle-Late Bronze Age, MBA: Middle Bronze Age, LBA: Late Bronze Age, IA: Iron Age

## Supplementary Material

supplementary information

Supplementary Tables

## Data Availability

The raw DNA sequences for individuals newly sequenced in this study are deposited in the European Nucleotide Archive under accession number PRJEB86313. Their processed genotype data in pseudo-haploid eigenstrat format can be obtained from the Harvard Dataverse repository at https://doi.org/10.7910/DVN/UPDESR. We include other newly reported data such as radiocarbon dates and archaeological context information in the manuscript and supplementary files. We plotted the maps in Figure 1 and Figure 4 using the Python package *basemap*. The land-sea mask, coastline, lake, river, and political boundary data are extracted from the GSHHG datasets (version 2.3.6) using GMT (5.x series). They are included under the terms of the open-source LGPLv3+ license.
