# Supplementary material for: Punic people were genetically diverse with almost no Levantine ancestors
Source: Nature. Author manuscript; Available in PMC 2025 Jul 4. (PMC12226237; doi:10.1038/s41586-025-08913-3)
Supplement: supplementary information [file NIHMS2078714-supplement-supplementary_information.pdf]

Supplementary Information

Punic people were genetically diverse with almost no Levantine ancestors

Harald Ringbauer[1,2,3], Ayelet Salman-Minkov[4,46], Dalit Regev[5], Iñigo Olalde[1,6,45], Tomer Peled[4], Luca Sineo[7], Gioacchino Falsone[8], Peter van Dommelen[9], Alissa Mittnik[1,2,3], Iosif Lazaridis[1,14,15,16], Davide Pettener[10], Maria Bofill[11], Ana Mezquida[11], Benjamí Costa[11], Helena Jiménez[11], Patricia Smith[12], Stefania Vai[13], Alessandra Modi[13], Arie Shaus[1,47,48], Kim Callan[14,16], Elizabeth Curtis[14,16], Aisling Kearns[14], Ann Marie Lawson[14,16], Matthew Mah[14,15,16], Adam Micco[14], Jonas Oppenheimer[14,16], Liju Qiu[14,16], Kristin Stewardson[14,16], J. Noah Workman[14], Nicholas Márquez-Grant[17], Antonio M. Sáez Romero[18], María Luisa Lavado Florido[18], Juan Manuel Jimenez-Arenas[19], Isidro Jorge Toro Moyano[20], Enrique Viguera[21], Jose Suarez Padilla[21], Sonia López Chamizo[21], Tomas Marques-Bonet[22], Esther Lizano[22], Alicia Rodero Riaza[23], Francesca Olivieri[24], Pamela Toti[25], Valentina Giuliana[26], Alon Barash[27], Liran Carmel[28], Elisabetta Boaretto[29], Marina Faerman[30], Michaela Lucci[31], Francesco La Pastina[31,35,36], Alessia Nava[32], Francesco Genchi[33], Carla Del Vais[34], Gabriele Lauria[35], Francesca Meli[36], Paola Sconzo[36], Giulio Catalano[37], Elisabetta Cilli[38], Anna Chiara Fariselli[38], Francesco Fontani[2,3,39], Donata Luiselli[38], Brendan J. Culleton[39], Swapan Mallick[14,15,16], Nadin Rohland[1,14,15], Lorenzo Nigro[41], Alfredo Coppa[32,40,42]*, David Caramelli[13]*, Ron Pinhasi[42]*, Carles Lalueza-Fox[43, 44]*, Ilan Gronau[4]*, David Reich[1,2,14,15,16]*

Correspondence to:
harald_ringbauer@eva.mpg.de, ilan.gronau@runi.ac.il, reich@genetics.med.harvard.edu
* Co-supervised this work

[1]Department of Human Evolutionary Biology, Harvard University, Cambridge, MA, USA

[2]Max Planck Harvard Research Center for the Archaeoscience of the Ancient Mediterranean (MHAAM), Leipzig, Germany

[3]Department of Archaeogenetics, Max Planck Institute for Evolutionary Anthropology, Leipzig, Germany

[4]Efi Arazi School of Computer Science, Reichman University, Herzliya, Israel

[5]Israel Antiquities Authority, Jerusalem, Israel

[6]BIOMICs Research Group, Department of Zoology and Animal Cell Biology, University of the Basque Country UPV/EHU, Vitoria-Gasteiz, Spain

[7]Dept. STEBICEF, Laboratory of Anthropology, University of Palermo, Palermo, Italy

[8]Dept. Culture e Società, University of Palermo, Palermo, Italy

[9]Brown University, Providence, RI, USA

[10]University of Bologna, Bologna, Italy

[11]Archaeological Museum of Ibiza and Formentera, Eivissa, Spain

[12]Faculties of Medicine and Dental Medicine, The Hebrew University of Jerusalem, Jerusalem, Israel

[13]University of Florence, Florence, Italy

[14]Department of Genetics, Harvard Medical School, Boston, MA, USA

[15]Broad Institute of MIT and Harvard, Cambridge, MA, USA

[16]Howard Hughes Medical Institute, Harvard Medical School, Boston, MA, USA
[17]Cranfield Forensic Institute, Cranfield University, UK
[18]Universidad de Sevilla, Seville, Spain
[19]Universidad de Granada, Granada, Spain
[20]Museo Arqueológico de Granada, Granada, Spain
[21]Universidad de Málaga, Málaga, Spain
[22]Unidad de Paleobiología, ICP-CERCA, Unidad Asociada al CSIC por el IBE UPF-CSIC, Cerdanyola del Vallès, Barcelona, Spain.
[23]Museo Arqueológico Nacional Madrid, Madrid, Spain
[24]Ministry of cultural heritage, Sicily region, Palermo, Italy
[25]The Giuseppe Whitaker Foundation, Motya, Italy
[26]University of Palermo, Department of Archaeology, Palermo, Italy
[27]Bar Ilan University, The Azrieli Faculty of Medicine, Safed, Israel
[28]Department of Genetics, The Alexander Silberman Institute of Life Sciences, The Hebrew University of Jerusalem, Jerusalem, Israel
[29]Weizmann Institute of Science, Scientific Archaeology Unit, D-REAMS Radiocarbon Dating Laboratory, Rehovot, Israel
[30]The National Natural History Collections, The Hebrew University of Jerusalem, Jerusalem, Israel
[31]Department of Environmental Biology, Sapienza University of Rome, Rome, Italy
[32]Department of Odontostomatological and Maxillofacial Sciences, Sapienza University of Rome, Rome, Italy
[33]Italian Institute of Oriental Studies, Sapienza University of Rome, Rome, Italy
[34]Università degli Studi di Cagliari, Cagliari, Italy
[35]Dept. STEBICEF, Laboratory of Anthropology, University of Palermo, Palermo, Italy
[36]Dept. Culture e Società, University of Palermo, Palermo, Italy
[37]Dept. STEBICEF, Laboratory of Anthropology, University of Palermo, Palermo, Italy
[38]Department of Cultural Heritage, University of Bologna, Ravenna, Italy
[39]Institute of Energy and the Environment, Penn State University, Pennsylvania, USA
[40]Department of Environmental Biology, Sapienza University of Rome, Rome, Italy
[41]Department of Ancient World Studies, Sapienza University of Rome, Italy
[42]Department of Evolutionary Anthropology, University of Vienna, Vienna, Austria
[43]Natural Sciences Museum of Barcelona, Barcelona, Spain
[44]Institute of Evolutionary Biology (UPF-CSIC), PRBB, Barcelona, Spain
[45]Ikerbasque—Basque Foundation of Science, Bilbao, Spain
[46]Department of Evolution and Ecology, University of California, Davis, CA, USA
[47]Department of Archaeology and Ancient Near Eastern Civilizations, Tel Aviv University, Tel Aviv, Israel
[48]Department of Data Science, Mount Holyoke College, South Hadley, MA, USA

Table of Contents

Section 1 - Archaeological site descriptions 4
Villaricos (Almería, Spain) 4
Puig des Molins (Eivissa, Spain) 5
Ses Païsses de Cala d'Hort (Eivissa, Spain) 7
Can Marines (Eivissa, Spain) 8
Solar Los Chinchorros (Cádiz, Spain) 8
San Fernando, Torre Alta (Cádiz, Spain) 10
Campo de Hockey (Cádiz, Spain) 11
Necropolis of Puente de Noy (Almuñécar, Granada, Spain) 12
Hipogeo del Parking de Alcazabilla (Málaga, Spain) 13
Calle Mármoles (Málaga, Spain) 14
Tharros (Sardinia, Italy) 15
Akhziv (Israel) 17
Motya (Sicily, Italy) 19
Motya (Sicily, Italy) University of Palermo excavations in the archaic necropolis 21
Motya (Sicily, Italy) “Area K” 23
Human remains from Motya city walls (Tower 1, Tower 4) 23
Birgi (Sicily, Italy) 25
Lilybaeum, Corso Gramsci necropolis (Sicily, Italy) 26
Lilybaeum, the Tribunale and Monumentale Necropolis (Sicily, Italy) 27
Lilybaeum - Human remains from the sectors of Punic Necropolis: Via Berta, Via De Gasperi, via Cicerone, Via D'Azeglio 29
Selinunte (Sicily, Italy) 30
Caserma Tukory, Palermo (Sicily, Italy) 31
Palermo. Human remains from the area of the “Istituto Maria Adelaide”, Palermo 33
Carthage (Tunis, Tunisia) 34
Kerkouane (Cap Bon, Tunisia) 45
Khenkela Cave (Constantine, Algeria) 47
Section 2 - Dataset description 49
Section 3 - Modeling ancestry and admixture using ADMIXTURE and qpADM 53
Section 4 - Prevalence of J2a and J2b Y chromosome haplogroups 61
Section 5 - Y Haplogroup and Autosomal Diversity in the Ancient Mediterranean 64
Section 6 - Genetic differentiation (FST) between different groups of Mediterranean individuals. 69
Supplementary References for Sections 2-6 71

Section 1 - Archaeological site descriptions

This section includes summaries of archaeological contexts sampled for this study. For every site described, we provide the following details:

- Authors who contributed to the site descriptions
- General Location and Chronology
- Excavation history
- Description of cemeteries
- References

Villaricos (Almería, Spain)

Carles Lalueza-Fox and Alicia Rodero Riaza

General Location and Chronology: The so-called archaeological area of Villaricos includes a complex of settlement, burial, and working areas dating from the Phoenician-Punic to the Arab-Andalusian times. The burial areas, as a whole, are traditionally referred to as the Villaricos necropolis, although they actually comprehend several cemeteries, burial plots, or even isolated tombs covering the mentioned chronological arch and corresponding to different settlements. Although the basic structure of the necropolis is a rectangular grave excavated in the terrain -but also amphora inhumations of infantile and cremations-, Villaricos is famously known for the large, excavated hypogea. Almost all of the hypogea had been looted and re-used in antiquity; however, some of them preserved original funerary materials as well as human remains. They are usually excavated into the solid rock, at least partially, and they have a funerary chamber accessed through a descending corridor -with or without stairs-. The surface area of these chambers ranges between 10 and 26 $m^{2,}$ and the access was closed by wooden structures, sometimes reinforced by large slab stones. The inside of the hypogea was parcelled with niches, walls, and sometimes with graves on the floor. The initial burials seem to have been made in wooden coffins, but subsequent re-uses have extended to other rituals. The original plan seems to correspond to the 6th century BCE, with persistent intensive use in the 5th and 4th centuries BCE. It continued to be used, at least partially, during Roman and Late Antiquity times.

Excavation history: Villaricos has a large funerary complex excavated at the end of the 19th century and the beginning of the 20th century by pioneer archaeologist Luis Siret (there are entries in his diaries from 1890 to 1914). The works were continued by Miriam Astruc and later by María José Almagro. Some results were published in 1906 by the *Real Academia de la Historia*, and several books were published detailing the findings (e.g., Astruc 1951). With the possible exception of Puig des Molins in Eivissa, Villaricos, with more than 1,842 documented tombs, constitutes the largest and richest Iberian necropolis from the Punic period. The materials from Siret's excavations were deposited in 1935 in the Museo Arqueológico Nacional (MAN) in Madrid, where they are still held. From the original 9,460 pieces listed by Siret, 5,781 - 370 of them corresponding to skeletal elements- have been located at the MAN (Rodero et al. 1996). Notably, this aDNA study demonstrates the value of such old archaeological collections, in this case almost a hundred years old, for genetic studies.

Description of cemeteries: Four tombs that yielded positive genetic results (T414, T693, T774, and T937) correspond to rock-carved hypogea (Astruc 1951); these are roughly rectangular chambers with rather irregular sides, sometimes with some stonework to hide flaws in the rock and with niches along the walls. T414 included cremation remains in stone recipients and one knife shaft made of bone. In T693, several small ceramic cups, a censer, fragments of a ceramic human figurine, one thin ring of gold, and two gold pendants were found; also, the remains of a falcata sword, a spear, and the metal part of a shield were uncovered in this hypogeum. In tomb T774, two black-glazed vessels of Campanian tradition, small unguent jars, and remains of spears were discovered. Perhaps the most interesting finding was within T774: Two small ivory plaques, one engraved with an Ionic capital and the other one with a fragmentary Greek theme; both plaques were incised to be fixed on a surface (Astruc 1951). In tomb T937, a circular bronze mirror, and some bronze coins were excavated. In the four hypogea described, remains of ostrich shells -an element of social prestige abundantly found across the Villaricos tombs- were uncovered.

Astruc (1951) describes one more tomb with genetic results (T62) as an inhumation in a large grave. Considering the localization and the radiocarbon date, it should be part of a cemetery belonging to the Punic settlement ("población púnica").

References

Almagro Gorbea. Mª J. (1984): *La necrópolis de Baria (Almería.). Campañas de 1975-78.* Excavaciones Arqueológicas en España 129. Madrid, Ministerio de Cultura.

Astruc, M. (1951). La necrópolis de Villaricos. Informes y Memorias, 28. C.G.E.A. Ministerio de Educacion Nacional. Madrid.

Pereira, J, Rodero Riaza, A, Chapa Brunet MT, Perea Caveda A, Madrigal Belinchón A, Pérez Diez MdC (1996). La necrópolis de Villaricos (Almería). Complutum Extra 6(I): 373-383.

Siret, L. (1906): *Villaricos y Herrerías. Antigüedades púnicas, romanas, visigóticas y árabes*. Madrid, Real Academia de la Historia.

Puig des Molins (Eivissa, Spain)

Carles Lalueza-Fox, Ana Mezquida, Helena Jiménez and Nicholas Márquez-Grant

General Location and chronology: Eivissa is an island of about 570 km^2 located 92 km from the Iberian Peninsula's coast, 82 km from Majorca island and 240 km from the North African coast. Puig des Molins is a large necropolis located on the southern slopes of the present city of Eivissa, at 51 meters above sea level and 500 meters from the old Punic town. It started to be used in the 7th century BCE with Phoenician cremations and ended with Islamic burials in the 13th century CE (Costa y Fernandez 2003). From the 6th century BCE onward, Carthage took political and economic control of the Western and Central Mediterranean and centralized their economic system in a set on some key commercial enclaves such as Cádiz, Málaga, and Eivissa (Aubet 1995). The establishment of these settlements involved a demographic increase, more extensive agricultural exploitation,

changes in the funerary rituals and religious practices, and also unprecedented production of commercial products. In this context, Eivissa was an important urban center integrated into a commercial network, mainly with Sardinia and Sicily (Costa y Fernandez 2000).

It was during the 5th and 4th century BCE when the necropolis experienced a period of maximum usage that can be summarized into the following phases:

a) Early Punic stage (ca. 530/525 to 450 BCE): The predominant burial rite is inhumation, although some cremations are also present. Rock-carved graves are the most predominant tomb type.

b) Middle Punic stage (ca. 450-200 BCE): There is a great expansion of the cemetery with an estimated 6,000 to 7,000 hypogea. A decrease in the number of tombs is apparent between 350 and 200 BCE, with about 10% of them being reused.

c) Late Punic stage (ca 200-25 BCE): There is a return to the cremation rites, coexisting alongside inhumations.

Excavation history: Puig des Molins has been extensively studied since the beginning of the 20th century. A significant part of the necropolis is protected today (5 ha). However, some sections are currently covered by modern city buildings, and some emerge during urban development constructions (Gómez Bellard et al. 1991; Costa & Fernández 2003a, 2003b). Most of the materials are currently preserved at the Museu Arqueològic d'Eivissa i Formentera (MAEF), located in the city of Eivissa. The necropolis was declared a UNESCO World Heritage Site in 1999 as one of the Western Mediterranean's largest and best-preserved Phoenician and Punic necropolis.

Description of cemeteries: The most abundant and characteristic of Puig des Molins is a large number of rock-carved Punic hypogea. It is estimated that there are around 3.000-4.000 such hypogea. Other types of tombs have also been documented: natural cavities, cremation burials in the ground or inside urns, rectangular pits with lateral steps, simple pits, and child burials in amphorae, among others (Costa & Fernández 2003b). Various rich grave goods have been found in the Punic tombs, including Greek pottery, jewelry, amulets, scarabs, decorated ostrich eggs, or terracottas (Mezquida 2022).

References

Costa, B., Fernández, J. H. (2003a): “El Puig des Molins, de campos de cultivo a Patrimonio de la Humanidad: Un siglo de Historia (1903-2003)”, *Misceláneas de Arqueología Ebusitana II. El Puig des Molins (Eivissa): Un siglo de Investigaciones*. Treballs del Museu Arqueològic d’Eivissa i Formentera, n.º 52, pp. 23-86.

Costa, B., Fernández, J. H. (2003b): “Necrópolis del Puig des Molins (Eivissa): las fases fenicio-púnicas”, *Misceláneas de arqueología ebusitana (II): El Puig des Molins (Eivissa): Un siglo de investigaciones*, Trabajos del Museo Arqueológico de Ibiza, 52, Ibiza, pp. 87-148.

Gómez Bellard, C., Costa, B., Gómez Bellard, F.. Gurrea, R.; Grau, E., Martínez, R. (1991): *La colonización fenicia en la isla de Ibiza*. Ministerio de Cultura. Madrid.

Mezquida, A. (2022): *Ritual funerario en la necrópolis del Puig des Molins (Ibiza): La excavación de 2006*. Monografies del Museu Arqueològic d'Eivissa i Formentera, 1. Ibiza.

Ses Païsses de Cala d'Hort (Eivissa, Spain)

Carles Lalueza-Fox, Maria Bofill and Nicholas Márquez-Grant

General Location: This Punic rural necropolis, also known as Can Sorà, is located in the southwestern part of the island of Eivissa. The cemetery is part of a larger archaeological complex, including two buildings used since pre-Roman times, two Byzantine tombs, and later additions such as the more recent farmsteads. This Punic necropolis, probably the best-preserved Punic rural cemetery on the island, was used as a burial place between 500-400 BCE and 100-75 BCE (Ramon, 1995).

Excavation history: Eighteen rock-carved tombs (hypogea) were discovered by 1917 by an excavation undertaken by Carlos Román Ferrer, and some additional ones (disturbed by the previous excavation) were found in subsequent excavations in the 1980s and 1990s by Dr. Joan Ramon Torres (Ramon, 1995).

Description of cemeteries: The human remains were recovered during J. Ramon Torres' excavations (Ramon, 1995) were found commingled within a number of hypogea. The hypogea varied in size, but due to subsequent changes in the terrain, many of the roofs collapsed. The anthropological analysis has been published by Márquez-Grant et al. (2021) and previous isotope analyses, including provenance as well as diet in Nechlich et al. (2012) and Fuller et al. (2010), respectively.

References

Fuller, B., Márquez-Grant, N. and Richards, M.P. (2010). Investigation of diachronic dietary patterns on the islands of Ibiza and Formentera, Spain: evidence from carbon and nitrogen stable isotope ratio analysis. *American Journal of Physical Anthropology,* 143: 512-522.

Márquez-Grant, N., Olivé Busom, J. and Ramon Torres, J. (2021). Los restos humanos de la necrópolis púnica de Ses Païsses de Cala d'Hort (Can Sorà, Ibiza): Estudio antropológico. *Coloquio Fenicio-Púnico Noviembre 2019.* Museu Arqueològic d'Eivissa i Formentra, Ibiza. Pages: 383-421.

Nehlich, O., Fuller, B., Márquez-Grant, N. and Richards, M.P. (2012). Investigation of diachronic dietary patterns on the islands of Ibiza and Formentera, Spain: evidence from sulphur stable isotope ratio analysis. *American Journal of Physical Anthropology*, 149: 115-124.

Ramón, J. (1995). Ses Paisses de cala d'Hort. Un establiment rutal d'època antiga al sud oest d'Eivissa. Quaderns d'Arqueologia Pitiüsa 1 (Ibiza).

Can Marines (Eivissa, Spain)

Carles Lalueza-Fox, Benjamí Costa and Nicholas Márquez-Grant

General Location and Chronology: Can Marines is a Punic rural site located in Sant Carles parish, within the limits of Santa Eulària des Riu village in the northeastern part of the island of Eivissa. The funerary objects retrieved suggest that the necropolis was used between the end of the 5th century BCE and the 1st century BCE.

Excavation history: The excavations started with the accidental discovery of a hypogeum in agricultural exploitation in 1980. Some years later, a new excavation confirmed the existence of two hypogea. The site itself has not been archaeologically described, with the exception of some anthropological and isotopic literature (Gómez-Bellard 1989, Salazar-García 2011). The anthropological study of the remains determined the presence of at least 28 individuals.

Description of cemeteries: The sample described here derives from one hypogeum containing human remains. The other contained one stone sarcophagus, but no human remains. The excavators pointed out that more tombs and a rural domestic structure could be discovered in the future, as seen in other examples on the island, such as Ses Païsses de Cala d'Hort.

References

Gómez-Bellard, F. (1989). Antropología médica en Ibiza. Ed. Universidad Complutense de Madrid; Madrid.

Salazar-García, D.C. (2011). Patrón de dieta en la población púnica de Can Marines (Ibiza) a través del análisis de isótopos estables (C y N) en colágeno óseo. Sagvntvm 43: 95-102.

Solar Los Chinchorros (Cádiz, Spain)

Antonio M. Sáez Romero and María Luisa Lavado Florido

General Location and Chronology: The site is located in the modern urban expansion district of the city of Cádiz. During the 1st millennium BC, the area apparently was initially used by several burial areas and cremations dating back to the late Phoenician Archaic period (7th-6th centuries BC), which were abandoned and partially damaged by the construction of a building involved in the production of salted fish. The plan and number of vats of this facility are unclear, although its activity must have extended over subsequent phases from at least the beginning of the 5th century BC to the end of the 2nd or the beginning of the 1st century BC. The pits distributed around the settlement indicate an important activity developed between the 5th and 3rd centuries BC, with abundant transport of amphorae and other local and imported ceramics documented in these deposits. In the 2nd century BC, the building was extensively renovated and enlarged, perhaps to include four oval basins (three of which were preserved), several rooms paved with "opus signinum" and a kiln (whose probable connection with pottery production was not fully clarified). After the abandonment of this craft center, it seems that there must have been an evolution in the land planning in this sector again, leaving it as a peripheral area with few traces of use

during the Late Republican and Imperial periods (only some scattered structures, wells and basins, a possible clay quarry, and several tombs from the 1st-2nd centuries AD).

Excavation history: The salvage excavations carried out in 2007-2008 (directed by M.L. Lavado Florido) allowed the investigation of a large area located in the modern urban expansion district of the city of Cádiz. The two phases of excavation revealed evidence that can be dated between the end of Late Prehistoric times (evidence of sporadic occupations of the Chalcolithic-Bronze Age) and the medieval-modern period (part of the coastal road or "Arrecife").

Description of cemeteries: From the end of the 7th century and through the 6th century BCE, the area was probably used predominantly for funerary purposes. Several primary cremations dating from this period were documented both to the north and west of the Punic building, grouped together, resulting in two small groups of common tombs with very few grave goods. Both groups of burials, as well as a similar isolated one found to the south of the building (this one with interesting grave goods including jewelry, glass beads, and worked ivory), were partially damaged after the change of use of the area in the 5th-2nd centuries BCE due to the rise of artisanal activities (fishing, pottery, quarrying, etc.). Only a few vertebrae and long bones, with no anatomical connection, were found of the individual studied in this paper (the remains were mixed with ceramics and other items within the large pit).

The sample comes from the pit excavated in sector E1/F1. It is a large structure, partially excavated into the yellowish marls layer, where debris and many ceramics were accumulated mainly between the end of the 4th century and throughout the 3rd century BC. It is likely that the excavation of the pit, related to the nearby building identified as a fish salting facility, damaged or destroyed older graves (6th century BC) located in this northern part of the site. That is probably the reason why the human skeletal remains ended up buried in a later pit, which served an artisanal function, not a funerary one.

References

Lavado Florido, M.L. (2010): "Ajuar funerario de Los Chinchorros. Cádiz", in M. D. López & E. García (eds.) *Cádiz y Huelva. Puertos fenicios del Atlántico. Catálogo de la Exposición* (Museo de Cádiz-Museo de Huelva, 2010-2011), Cádiz, 314-315.

Sáez Romero A.M., Lavado Florido, M.L. (2016): “Calle San Bartolomé/Los Chinchorros (Cádiz, España)”. RAMPPA, Red de Excelencia Atlántico-Mediterránea del Patrimonio Pesquero de la Antigüedad [URL: http://ramppa.ddns.net/cetaria/calle-san-bartolome-los-chinchorros] [Actualizada el 23/11/2016].

Sáez Romero A.M., Lavado Florido, M.L.(2019): “Cremaciones fenicias y un nuevo saladero de pescado púnico de Gadir. Avance de los hallazgos registrados en el área de Los Chinchorros (calle San Bartolomé, Cádiz)”. Habis 50, pp. 49-81.

Sáez Romero A.M., Lavado Florido, M.L. (2021): “Cerámicas griegas en Gadir entre los siglos V-III a. C. Nuevos datos de las instalaciones conserveras púnicas de San Bartolomé (Cádiz)”, in Andrés Carretero, Marigel Castellano, Margarita Moreno Conde y Concha Papí

Rodes (eds.) ABANTOS. Homenaje a Paloma Cabrera Bonet. Madrid: Museo Arqueológico Nacional, 253-264.

Zamora, J.A., Sáez Romero A.M., Lavado Florido, M.L.(2021): “Estampillas anfóricas y grafitos recuperados en el solar de “Los Chinchorros” (Calle San Bartolomé, Cádiz)”, Revista Atlántica-Mediterránea de Prehistoria y Arqueología Social, 22: 139-168.

San Fernando, Torre Alta (Cádiz, Spain)

Antonio M. Sáez Romero

General Location and Chronology: The site is located in the southern part of the present-day island of Cadiz, in an area generally identified with the Antipolis mentioned by Strabo (3.5.3-5). Throughout the 1st millennium BC and in relation to the territory of the Phoenician city of Gadir, in this insular sector, numerous artisanal facilities were established, mainly linked to the production of pottery, salt, fishing, and fish processing. Several dozens of pottery workshops exploited the clay soils of the area at least between the late 6th century BC and the 1st century AD, sometimes almost uninterruptedly and sometimes with significant hiatuses, but usually in an organized landscape model that was shaped at the beginning of the Punic period and continued at least until the beginning of the Roman-Republican phase. Torre Alta was one of these settlements. It is currently one of the most excavated and studied facilities, allowing us to analyze the typology of such facilities from the 3rd to 2nd centuries BC (pottery production in the area began in the 5th century BC, but the later phase is the best preserved)**.**

Excavation history: The site was investigated through occasional salvage excavations between 1987 and 2003. These excavations allowed the excavation of the entire site and the investigation of the surroundings of the core area, detecting some possible clay quarries and other secondary structures. The main field seasons (1987-1988, 1995, 1997, and 2001-2003) revealed the existence of eight ceramic kilns and several pits with large amounts of debris and refused pieces, as well as thousands of ceramic sherds. These structures were part of an artisanal complex of relatively small dimensions, with no evidence of other buildings nearby (probably built with mud bricks and wood, such as storerooms, areas for the potter's wheel, etc.). All of this can be dated between the 3rd century and the first two-thirds of the 2nd century BCE that is, between the end of the Punic city and the beginning of the Roman period.

Description of cemeteries: During the 1995 excavation season, some bone remains were identified in a sector to the south of the ceramic kilns. These were presumably the remains of several graves, destroyed since ancient times, which seem to correspond to single pit burials (without a stone cover) of a few adults. Only some ceramic unguentaria were associated with them, dating the funerary area to around the mid-2nd century BC (Sáez & Díaz 2010: 272-275). It is possible that they were artisans linked to the workshop itself during the latter stages of production, although the poor preservation of the remains and the lack of a clear context have not allowed us to gather conclusive data in this regard.

References

Arteaga, O., Castañeda, V., Herrero, N., Pérez, M. (2001): "Los hornos tardopúnicos de Torre Alta (San Fernando, Cádiz). Excavación de urgencia de 1997", Anuario Arqueológico de Andalucía/1997, vol. III, Sevilla, 128-136.

De Frutos, G., Muñoz, A. (1994): "Hornos Púnicos de Torre Alta (San Fernando, Cádiz)", in J.M. Campos, F. Gómez & J.A. Pérez (eds.) Arqueología en el entorno del Bajo Guadiana, I Encuentro de Arqueología del Suroeste, Huelva-Niebla: Universidad de Huelva, 396-414.

Muñoz Vicente, A., De Frutos, G. (2006): "El complejo alfarero de Torre Alta en San Fernando (Cádiz). Campaña de excavaciones de 1988. Una aportación al estudio de la industria pesquera en la Bahía de Cádiz en época tardopúnica", in I Conferencia Internacional Historia de la Pesca en el ámbito del Estrecho (1-5 junio de 2004, El Puerto de Santa María), II, Sevilla, 705-803.

Sáez Romero, A. M. (2008): La producción cerámica en Gadir en época tardopúnica (siglos –III/-I). BAR International Series, 1812 (2 vols.), Oxford.

Sáez Romero, A.M., Díaz Rodríguez, J.J. (2010): "La otra necrópolis de Gadir/Gades. Enterramientos asociados a talleres alfareros en su hinterland insular", in A. M. Niveau & V. Gómez (Coords.) Las necrópolis de Cádiz. Apuntes de arqueología gaditana en homenaje a J. F. Sibón Olano. Servicio de Publicaciones de la Universidad de Cádiz. Cádiz, 251-337.

Campo de Hockey (Cádiz, Spain)

Antonio M. Sáez Romero

General Location and Chronology: Campo de Hockey is a prominent Neolithic necropolis, described in detail in Olalde et al. (2019). However, south of the Neolithic site's limit, a single, shallow inhumation was discovered together with undiagnostic ceramic materials from the Punic-Roman period. In the vicinity, some structures can be dated from the Punic to the Late Roman periods and could correspond, in fact, to places of pottery production.

Excavation history: Described in Olalde et al. (2019).

Description of cemeteries: The individual was buried in a supine decubitus position and West-East direction, but not flexed like all Neolithic individuals. Inside the burial, there were undiagnostic ceramic materials from the Punic-Roman period.

References

Olalde et al. (2019). The genomic history of the Iberian Peninsula over the past 8000 years. Science. 363. 1230-1234. 10.1126/science.aav4040.

Necropolis of Puente de Noy (Almuñécar, Granada, Spain)

Juan Manuel Jimenez and Isidro Jorge Toro Moyano

General Location and Chronology: The Necropolis of Puente de Noy (Almuñecar, Granada, Spain) is, besides the Laurita Necropolis, the most important Pheno-Punic necropolis of the ancient city of Sexi. This necropolis is located on the southern slope of a low elevation between the Peñón del Santo and the Punta de San José and is divided into 5 areas (A, B, B-C, D, and E) encompassing burials from century 7th BCE to century 1st CE (Molina-Fajardo & Huertas-Jiménez, 1985).

Description of cemetery: Tomb 11 is located in the named D Area. This sector is located in the southmost part of the necropolis. According to Molina-Fajardo and Huertas-Jiménez (1983, 1985), Tomb 11 is of type 2, featuring a rectangular fossa with vertical walls. The orientation of this tomb is NW-SE. Such disposition is very similar to that of the other 6 tombs very close to it (among them, Tomb 14). The remaining tombs are orientated N-S. Although Tomb 11 was looted (most of its original contents are missing, and the found pottery is scarce, fragmentary, and not very representative from a chrono-cultural point of view), it was dated to the first century BCE (Huertas-Jiménez & Molina-Fajardo, 1983; Molina-Fajardo & Huertas-Jiménez, 1985). For its part, Tomb 14 is located in the same area as Tomb 11 (Molina-Fajardo and Huertas-Jiménez 1983, 1985). However, the pottery is more diagnostic from a chronological point of view. The presence of a fragment of glass bulb unguentarium and fragments of vessels, including a Campanian patera (Morel 2255/Lamboglia 5), has been used as an argument to date this tomb to the first century BCE. Regarding Tomb 25, we do not have information about it. In any case, it is worth noting that Hellenistic unguntaria are frequent in the tombs of this area (D). Finally, although in that time (first century BCE) the normative funeral rite in the south of the Iberian Peninsula was cremation (Jiménez-Díez, 2009), Puente de Noy provides inhumations.

References

Huertas Jiménez C, Molina Fajardo F (1983) Tipología de la cerámica de la necrópolis fenicio-púnica de Puente de Noy en Almuñécar (Granada). In: XVI Congreso Nacional de Arqueología (Cartagena, Murcia, 1982). Zaragoza: Depto. de Ciencias de la Antigüedad, pp. 497-506.

Jiménez-Díez A (2009) Roman Settlements / Punic Ancestors. Some Examples from the Necropoleis of Southern Iberia. In: M dalla Riva (ed.), Meetings between Cultures in the Ancient Mediterranean. Proceedings of the 17th International Congress of Classical Archaeology (Rome 22-26 sept. 2008): 25-43. Rome, International Association for Classical Archaeology. Published at Bolletin di Archeologia On Line, Volume Speciale: http://bollettinodiarcheologiaonline.beniculturali.it/wp-content/uploads/2019/01/4_Jimenez_paper.pdf

Molina-Fajardo F, Huertas-Jiménez C (1983) Tipología de las tumbas de la necrópolis feno-púnica de Puente de Noy (Almuñécar, Granada). In: XVI Congreso Nacional de Arqueología (Cartagena, Murcia, 1982). Zaragoza: Depto. de Ciencias de la Antigüedad, pp. 489-496.

Molina-Fajardo F, Huertas-Jiménez C (1985) Almuñecar en la Antigüedad. La necrópolis fenicio/púnica de Puente de Noy II. Granada: Excma. Diputación Provincial de Granada/Ilmo. Ayuntamiento de Almuñécar.

Hipogeo del Parking de Alcazabilla (Málaga, Spain)

Juan Manuel Jimenez Arenas, Enrique Viguera, Sonia López Chamizo and José Suárez Padilla

General Location and Chronology: The site is located within the historic center of Málaga. It lies on the left bank of the Guadalmedina River (36º 43' 20.09'' N / 4º 24' 58.63'' W) and dates to the 4th to 6th century BCE.

Excavation history: The tomb was discovered in 2000 when a public parking lot was built on the west slope of Alcazaba Hill. This hypogeum is part of a necropolis from the Phoenician period. In this area, scattered burials have been located since the 6th century BC, presenting various traditions and typologies (cremation pits, individual burial tombs, or a hypogeum).

Description of cemeteries: The rectangular chamber dates to the 6th to 4th century BC. It has partially been destroyed and has masonry walls and an ashlar entrance. It is about 4.5 m long and about 3 m wide, with an estimated surface of 12-13 m², which seems to have had an opening in its rear that would have been destined for the reception of libations from abroad. Inside, four buried individuals were found, three of them inside graves, two males of about 25 and 50 years, respectively, and a woman of a similar age.

The three individuals deposited in the graves carried personal adornments, including gold earrings. Both inside and outside the burial, a copper bracelet was found, as well as the remains of amphorae, some of them Iberian; bowls, mortars, jugs, and plates, sometimes decorated with red slip (that can show some Phoenician graphite), all of them of Punic tradition; and some fragment of an attic black-glazed stamped bowl.

Burnt bones of an ox, a goat, and a bird were found outside the hypogeum, but according to the analysis carried out, these were not consumed so that they could be interpreted as an offering to the spirit of the deceased. In addition, the remains of a dog were discovered at the base of the chamber that had also been burned and, this time, beheaded, possibly being a propitiatory ritual.

References

Martín Ruiz, J.A.; Pérez-Malumbres Lada, A.; Carretero, J.R. (2003): Tumba de cámara de la necrópolis fenicia de Gibralfaro (Málaga, España); (Anexo I: Mabel Montero, Análisis de los restos de fauna; Anexo II: Alfonso Palomo Laburu y Víctor Smith Fernández, Análisis de los restos antropológicos) Rivista di Studi Fenici, 31, nº 2: 139-160.

Martín, J.A. (2012): "El hipogeo fenicio de Mundo Nuevo (necrópolis de Gibralfaro, Málaga), E. García (ed.), Diez años de arqueología fenicia en la provincia de Málaga (2001-2010), Junta de Andalucía, Monografías-Arqueología: 105-120.

Calle Mármoles (Málaga, Spain)

Juan Manuel Jimenez Arenas, Enrique Viguera, Sonia López Chamizo and José Suárez Padilla

General Location and Chronology: The site is located within the city of Málaga on the right bank of the Guadalmedina river. It dates to the 4th to 3rd centuries BCE.

Excavation history: The tomb was located due to a preventive archaeological activity in 2010. This hypogeum is part of a necropolis from the Phoenician period located on the right bank of the Guadalmedina River. In this area, scattered burials have been located since the 6th century BC, which, as in the necropolis located on the left side of the Guadalmedina River), present different traditions and typologies (cremation pits, individual burial tombs, or a hypogeum).

Description of cemeteries: The dromos or access corridor has been preserved. It is excavated in the rock and ends in a rectangular trend chamber (or even in the shape of a bull's skin) built with masonry (with approximate dimensions of 3.5 by 1.50 m). The access door, which was found sealed, was built partly with ashlars and possibly reused. One has a perforation, which could be used ceremonially to pour liquids into the tomb. Stone slabs of considerable dimensions, arranged on two sides, were used to create the chamber ceiling.

Inside the tomb, three levels of burial have been located. Except in the intermediate layer, where there are remains of an articulated individual, they are removed. On one of the interior walls, there is a funerary deposit associated with a Kouass-type lamp, which dates the final use of the complex to the 3rd century BC. There is no anthropological study of the skeletal remains.

The grave goods are concentrated in the access corridor. Fragments of vessels, jugs, and bowls of Punic tradition appear, together with a piece of an Iberian-type plate dated between the 5th and 4th centuries BCE. Added to these findings is a necklace bead, which could have been part of some individuals' personal adornments.

Next to the door, there is a pit whose contents relate to the practice of a foundational banquet. Its materials provide a chronology of the tomb's construction in the second half of the 6th century BCE.

References

Florido, D., Navarrete, V., Ramírez, J.D., Ruiz, N. y Sabastro, M.A. (2012): “Un hipogeo con forma de piel de toro a orillas del Guadalmedina. Málaga”, E. García (ed.), Diez años de arqueología fenicia en la provincia de Málaga (2001-2010), Junta de Andalucía, Monografías-Arqueología: 121-136.

Tharros (Sardinia, Italy)

Anna Chiara Fariselli and Carla Del Vais

General Location: The Northern necropolis of San Giovanni / Santu Marcu is located along the western coast of the Sinis peninsula, north of the urban settlement of Punic Tharros on the Gulf of Oristano. Conversely, the Southern necropolis is located on Capo San Marco at the end of the Sinis peninsula.

Excavation history: The northern funerary area has been known since the nineteenth century. A map, on a scale of 1:5000 created between 1884 and 1885 by Filippo Nissardi, indicates numerous tombs that evidently had already been violated. At the end of this century (1891-1893), the lawyer Efisio Pischedda of Oristano carried out excavations in the area of Tharros and probably also in the northern necropolis, resulting in the recovery of rich funeral offerings. In the same years, incineration tombs were identified on the occasion of constructing a domed building (Area B).

Throughout the first half of the twentieth century, there is no news of findings in the funerary area. Destructive intervention perpetrated in 1947 by some local stone quarrymen was interrupted by the Superintendence of Cagliari. Starting from the end of the fifties, with the planting of the village of San Giovanni di Sinis, the necropolis was partly covered and suffered significant interventions of destruction and looting. In 1958 an intervention was carried out by the Superintendence of Antiquities, under the direction of Gennaro Pesce, in an area where a house was to be built (Area A).

In the same area, Giovanni Tore (University of Cagliari, 1989-1991) conducted long and fruitful investigations, documenting more than fifty Punic tombs excavated in the rocky bank (chamber and pit tombs). In 2001, the same sector was the subject of an enhancement project. The research continued in the years 2009-2013 under a five-year excavation concession to the University of Cagliari (DG Prot. 2145, Class. 34.31.07/382.1, 2/03/2009; sc. dir. C. Del Vais), in collaboration with the University of Bologna. Four funerary areas dating to the Punic period have been investigated (Areas A-C). A particular focus was on Area A and Area B, from which most of the bone remains analyzed here originate. In all sectors, secondary (and more rarely primary) incineration pit tombs dug in the sandbank (late 7th-6th cent. BCE.) and parallelepiped and chamber tombs dug in the sandstone bank (6th-3rd cent. BCE) have been documented. In some sectors, pit tombs of the Roman-Republican age (last third of the III century BCE.-I century BCE.) have also been identified for incineration and inhumation.

Description of cemeteries: The San Giovanni/Santu Marcu Necropolis, or Northern Necropolis, features cremation tombs made in shallow pits dug in the sand dating from the 7th-6th centuries BCE. It also houses parallelepiped pits dug into the rock and chamber tombs accessible from stepped shafts, the latter two types with inhumation burials. Hypogean rock-cut tombs, both pit and chamber tombs, are set up from the late 7th century BCE, especially in the 6th-2nd century BCE. On the other hand, tombs from the Roman period are made of simple earthen pits covered with some lithic slabs from the 1st century

BCE to the 2nd century AD, according to the present data. It is not uncommon for the Romans to destroy Punic tombs either to obtain new deposition spaces for their dead or because, in the imperial age, a large part of the northern necropolis was used as a stone quarry. Some rock-cut Punic tombs and even chamber tombs, however, are likely to have been reused in the Roman phase as well, as evidenced by pottery.

The necropolis of San Giovanni / Santu Marcu belongs to the Punic city of Tharros, but unlike the Southern Necropolis, the Northern seems to be used by the community that lived near the lagoon port of Mistras, located a short distance away. This is probably the cemetery context reserved for people associated with the port trades. The Southern cemetery of Capo San Marco instead is the largest and most monumental necropolis, reserved for the uses of the citizen community and the Carthaginian ruling class.

Ancient literary sources speak of the existence of two Tharros: one related to commercial activities and the other more related to manufacturing, civic, and leadership class activities. Nor is it anomalous to think that travelers and merchants have settled in Tharros / Mistras and have adopted the local funeral customs, something completely customary and documented throughout the Punic Mediterranean, where the phenomena of integration, also testified by name inscriptions, are frequent.

None of the samples described above belong to closed contexts with precise associations of grave goods because the whole Northern necropolis, like the Southern one, was violated by the inevitably tampering with the original Punic tombs.

References

C. Del Vais, C. – Fariselli, A.C. (2012) *La necropoli settentrionale di Tharros: nuovi scavi e prospettive di ricerca (campagna 2009)*, *ArcheoArte*, suppl. 1 (2012), pp. 265-283.

Del Vais, C.. – Fariselli, A.C. (2019), *Nuove ricerche nella necropoli settentrionale di Tharros (S. Giovanni di Sinis, Cabras OR)*, in A. Ferjaoui – T. Redissi (edd.), *La vie, la mort et la religion dans l'univers phénicien et punique. Actes du VIIème congrès international des études phéniciennes et puniques Hammamet, 9 - 14 novembre 2009*, Tunis, pp. 1239-1260.

Del Vais, C. - Fariselli, A.C. (2022), *Tharros*, Sassari.

Fariselli, A.C. (2021), 'Rituali collettivi ed escatologia privata nel paesaggio funerario tharrense: dati dalla necropoli punica di Capo San Marco (Penisola del Sinis)', *Actas del XI Coloquio Internacional del CEFYP: la muerte y el más allá entre Fenicios y Púnicos (27-29 de noviembre 2019)*, Eivissa, pp. 273-288.

Akhziv (Israel)

Patricia Smith and Marina Faerman

General Location and Chronology: Tel Achziv (also Achzib, Akhziv, or el Zib) is located on a sandstone (kurkar) ridge on the northern Mediterranean coast of Israel, ca. 15 km north of Acre. It was first settled in the Bronze Age and was a prosperous port between Iron Age II and the Roman Period (11th Century BCE -4th Century CE). Four Iron Age cemeteries lie around the tel containing tombs, a crematorium, and cinerary urns. The eastern slopes of the tel contained the earliest tombs. They are rectangular cist tombs dug into the local sandstone and aligned north-south, whereas all tombs in the other cemeteries were aligned east-west. The tombs in the southern cemetery include one cist tomb, four rectangular chamber tombs constructed at the end of the 11th-early 10th centuries BCE but used intermittently through to the 7th century; shaft tombs constructed in the 9th-7th centuries BCE, and shallow pits with cinerary urns, containing cremated remains from the 10th-6th centuries BCE The northeastern extension of the southern cemetery (area E) contains additional shaft tombs and cinerary urns in shallow pits as well as pit tombs and cist tombs dating to the 7th-4th centuries BCE. The northern cemetery was primarily used for cremation. It contained a crematorium and numerous cremation urns in pits, some with engraved steles as well as pit tombs and infant burials from later periods, but also contained one chamber tomb (TN1), similar to those identified in the southern cemetery.

Excavation history: Excavation of the cemeteries began with the excavations of I. Ben-Dor between 1941-1944 (Dayagi-Mendels 2002), followed by M. Prausnitz between 1957-1984 (Prausnitz 1969, 1975, 1982, 1986, 1993) and E. Mazar 1988-2004 (Mazar (2000, 2001, 2004, 2008, 2009). Most of the tombs contained multiple burials as well as valuable grave offerings that included red-slipped ceramic figurines, masks, scarabs, ivories, seals, jewelry, and a variety of iron ornaments and implements, including swords, daggers, scythes, and arrowheads.

Description of cemeteries: The skeletal remains sampled for DNA come from multiple, successive burials in chamber tombs TC1, 2, and 4 in the southern cemetery and TN1 in the northern cemetery. All were constructed from the local sandstone and were similar in size, measuring between 2.70-2.86 m long, 1.65-1.86 m wide, and ~1.65-1.75 m high, with a small rectangular entrance in the eastern wall. TC4 lacked a roof and a dromos, both of which were present in the other three tombs, and was built with unfinished sandstone blocks in contrast to the other three that were built with ashlar blocks, while each of the roofed tombs had a hole cut into the roof that was used for libations. All four were broadly contemporaneous and were used intermittently for burial between the end of the 11th-7th centuries BCE, with earlier burials either pushed aside to make room for later burials or simply stacked on top of one another with their associated grave offerings so that the latest burials were in the upper levels. Some were still articulated, indicating that primary burials were the norm. They had been buried in an extended position. They included adults of both sexes and children but no infants. Most of the crania sampled for DNA analysis originate from the upper, later burials.

TC1 locus 979 contained disturbed skeletal remains of at least 50 individuals that had been piled in heaps around the sides of the tomb to make room for later burials (Smith, Horwitz

and Zias1990). DNA was recovered from the skeleton of a young woman (ASC-9) found in the upper level of a bone pile in the northeastern corner of the tomb and another adult (ASC-15).

TC2 (loci 212, 215) contained several crania in a pit (locus 215) dug inside the entrance (one of these yielded DNA: ASC-8, locus 215), as well as 40 additional individuals and grave offerings that included scarabs and cylinder and stamp seals dated to the 9th and 7th centuries BCE DNA was recovered from three fragmentary crania found in the upper burial level of locus 212: ASC-10, ASC-13, and ASC-17.

TC4 (locus 610) contained 50 individuals in bone piles (Smith et al. 1993), and DNA was retrieved from four of them in the lower levels: ASC-2 (and ASC-2B), ASC-3 (and ASC-3B), ASC-4 and ASC-11.

(TN1) in the northern cemetery was covered by a sandbank, so the entrance was sealed and damp when excavated. It had a larger dromos than those in the southern cemetery and a gabled roof (Mazar 2004:195-197). The excavation was complicated by the fact that the tomb was damp, so the bones were poorly preserved, and only 4 of the 39 petrous bones sampled yielded DNA. All were from the upper level of burials that were attributed to the late 7th – mid-6th centuries BCE: ANC-19, ANC-31 (and ANC-42), and ANC-3B.

To summarize, DNA was recovered from 16/62 petrous bones sampled from the chamber tombs.TN1 in the northern cemetery was least successful, with DNA recovered from only 4/39 petrous bones sampled compared with 12/23 petrous bones sampled from chamber tombs in the southern cemetery. The only difference noted between them was that associated with the microenvironment (damp versus dry).

References

Dayagi-Mendels, M. 2002. Akhziv Cemeteries: The Ben-Dor Excavations, 1941-1944. Israel Antiquities Authority.

Mazar, E. 2000. Phoenician Family Tombs at Achziv, A Chronological Typology (1000-400 BCE). in Gonzalez Prats, A. (ed.) Fenicios y Territorio, pp.189-225. Alicante.

Mazar, E. 2001.The Phoenicians in Achziv, The Southern Cemetery. Cuadernos de Arqueología Mediterránea 7.

Mazar, E. 2004.The Phoenician Family Tomb T.N.1 at the Northern Cemetery of Achziv (10th – 6th centuries BCE). Cuadernos de Arqueología Mediterránea 10.

Mazar, E. 2008. Changes in Phoenician Burial Customs from the Assyrian to the Persian Period, in light of Achziv Excavations. In Bar, S. (ed.) In the Hill-Country, and in the Shephelah, and in the Arabah (Joshua 12, 8): Studies and Researches Presented to Adam Zertal in the Thirtieth Anniversary of the Manasseh Hill-Country Survey, pp.146-151. Jerusalem (Hebrew).

Mazar, E. 2009. Phoenician Burial Customs at Akhziv. Qadmoniot 138: 93-103. (Hebrew)

Prausnitz, M.W. 1969. Israelite and Sidonian Burial Rites at Akhziv. In Proceedings

of the Fifth World Congress of Jewish Studies. Jerusalem. Vol I, pp. 85-89.

Prausnitz, M. W. 1975. The Planning of the Middle Bronze Age Town of Achzib and Its Defences. Israel Exploration Journal 25: 202–210.

Prausnitz, M.W. 1982. Die Nekropolen von Akhzif und die Entwicklung der Keramik vom 10. Bis zun 7. Jarhlhundert V. Chr. In Niemeyer H.G. (ed.), Akhziv, Samaria und Ashdod, (Phonizier im Westen Madrider Beitrage 81), Mainz am Rhein, pp. 31-44. Pls. 2-3.

Prausnitz, M.W 1986. Bamot Hatofet. In Yedaya M. (ed.), The Western Galilee Antiquities, Tel-Aviv, pp. 466-468 (in Hebrew).

Prausnitz, M.W 1993. Achzib. In Stern E. (ed.), The New Encyclopedia of Archaeology in the Holy Land, Jerusalem, pp. 32-36.

Smith, P., Horwitz, L. and Zias, J. 1990. Human Remains from the Iron Age Cemeteries at Akhziv. Part 1: The Built tomb from the Southern Cemetery. Rivista di Studi Fenici, XVIII: 137-150.

Smith, P., Mazar, E., Sabari, P., Selah, M. and Ganchrow, R. 1993. The Early Phoenicians, Excavated from Achziv, Northern Israel. National Geographic Research and Exploration 9(1): 54–69.

Motya (Sicily, Italy)

Dalit Regev, Francesca Oliveri, Pamela Toti

General Location and Chronology: The island of Mozia (Motya) in Western Sicily is a unique Phoenician settlement in the Western Mediterranean. Its exceptional state of preservation offers insights into the topographical development of the settlement, its various phases of existence, and its funerary practices in close proximity to the urban area. The residential areas and burial grounds at Mozia were clearly demarcated by walls from the mid-6th to the late 4th century BC, similar to cases in the Phoenician homeland of Tyre and Arwad (Elayi, 1996). The archaic necropolis of Mozia is situated along the island's northern coast on a rocky plateau approximately three meters above the shoreline. While the exact boundaries of the necropolis remain partly uncertain, the earliest phase extended over a minimum length of 60 meters in an east-west direction, roughly positioned between the two wall towers. The investigations indicate that it extended beneath a section of the fortification wall and, in some areas, even a few meters inside it. The cemetery area lacks distinct ancient enclosures but was physically separated or intentionally distanced from the rest of the settlement.

Excavation History: Archaeological excavations at Mozia began in the early 20th century (1906-1913) by Giuseppe Whitaker in collaboration with Antonio Salinas from the Palermo Museum (Pace, 1915; Whitaker, 1921). These excavations unearthed tombs with grave goods dating from the 7th and 6th centuries BC. Systematic investigations were later conducted by Vincenzo Tusa in the 1970s, focusing on incineration tombs of the Archaic period, particularly in the so-called "Luogo di Arsione" or Industrial Quarter (Bevilacqua et al. 1972; Ciasca et al. 1973; Ciasca 1978; Ciasca et al. 1978; Ciasca 1990; Tusa, 1983). The incineration necropolis of Mozia, also known as the "Whitaker Necropolis" and the "Archaic Necropolis," predominantly contains tombs from the 6th century BC with Phoenician-style

pottery, including Red Slip and painted vessels, common pottery, and imported Greek pottery. In 1955, archaeologists from the British Mission headed by B.S.J. Isserlin discovered a lithic sarcophagus with an inhumation burial and grave goods dating to the late 6th century BC, challenging the assumption that burials in Mozia shifted to Birgi in the mid-6th century BCE (Isserlin et al. 1958). Instead, it suggests that the area close to the walls on the north and east sides of the island served as a cemetery from the mid-6th to the 4th century BCE, occupying a larger area than the earlier archaic necropolis.

Description of Cemeteries: The island's funerary archaeology has revealed ca. 350 graves, showcasing both inhumation in lithic sarcophagi and incineration in terracotta urns (Giammellaro, 2004; Nigro 2003; Nigro 2004; Spagnoli 2008). Recent investigations by Maria Grazia Griffo at the Birgi site, along with the study of materials from the same necropolis preserved in the Whitaker Collection of the Museum of Mozia by Maria Luisa Famà and Maria Pamela Toti, revealed the chronological independence of the two necropolises, dating the grave goods starting in the 7th century BCE (Oliveri and Toti, forthcoming). During the 6th century BCE, the designated necropolis area expanded, potentially encompassing the island's entire perimeter. The construction of surrounding walls in the latter half of the 6th century BCE fully incorporated the extensive cemetery area. It is important to note that this does not imply a relocation of the necropolis to the mainland, as new burials could still occur on the island's beaches despite certain sections being cut off or covered by the fortified wall. Evidence from the 5th century suggests the continuity of life in Mozia even after the destruction caused by Dionysius, including a small burial area in use from the 4th to 2nd centuries BCE. This area, situated alongside the eastern bastion of the North Gate and the Sanctuary of Cappiddazzu, is the only part of the Mozia settlement where evidence of a presence during the Imperial Roman period and beyond has been found. In recent campaigns (2013-2017), the University of Palermo excavated the archaic necropolis of Mozia, uncovering a total of 115 graves (Sconzo 2020). The variety of burial rituals observed, including inhumations, cremations, and mixed or atypical burials, reflects the complexity and changing of Phoenician funerary practices on the island.

References

Bevilacqua, F., A. Ciasca, G. Matthiae Scandone, S. Moscati, V. Tusa, and A. Tusa Cutroni. 1972. Mozia - VII. Rapporto preliminare della Missione congiunta con la Soprintendenza alle Antichità della Sicilia Occidentale (Studi Semitici 40). Roma.

Ciasca, A., V. Tusa, and M.L. Uberti. 1973. Mozia - VIII. Rapporto preliminare della Missione congiunta con la Soprintendenza alle Antichità della Sicilia Occidentale (Studi Semitici 45). Roma.

Ciasca, Antonia. 1978. "Mozia 1977: Scavi alle mura (campagna 1977)." Rivista di Studi Fenici Roma 6 (2): 227-244.

Ciasca, A., G. Coacci Polselli, N. Cuomo Di Caprio, M.G. Amadasi Guzzo, G. Matthiae Scandone, V. Tusa, A. Cutroni Tusa, and M.L. Uberti. 1978. Mozia - IX. Rapporto preliminare della Missione congiunta con la Soprintendenza alle Antichità della Sicilia Occidentale (Studi Semitici 50). Roma.

Ciasca, A. 1990. “Sulle necropoli di Mozia.” Sicilia Archeologica 72: 7-11.

Elayi, Josette, and Muhammad Raif Haykal. 1996. Nouvelles découvertes sur les usages funéraires des Phéniciens d'Arwad. Paris: Gabalda.

Giammellaro, Spanò A. 2004. "I luoghi della morte: impianti funerari nella Sicilia fenicia e punica." In El mundo funerario, edited by A. Gonzalez Prats, Alicante, 205-251.

Isserlin, B. S. J., W. Culican, W. L. Brown, and Aldina Tusa Cutroni. "Motya: 1955." Papers of the British School at Rome 26 (1958): 1-29.

Nigro, L. 2003. "Nuovi scavi a Mozia dell'Università di Roma 'La Sapienza' (XXII campagna, agosto-ottobre 2002)." Sicilia Archeologica 101: 85-97.

Nigro, L. (ed.). 2004. Mozia X: Zona C. Il Kothon. Zona D. Le Pendici Occidentali Dell'acropoli. Zona F. La Porta Ovest, Rapporto Preliminare Della XXII Campagna Di Scavi, 2002. Quaderni Di Archeologia Fenicio-Punica 1. Roma: Missione archeologica a Mozia. Roma.

Oliveri, F., and M.P. Toti. "Mozia oltre...testimonianze archeologiche ed archivististiche sulle fasi tarde dell'isola." In Oltre gli Elimi, edited by Fondazione Ettore Maiorana, 7-8 Aprile 2022, Erice. (in press)

Pace, B. 1915. "Mozia. Prime note sugli scavi eseguiti negli anni 1906-1914." NSc, 1915: 431-446.

Sconzo, P. 2020. "Nuovi dati dalla necropoli arcaica di Mozia (campagne 2013-2017)." IX Congreso Internacional de Estudios Fenicios y Púnicos / International Congress of Phoenician and Punic Studies MYTRA 5, 2020: 1205-1217.

Spagnoli, F. 2008. "Sepolture intramurali a Mozia." In Atti Del Convegno Internazionale Sepolti Tra I Vivi - Buried Among The Living. Evidenza ed interpretazione di contesti funerari in abitato, edited by G. Bartoloni and M. G. Benedettini, Roma, 26-29 Aprile 2006.

Tusa, V. 1983. "La Sicilia fenicio-punica: stato attuale delle ricerche e degli studi e prospettive per il futuro." In Atti del I Congresso Internazionale di Studi Fenici e Punici (Roma 5-10 novembre 1979) (Collezioni di Studi Fenici 16), edited by P. Bartoloni, S.F. Bondì, G. Coacci Polselli, M.T. Francisi, F. Mazza, G. Petruccioli, P. Xella, Roma, 187-197.

Whitaker, J. I. S. 1921. Motya, a Phoenician colony in Sicily. London: G. Bell.

Motya (Sicily, Italy) University of Palermo excavations in the archaic necropolis

Paola Sconzo

Most of the samples provided for this study were retrieved between 2014 and 2016 by the University of Palermo within Tower B8, also called Tower Whitaker, on the NW sector of the island. When in use, this tower undoubtedly dominated the central sector of the archaic necropolis. It consisted of a protruding structure with a rectangular plan, divided into two small chambers by a central partition (Sconzo 2020; Lauria et al. 2017; 2018; 2020). Here, beneath the floor levels, in archaeological layers clearly predating the construction of the city wall, a previously intact portion of the archaic necropolis has been uncovered, and around fifty undisturbed burials have been identified. These findings confirm the presence of a long stratification, which reaches a thickness of 2 meters and reveals at least eight layers of burials without reaching the bedrock (Sconzo 2020). Based on the rare grave goods found inside the funerary pits or in direct association with the deposits, on the burial urns

themselves and on the ceramic materials from the layers of use and accumulation, it was possible to infer that the most recent part of the necropolis area has also been preserved in this zone, with burials dating from the late 7th to the early 5th century BCE. Samples nos. 5 and 10 come from infant burials (Graves T.248 and T.260) in enkytrismoi dating to the V cent. BC; samples nos. 4 and 6 from adult inhumations (Graves T.235 and T.260) of uncertain dating. Samples nos. 7- and 8 come from deposits cut by the grave pits.

References

Lauria, G. – Sconzo, P. – Falsone, G. – Sineo, L. 2017. Human Remains and Funerary Rites in the Phoenician Necropolis of Motya (Sicily)", International Journal of Osteoarchaeology, 27: 1003–1011.

Lauria, G. – Sconzo, P. – Falsone, G. – Sineo, L. 2018. New Anthropological data from the Archaic Necropolis at Motya (2013 Excavation Season)". In M. Guirguis (ed.), From the Mediterranean to the Atlantic: people, goods and ideas between East and West. I. 8th International Congress of Phoenician and Punic Studies (Carbonia, Sant'Antioco, 21th-26th October 2013), Pholia Phoenicia 2: 250-252.

Lauria, G. – Sconzo, P. – Falsone, G. – Sineo, L. 2020. Child inhumations on the island of Motya. New evidence from the Archaic Cemetery. 9th International Congress of Phoenician and Punic Studies (Merida, 21th-26th October 2018): Mytra 5, 2020: 1781-1785.

Sconzo, P. 2020. Nuovi dati dalla necropoli arcaica di Mozia (campagne 2013-2017). 9th International Congress of Phoenician and Punic Studies (Merida, 21th-26th October 2018): Mytra 5, 2020: 1091-1102.

De Simone, R. 2023. Una Nuova Iscrizione Punica Su Un Cippo Funerario Da Mozia. Pholia Phoenicia 7: 53-61.

Peripoli, B. – Gigante, M. – Mahoney, P. – McFarlane, G. – Coppa, A. – Lugli, F. – Lauria, G. – Bondioli, L. – Sconzo, P. – Sineo, L. – Nava, A. 2023. Exploring prenatal and neonatal life history through dental histology in infants from the Phoenician necropolis of Motya (7th- 6th century BCE). Journal of Archaeological Science: Reports. https://doi.org/10.1016/j.jasrep.2023.104024.

Falsone, G. - P. Sconzo 2017. New Investigations in The North-East Quarter at Motya. The Archaic Cemetery and Building J", in M. Guirguis (ed.), From the Mediterranean to the Atlantic: people, goods and ideas between East and West. I. 8th International Congress of Phoenician and Punic Studies (Carbonia, Sant'Antioco, 21th-26th October 2013), Pholia Phoenicia 1: 62-69.

Sconzo, P. 2016. The Archaic Cemetery at Motya. A case-study for tracing early colonial Phoenician culture and mortuary traditions in the West Mediterranean". In F. Schön - H. Töpfer (eds.), Karthago Dialogue. Karthago und der punische Mittelmeerraum – Kulturkontakte und Kulturtransfers im 1. Jahrtausend vor Christus, Tübingen: 315-330.

Motya (Sicily, Italy) “Area K”

Luca Sineo and Francesca Meli

Sample "Motya 12" can be attributed to the remains of the T. 406 re-deposition. It is one of the few funerary attestations found in the northeastern sector of the island, well known as “Zona K”. This area is situated between the sanctuary of Capiddazzu and the fortifications. The University of Palermo conducted investigations between 1977 and 1981, as well as in 1985 (Falsone et 1980-81; id 1989; for eastern sector cf. Spanò Gemmellaro 1989). These investigations uncovered a section of an area that can be classified as "industrial” identified by the presence of two ceramic furnaces and several associated structures. During the investigations, several funerary artifacts were found: inhumations in simple pits, re-depositions (incinerations and inhumations), and/or simple bone clusters. These artifacts are likely associated with a temporary occupation after the destruction caused by Dionysius in 397 BC (Spanò Gemmellaro 1989, p.47, note 17; Falsone et al. 1989). Nevertheless, archaeologists have postulated that certain skeletal remains may have been primarily deposited in funeral units of the ancient necropolis adjacent to the island. (Falsone et al., 1980-1981, pages 877-930).

References

VV.AA., *La statua marmorea di Mozia e la scultura di stile severo in Sicilia*: Atti della giornata di studio, Marsala, 1 giugno 1986. L'Erma di Bretschneider, 1988.

G. Falsone, *I nuovi scavi di Mozia*, in BBCCAA Sic 1, 1980, pp. 98-103.

G.Falsone -F. Spatafora – A. Giammellaro Spano’ – L. Famà, *Gli scavi della Zona K a Mozia e il caso stratigratico del Locus 5615*, In Kokalos XXVI- XXVII, (1980-81), tomo II,2, pp. 877-930;

G. Falsone, *Struttura e origine orientale dei forni da vasaio di Mozia*, Fondazione Giuseppe Whitaker, 1981.

G. Falsone*, Mozia Zona k. La quarta campagna di scavo*. In *Sicilia Archeologica*, 1989, 71, pp. 51-63.

A. Spano Giammellaro, *Mozia: scavi nell'area K est-Campagna 1985. Notizie preliminari*, in *Sicilia archeologica*, 1989, 22.69-70, pp. 39-47.

Human remains from Motya city walls (Tower 1, Tower 4)

Lorenzo Nigro, Federica Spagnoli

A map of the north-east quarter of the island of Motya with the city-walls and the burial areas underneath them is depicted in from Nigro 2018, Fig. 9.
Two samples (104b and 106b - corresponding to our labels I22235 and I22236), refer to the skeletal remains of two individuals belonging to the first generations of Phoenician

inhabitants of Mozia. This is suggested by stratigraphy, as 104b and 106b were found in secondary burial, re-assembled together with many other human bones, within a sepulchral circular pit in which the remains of some archaic tombs dating back to the 8th century BCE or even earlier (some prehistoric burials were also included) had been collected, to make room for new burials (cremation in jars, dating to the 7th century BCE). Subsequently, the city walls were built over this round structure, and Tower 4, the first Motyan wall circuit, was erected there. The context of Tower 4, actually underneath Tower 4, is described in Spagnoli 2007-08 (327-328, Fig. 2, note 25 with references to the first publication of these findings by A. Ciasca). Human remains of the Phoenician (and prehistoric) burials were often found during the excavation of the city walls because the wall circuit, built around 550 BCE, cut through the pre-existing Phoenician and prehistoric cemeteries (Nigro - Spagnoli 2017, 59-69, Fig. 31). At that time, people buried surely belonged to the élite of the society, and may include members of the first families of Phoenicians (from the Levant and Cyprus) who first settled down on the island.

Recent excavations along the city walls, on the same north-western shore of the island, have revealed in Tower 6 (some 100 m to the west) other burials cut through by the wall structures, which yielded human remains and parts of tomb furnishings, including a monumental inscription of a tomb (Nigro 2019).

Sample 101 was found between Tower 1 and the later East Tower within Wall M.2 (Nigro 2020, 16, Fig. 3-4), i.e., the earliest city wall, that means a burial of the first half of the 6th century BCE, as also suggested by associated pottery.

References

NIRGRO, L. 2018, "*La Sapienza a Mozia 2010-2016: il primo insediamento fenicio, l'area sacra di Baal e Astarte, il Tofet, la necropoli, l'abitato, i nuovi scavi alle mura – una sintesi*": Fabrizio Serra (eds.), Folia Phoenicia, Pisa - Roma 2018, pp 484

NIGRO, L. 2019, "Qui riposa il «Servo di Melqart»": Archeo 418 (Dicembre 2019), pp. 42-46. [ISSN 1120-4559].

NIGRO, L. 2020a "*Sulle mura di Mozia. Stratigrafia e cronologia alla luce dei nuovi scavi della Sapienza (2014-2019)*": P. BARTOLONI - M. GUIRGUIS (eds.), Folia Phoenicia 4, Pisa - Roma 2020, pp. 13-64. [ISSN 2532-6384; e-ISSN 2532-7704] https://bit.ly/2M5DPdI

NIGRO, L. 2020n "Mozia, scavi alle mura (2014-2019)", S. DE VINCENZO (ed.), *Analysis Archaeologica. An International Journal of Western Mediterranean Archaeology* (Vol. 5 - 2019), Roma 2020, pp. 21-42, 298-303. [ISSN 2421-6380; ISBN 978-88-5491-099-7]

NIGRO, L. – SPAGNOLI, F. 2017a, *Landing on Motya. The earliest Phoenician settlement of the 8th century BC and the creation of a West Phoenician cultural identity in the excavations of Sapienza University of Rome - 2012-2016* (Quaderni di Archeologia Fenicio-Punica, Colour Monograph 04), Roma 2017. [ISBN 9788898154005; ISSN 1824-4017] https://bit.ly/36qTNFr

SPAGNOLI, F. 2007-2008 "Sepolture intramurali a Mozia", in G. BARTOLONI - M.G.

BENEDETTINI (a cura di), *Atti del convegno internazionale "Sepolti tra i vivi: evidenza ed interpretazione di contesti funerari in abitato", Università di Roma «La Sapienza», 26-29 aprile 2006* (Scienze delle Antichità, 14), Roma 2007-2008, pp. 323-346.

Birgi (Sicily, Italy)

Dalit Regev, Francesca Oliveri, Pamela Toti

General Location and Chronology: The necropolis of Birgi, located on the northern coast of the Stagnone of Marsala, was connected to the island of Mozia by a road (that is now submerged) ending at the North Gate. The necropolis was used from the late 8th to the mid-3rd centuries BC.

Excavation History: Archaeological discoveries are known since the late 1800s (Whitaker, 1921). Between 1996 and 2004, extensive stratigraphic investigations were carried out in Birgi, with significant excavations from 1996 to 1999 (Griffo, 1997). Within approximately 700 m², the largest stratigraphically excavated area to date, 140 tombs of three different types were uncovered: incineration, sarcophagus inhumation, and enchytrismos. In most cases, the grave goods were found in situ.

Description of Cemeteries: The distribution of incineration burials does not exhibit any discernible spatial planning or groupings. However, the sarcophagi were divided into three sets, each with a different orientation: northeast/southwest, north/south, and east/west. Notably, the discovery of a sarcophagus superimposed on an older one within the latter group represents the first known instance of this funerary custom.

The burials from the first phase involved incineration within ollae or amphorae of Phoenician tradition and can be dated between the late 8th and mid-6th centuries BC. In the second phase, which spanned from the second half of the 6th to the 5th centuries BC, a higher quantity of tombs combining incineration and inhumation within sarcophagi indicates a population increase in the settlement, parallel to the findings in Motya. In the third and final phase, from the 4th to the mid-3rd centuries BC, the use of incineration resurfaced. Regarding infant burials, preliminary analyses of human remains have identified ten, with the oldest dating back to the late 6th century BC. The containers used were always amphorae, which is a Phoenician tradition.

Significant damage has been inflicted on the burials, particularly those from the last phase, due to extensive agricultural activities in the area since the late 1800s. Mechanized plowing since the 1960s has further impacted the site. The plastic clay soil has chemically reacted with the mixture of numerous vessels, leading to the disappearance of painted surfaces and the disintegration of forms. Identification of artifacts often relies on imprints left in the clay. The sarcophagi bottoms exhibit furrows from plowing, and the long sides sometimes collapse inwardly due to soil pressure.

The grave goods display a highly diverse composition in quantity and types. Phoenician materials resemble those found in the necropolis of Mozia, except for locally produced skyphoi imitating Greek forms, which are characteristic of the Mozia necropolis. Furthermore, no significant quantitative differences were observed between Phoenician and Greek vessels at Birgi, unlike the findings at Mozia. Additionally, three funerary inscriptions in the archaic Greek alphabet have been documented, two of which are preserved at Mozia

and one in Palermo, affirming that these sites are two separate but parallel entities (Fama and Toti, 2019; Griffo, 2009).

References

Famà, Maria Luisa, and Maria Pamela Toti. "La Necropoli Di Birgi: Un Esempio D' interazione Culturale Tra Fenici E Greci Nell'eterno Banchetto." In Nel Mondo Di Ade : Ideologie, Spazi E Rituali Funerari Per L'eterno Banchetto (Secoli Viii-Iv A.C.) : Atti Del Convegno Internazionale, Ragusa-Gela, 6-7-8- Giugno 2010, edited by Rosalba Panvini and Lavinia Sole, 395-409. Caltanissetta: Salvatore Sciascia editore, 2019..

Griffo, M.G. 1997. "Birgi, la riscoperta di un sito archeologico." In Marsala, edited by M.G. Griffo, 40-42. Marsala: Murex Edizioni.

Griffo, M.G. 2009. "Per la conoscenza di una fase arcaica della necropoli di Birgi attraverso i reperti della collezione Whitaker." In La Sicilia in età arcaica, dalle apoikiai al 480 a.C. Contributi delle recenti indagini archeologiche, edited by R. Panvini and L. Sole, 273-275.

Whitaker, J. I. S. 1921. Motya, a Phoenician colony in Sicily. London: G. Bell.

Lilybaeum, Corso Gramsci necropolis (Sicily, Italy)

Anna Chiara Fariselli, Donata Luiselli, Elisabetta Cilli

General Location and Chronology: according to classical authors (Diod. Sic. 22.10.4.), in 397 BC, the Phoenician city of Motya, situated on the island of San Pantaleo at the center of a large lagoon, known today as 'Stagnone', was invaded and destroyed by the Syracusan tyrant Dionysus I. The survivors founded a town on the mainland nearby, on the promontory of Capo Boeo, that they called Lilybaeum, which developed into the most important military stronghold in Punic Sicily. Based on other accredited hypotheses, the foundation of the city, equipped with various port basins and naturally fortified, would have been designed by Carthage to build a solid military base in a highly strategic context of the central Punicized Mediterranean (Acquaro, 2014).

Excavation History: the excavation of the sector of the necropolis located in Corso Gramsci, conducted from spring 2003 to summer 2004, uncovered fifty-seven rock-cut rectangular pit tombs and five hypogeic chamber tombs. These have burial cells carved out on opposite sides of the stepped access shaft. The necropolis was reused in the early Christian period. The typical rock-cut Punic graves either have a vertical shaft leading into one or more funerary chambers (type I) - probably burial chambers corresponding to family hypogea - or mostly consist of a rectangular cist (type II), inside which the body of the deceased was deposited on a wooden support. Human remains have been found in most tombs, not in anatomical connection and in extremely fragmented conditions. Although rare, the ritual of incineration was practiced: after the individual's cremation, the skeletal remains were placed into terracotta urns deposited inside rock cavities or in stone shelves provided with a lid (Di Salvo, 2004).

References

Acquaro, E. (2014), La Cartagine di Elissa e le sue rifondazioni nel Mediterraneo, Scienze e Ricerche 1, pp. 245-247.

Bechtold, B. (1999), La necropoli di Lilybaeum, Roma.

Di Salvo, R. (2004), Antropologia e paleopatologia dei gruppi umani di età fenicio-punica della Sicilia occidentale, in Pràts G (ed.), El mundo Funerario, Alicante, pp. 253-258.

Giglio, R. (2016), 'La necropoli di Lilibeo alla luce delle recenti scoperte', Lattanzi, E. – Spadea R. (eds.), Se cerchi la tua strada verso Itaca …Omaggio a Lina Di Stefano, Roma 2016, pp. 101-114.

Lilybaeum, the Tribunale and Monumentale Necropolis (Sicily, Italy)

Dalit Regev, Francesca Oliveri, Pamela Toti

General Location and Chronology: The necropolis of Lilybaeum extended along the eastern side of the city, starting from the outer edge of the moat. It stretched from the rocky ridge, currently occupied by the slaughterhouses and wine factories, to the Salinella contrada in the north. In the south, it reached the Madonna della Grotta area, which contains remains of early Christian catacombs, occasionally reusing Phoenician tombs. These excavations have provided valuable insights into Phoenician Sicily during the 4th-3rd centuries BC (Bechtold et al. 1999; Di Stefano, 1984, 1993).

Excavation History: Fortuitous discoveries in this area have been reported since the 18th century. However, systematic excavations began in the late 19th century and intensified over the last thirty years due to significant urban expansion. The area around the present-day Tribunal exhibits the highest concentration of necropolis remains. Systematic excavations since 1948 have allowed for an overall reconstruction of the site. Other extensive areas explored include Via del Fante, Massimo D'Azeglio, and A. De Gasperi (Bisi 1966, 1967, 1970; Di Stefano 1974).

Description of Cemeteries: The tombs in the calcarenite bank display a distinctive typology typical of Phoenician cemetery areas. The most common burial type is a simple uncovered rectangular pit, averaging 1.80 to 2 meters long, 0.70 meters wide, and up to 1 meter deep, designed for individual burial. These graves primarily contain adults, occasionally including smaller graves for children. Adjacent to the pit tombs, vertical shaft hypogea openings were present where the terrain allowed. These hypogea in Lilybaeum could reach depths of up to 10 meters, leading to one or two burial chambers at the bottom. The rectangular chambers lacked decoration but displayed prominent traces of work tools on the walls. Another burial type consisted of simple pits without chambers. These graves had three or four slabs serving as covers, resting on a fold, with an average depth of about 2 meters. They contained multiple burials, likely belonging to the same household. Isolated cinerary urns deposited in natural rock cavities with modest grave goods were common in some areas of the necropolis (Bechtold and Valente, 1992).

The hypogea were likely reserved for households of higher social status. Over time, as the hypogea saw prolonged use, the orderly placement of the earliest burials gave way to

increasingly haphazard deposition. Older burials were often piled at the bottom of the chambers to make space for new ones. The most recent burials were placed at the bottom of the pits in densely crowded instances. This presents significant challenges in definitively attributing individual grave goods.

Similar to other Phoenician cemetery areas, the placement of graves in Lilybaeum was influenced by the nature of the rock. Some areas were minimally exploited, while others were highly concentrated with burials without specific topographical orientation. However, a general north-south orientation is apparent. Phoenician burials were often disrupted by later Hellenistic and Roman graves, with new burials sometimes involving the violation or reuse of Phoenician tombs.

Analysis of grave goods reveals a wealth of ceramic vessels and a scarcity of personal ornaments. Unlike other Phoenician centers, jewelry is sporadically found in Lilybaeum. Toilet objects, including mirrors, cosmetic containers, makeup spatulas, shears, and perfume jars, are more prevalent. Pottery from the necropolis predominantly consists of traditional Phoenician forms (Benichou-Safar, 1992).

References

Bechtold, B., Valente, I. 1992. "Recenti scavi nella necropoli punica di Lilibeo. Problemi e considerazioni." In Atti delle Giornate internazionali di studi sull'area elima (Gibellina 19-22 settembre 1991), Pisa 1992, 687-701.

Bechtold, B., Frey-Kupper, S., Madella, M. 1999. La necropoli di Lilybaeum. Marsala 1999.

Benichou-Safar, H. 1992. "Nécropoles." In Dictionnaire de la Civilisation Phénicienne et Punique, edited by E. Lipinsky, 311-313. Brépols 1992.

Bisi, A.M. 1966. "Lilibeo, Marsala, ricerche archeologiche al Capo Boeo." NSc 20, 1966, 310-347.

Bisi, A.M. 1967. "Lilibeo, Marsala, ricerche archeologiche al Capo Boeo." NSc 21, 1967, 379-403.

Bisi, A.M. 1970. "Lilibeo, Marsala, scavi nella necropoli dei Cappuccini." NSc 24, 1970, 524-559.

Di Stefano, C.A. 1974. "Scoperte nella necropoli di Lilibeo." Kokalos 20, 1974, 162-171.

Di Stefano, C.A. 1984. Lilibeo. Testimonianze archeologiche dal IV secolo a.C. al V secolo d.C., Palermo 1984.

Di Stefano, C.A. 1993. Lilibeo Punica. Marsala 1993.

Lilybaeum - Human remains from the sectors of Punic Necropolis: Via Berta, Via De Gasperi, via Cicerone, Via D'Azeglio

Luca Sineo e Francesca Meli

The necropolis of Lilybaeum, also referred to as the Punic-Roman necropolis, is located beyond the protective moat, adjacent to the city's north-western and north-eastern fortifications, as evidenced by systematic archaeological investigations (Giglio Cerniglia 2012, pp. 179–206). This burial site was utilized from the city's establishment until the Imperial Age, between the 4th century BCE and the 2nd century CE (Di Stefano 1984, pp. 38–43). Significantly, the unearthing of Punic hypogea in this cemetery occurred during the period of renovation activities spanning from 1987 to 1992 (Becthold, 1999). The necropolis's primary area is predominantly situated close to the Tribunal and adjacent quarters. The samples utilized in this study were obtained from specific sectors, namely Via De Gasperi (TT. 40, 105, 114), Via Berta (TT. 99, 143, 145, 193; T. 190, as documented in Meli et al. 2023), and Via Cicerone (T. 32). The Roman period is characterized by the presence of monumental reuse, as evidenced by the discovery of the painted hypogeum of *Crispia Salvia* in 1994 along Via D'Azeglio (Meli et al., in press, 2024).

References

B. Bechtold, *La necropoli di Lilybaeum*, L'Erma di Bretschneider, 1999.

C.A. Di Stefano, *Lilibeo: Testimonianze archeologiche dal IV secolo a.C. al V secolo d.C.*, Palermo, 1984.

R. Giglio Cerniglia, *Attività della Soprintendenza BB.CC.AA. di Trapani: triennio 2007-2009, in Sicilia occidentale Studi, rassegne, ricerche,* Ampolo C., (ed.), *Atti delle settime giornate internazionali di studi sull'area e/ima e la Sicilia occidentale nel contesto mediterraneo (Erice, 12-15 ottobre 2009),Workshop «G. Nenci» diretto da Carmine Ampolo*, Pisa, Voi. II,179-206, 193-194, 2012.

Meli F., Savarino F., Romano A., Lauria G., Griffo M.G., Sineo L. – 2023. *Bio-archaeological Notes on the Punic Lilybaeum: The 190 Hypogeum*, In *Sicilia Archeologica*, 114, 69-86, L'Erma di Bretschneider, 2023.

F.Meli, R. Abbate, G. Lauria, *L'Ipogeo dipinto di Crispia Salvia. Studio antropologico delle tombe 3 e 4,* In E. Caruso e M. G. Griffo (edds.), *Lilibeo e il Mare, il Museo archeologico regionale di Marsala, in press.*

Selinunte (Sicily, Italy)

Dalit Regev, Francesca Oliveri, Pamela Toti

General Location and Chronology: Selinunte, an ancient colony of Greek Megara Iblea, was situated on the southwestern coast of Sicily. It was founded in the 7th century BC and faced destruction by the Carthaginians in 409 BC and 250 BC. Despite these setbacks, the city remained a modest center during Roman and Byzantine times. The urban layout of Selinunte was established between 580 and 570 BCE, with the sacred area positioned at the center of the acropolis. From 560 to 460 BCE, Selinunte witnessed a phase of monumentalization, marked by significant transformations of the acropolis, including the construction of temples C and D. Initially an imposing city that allied with Carthage, Selinunte formed a pact with Syracuse after the Battle of Himera in 480 BCE, distancing itself from

Carthage's protection. Unlike other Western Greek cities, Selinunte had a distinctly mercantile focus, as evident in two lagoon ports built at the sea outlet of the Modion and the Cottone, the two waterways flanking the hill to the west and east, on which arose the first settlement of the Greek city. Carthaginian rule ended during the First Punic War when the population was relocated to Lilybaeum (modern-day Marsala) as a defensive measure against Roman attacks. Carthage destroyed the city, leaving it in ruins. Subsequently, a severe earthquake in the 10th or 11th century likely further devastated the ancient monuments. The town on Manuzza Hill ended with the destruction in 409 BC. The hill later became the site of a Phoenician necropolis for the inhabitants who settled on the acropolis. This area revealed a street arrangement with main north-south roads intersecting side streets, defining various insulae. The structures in this area often featured "frame walls," a characteristic Phoenician technique (Guido and Tusa, 1987).

Excavation History: Selinunte was rediscovered by the historian Tommaso Fazello in the second half of the 16th century. The British initiated archaeological excavations in 1823. The archaeological area of Selinunte encompasses the Acropolis, the Eastern Hill, the plateau of Contrada Manuzza, the sanctuary of the Malophoros in Contrada Gaggera, and two Necropolises, namely Manicalunga and Galera Bagliazzo. The necropolis of Manicalunga and Timpone Nero, unearthed in 1871 by archaeologist F. S. Cavallari, is the largest and most affluent among Selinunte's necropolises, containing burials from the 6th and 5th centuries BC. The distance between the necropolis and the city raises doubts about whether it belonged to Selinunte or a neighboring settlement in the city's suburbs. Since 1973, an Italian-French archaeological mission has been working on the excavation and study of Selinunte's acropolis and the hill of Manuzza. While the acropolis has received significant attention, the hill of Manuzza, where the ancient city was located, has not been systematically excavated. However, evidence of an indigenous settlement, including the remains of a hut's foundation, was discovered in this area, dating back to the mid-7th century BC, coinciding with the foundation of Archaic Selinunte. An Archaic necropolis on the southeastern slopes of the hill, northeast of the acropolis, has also been established, likely associated with the initial colonial settlement (Rallo 1976/77, 1982/83, 1982).
Description of cemeteries: The necropolises of Selinunte, except for a small necropolis on the southeastern slopes of Manuzza dating back to the mid-7th century BC, are located outside the city and can be divided into three distinct areas: Buffa to the north of the eastern hill, Galera Bagliazzo 250 meters northeast of Manuzza hill, and Pipio-Bresciana and Manicalunga-Timpone Nero to the west of Gaggera hill. Almost all the dwellings unearthed are intersected by tombs from a necropolis that developed across the entire northern area of Manuzza between the second quarter and the second half of the 4th century BCE. These tombs, made from earth, slabs, capuchin, or dry-stone linings, were placed in cut stones. The necropolis emerged after the area was abandoned, with the city contracting to the acropolis hill and the edges of Manuzza closest to it. The specimens in this work seem to belong to this phase of the Manuzza necropolis.

References

Chiarenza, N. 2019. Selinunte tra la seconda metà del IV e il III secolo aC un insediamento dell’eparchia cartaginese al centro del mediterraneo. Karthago 31: 27-63

Guido, M., and Tusa, V. 1987. Guida archeologica della Sicilia. Palermo.

Jonash, M., Adorno, L., Miccichè, R. 2020 Selinunte. Sondaggio nella stratigrafia del Pianoro di Manuzza. Rapporto preliminare. https://www.fastionline.org

Rallo, A. 1976/77. "Scavi e ricerche nella città antica di Selinunte. Relazione preliminare." Kokalos XXII, 1976-1977: 720-733.

Rallo, A. 1982/83. "L'abitato di Selinunte, il quartiere punico e la sua necropoli." Kokalos XXVIII, 1982-1983: 169-174.

Rallo, A. "Selinunte: Le Ceramiche Di Vii Secolo A.C. Della Necropoli Meridionale Di Manuzza Dopo Gli Scavi 1978." Annuario della scuola archeologica di Atene e delle missioni italiane in oriente LX (1982): 203-18.

Caserma Tukory, Palermo (Sicily, Italy)

Dalit Regev, Francesca Oliveri, Pamela Toti

General Location and Chronology: The Caserma Tukory area encompasses a significant part of the extensive Phoenician necropolis, which was in use until the Roman colonization period. Stretching from Piazza Indipendenza to present-day Via Cuba and Via Pindemonte, it is bounded by Via Cappuccini and Corso Pisani. Established in 1834, the military garrison occupies the former convent of the Minim Fathers of St. Francis of Paola, known as the Rominati. The convent, dedicated to Santa Maria della Vittoria, was constructed between 1599 and 1630 on the site where a small Norman church once stood (Chirco, 2006).

Excavation history: The Phoenician necropolis in this area provides concrete and significant evidence of the early Phoenician emporium, likely founded in the 7th century BCE Tomb discoveries have occurred in this extensive necropolis since 1746, with over 700 tombs unearthed since then (Tamburello, 19677, 1974). Representative tombs are now visible in the Tuköry Barracks area along Corso Calatafimi. Excavations from 2001 to 2005 resulted in the unearthing of 72 burials within the Tuköry Barracks necropolis, including a hypogeic chamber tomb that yielded one of the oldest grave goods found in the entire necropolis, showcasing typical Phoenician forms (Spatafora, 2010a, 2010b, 2014). Combined with the discoveries made between 1989 and 1996, the total number of excavated graves reaches 150 within this strip of the necropolis. One significant discovery is a section of an earthen road, visible for approximately 30 meters, that cuts across the necropolis in a northwest-to-southeast direction. The road dates back to at least the late 6th to early 5th century BCE, as indicated by the alignment of the oldest chamber tombs along its path. The road continued to be utilized until the mid-third century BCE, during the final phase of the necropolis's use. This evidence demonstrates the existence of planned and organized funerary spaces that were integrated into the urban layout during the late archaic period. This urbanistic model, characterized by two peripheral streets running inside the city wall, parallels other Phoenician cities in Northern Africa, Sardinia and Mozia (Benichou Safar, 1986; Ramon Sainz, 1990).

Description of cemeteries: The necropolis encompasses a chronological range from the late 7th century to the early 3rd century BCE and includes various burial practices such as hypogenic chamber tombs, inhumations in lithic sarcophagi, and depositions in earthen pits or cinerary urns. Both inhumation and incineration methods were commonly employed

(Giammellaro, 2004). The earliest form of burial involved the cremation of bodies, with the resulting charred remains placed inside cineraria vessels. These vessels, their mouths sealed or covered with inverted cups, were then interred in circular or oval pits carved into the calcarenite soil. In some cases, individuals were laid supine within earthen pits and then subjected to cremation. Once the combustion process was complete, the remains, often accompanied by grave goods, were placed inside the pit as part of a specific funerary ritual. Alternatively, the corpse could be incinerated within a stone sarcophagus positioned inside a pit excavated in the calcarenite soil. Equally ancient is the practice of inhumation, where bodies were laid to rest in sarcophagi placed within pits dug into the calcarenitic rock or within hypogeic chambers. Numerous stone sarcophagi or pits, covered with calcarenite slabs or solenes, contain the remains of individuals across various age groups, with a prevalence of infants or juveniles. Grave goods found inside the sarcophagi indicate a higher social class for the deceased.

The necropolis is characterized by underground chamber tombs excavated in the calcarenitic rock. The tombs were typically accessed from the northeast and featured entrances enclosed by one or more slabs, often marked by various-shaped cippi. A staircase led to a rectangular chamber where sarcophagi were placed. These tombs were later reused, often by members of the same family, with new bodies positioned on wooden platforms supported by calcarenite slabs and covered. Some individuals were laid in simple pits or large amphorae used as burials (enchitrysmos). Personal belongings, jewelry, weapons, toiletry items, tools, and pottery were placed inside the sarcophagus, while Phoenician vessels were associated with these burials.

The coexistence of different tomb types, pit burials, and chamber burials, as well as two burial rituals, incineration, and inhumation, is evident in the Palermo necropolis. Similar burial practices can be observed in Carthage and certain settlements on the Iberian Peninsula

References

Benichou Safar, H. 1986. Les tombes puniques de Carthage. Paris.

Chirco, A. 2006. Palermo la città ritrovata. Itinerari fuori le mura. Palermo.

Giammellaro, Spanò. "I Luoghi Della Morte. Impianti Funerari Nella Sicilia Fenicia E Punica." In El Mundo Funerario. Iii Seminario Internacional Sobre Temas Fenicios, edited by A. Gonzalez Prats, 205-51. Alicante, 2004.

Ramon Sainz, M. L. 1990. Estudio sobre el ritual funerario en las necrópolis fenicias y púnicas de la Peninsula Iberica. Madrid.

Spatafora, F. 2010a. "La necropoli di Panormos." In L'ultima città. Rituali e spazi funerari nella Sicilia nord-occidentale di età arcaica e classica (Catalogo della mostra), edited by F. Spatafora and S. Vassallo, Palermo, 31-46.

Spatafora, F. 2010b. "Ritualità e simbolismo nella necropoli punica di Palermo." In Atti della Giornata di Studi in onore di Antonella Spanò (Palermo, 30 maggio 2008), Palermo, 23-39.

Spatafora, F. 2014. "Palermo: la necropoli punica (scavi 2000-2005): spazio funerario, rituali e tipologie funerarie." In Palermo: la necropoli punica (scavi 2000-2005): spazio funerario, rituali e tipologie funerarie, 445-452.

Tamburello, I. 1967. "Palermo–Necropoli: l'esplorazione 1953-1954." Notizie degli scavi di Antichità XXI: 354-378.

Tamburello, I. 1974. "Palermo. Osservazioni sulla necropoli punica." Kokalos XX, 152-161.

Palermo. Human remains from the area of the "Istituto Maria Adelaide", Palermo

Luca Sineo and Francesca Meli

Among the Punic samples from Palermo, we have to consider a few skeletal remains, presumably derived from old excavations (1914-1924) in the area of the Institute "Maria Adelaide", now included between Corso Pisani and Corso Calatafimi. The area falls within the topographic boundaries of the city's large Punic necropolis. The skeletal materials are preserved and inventoried as a historical collection at the Stebicef Department of the University of Palermo.

References

I.Tamburello, La necropoli. Rinvenimenti e storia degli scavi, in Mirabella, C. M., & Di Stefano, C. A (edds.) "Palermo punica: Museo Archeologico Regionale Antonio Salinas, 6 dicembre 1995-30 settembre 1996." (1998).

Carthage (Tunis, Tunisia)

Alfredo Coppa, Francesco La Pastina, Michaela Lucci

General Location and Chronology: The site of Carthage is located on the south-western shore of the Gulf of Tunis and corresponds to the occupied area of the modern city of Carthage (10°19′51.25 EAST - 36°51′28.83 NORTH). According to Greco-Latin sources, it is a city of Phoenician foundation that dates back to 814 BC (Moscati, 1988; Bondì, 2009). Archaeological evidence shows that the first Phoenician settlement extended from the slopes of the Byrsa hill to the coastline.

The site's planimetric layout thus places the settlement on the plain (Moscati, 1988; Bondì, 2009) and the necropolises on the hillside. Following an arrangement from east to west, we find Dermech, Douimes Juno, and Byrsa separated from the other necropolises by a corridor occupied by the Archaic settlement. The Tophet, on the other hand, is clearly isolated to the south (Gras *et al.*, 2000).

Through stratigraphic study, it was possible to descend chronologically to the first half of the 8th century BCE (Lancel et al., 1982). This study would confirm the foundation date provided by classical sources. Further evidence for the reliability of the identified chronological horizon is provided by the first 14C-calibrated dating of cattle bones found in the first layers of the Carthaginian settlement below the *Decumanus Maximus*, which yielded calibrated dates slightly earlier than 800 BCE (Docter, 2007; Docter et al., 2005, 2008).

A map of the necropolises in Carthage is depicted in Gras M., Rouliard P., Teixidor J. 2000 (see Fig. 24 page 259 there). The sites and regions of Carthage (Bir Massouda; Bordj-Djedid; Byrsa Hill; Circular Lagoon / Military Harbour; Dahar-el-Morali; Dermech; Douïmès; Hamburg Housing Quarter; Juno Hill; Odeon Hill; Rectangular Lagoon / Commercial Harbour; Sainte Monique; Tophet of Salammbô) are depicted in Bergeron M. E. 2011 (Figure 1, page 183 there).

Excavation history: The Danish consul Christian Tuxen Falbe conducted an initial investigation of the site through a study of the topography published in 1833 (Falbe, 1833).

However, Alfred Louis Delattre, in 1875, conducted the first investigation of the Punic and Roman remains (Beschaouch, 1993). Archaeological activities intensified in 1972 when UNESCO, following a request from the Tunisian authorities (concerned about the increasing urbanization of the area to the detriment of the archaeological record), promoted an international campaign to safeguard and enhance the site. The outcome of this initiative was the involvement of around ten nations in research activities and, in 1979, the inclusion of the site on the World Heritage List (Morel, 2011).

In collaboration with the Institut national d'archéologie et d'art de Tunis, which later became the Institut National du Patrimoine, it was possible to investigate a large part of the archaeological area of interest:
Circular harbour area (British excavations), *Cardo* IX (German excavations), South slope of Byrsa (French excavations), South-east slope of Byrsa (Tunisian excavations), North slope of Byrsa (Swedish excavations), Terrain Ben Ayed (German excavations), Under the *Decumanus Maximus* (German excavations), Bir Massouda/Bir Messaouda (British, Dutch and Tunisian/Belgian excavations) (Docter, 2002), Rue Ibn Chabâat (German excavations), Rue Dag Hammerskjoeld (British excavations), Rue Septime Sévère (German excavations), Magon (German excavations), Punische Seetorstraße (German excavations), Rue Sophonisbe (British excavations), *Decumanus* VI-N (Canadian excavations), 'Falbe point 90' (Danish excavations), Terrain Boudhina (Tunisian excavations) (Docter, 2007; Docter et al. , 2007), Roman Circus (excavations by the University of Georgia, USA), Yasmina (excavations by the University of Georgia, the University of Colorado and the University of Michigan, USA), (Norman and Haeckl, 1993), Antonine Baths (French and Italian excavations) (Nigro et al, 2021, 2022).

Necropolises occupy the hillside and form the city's western limit (**Supplementary Figure 1**). In the area of Dermech, close to the coastal strip, the oldest tombs, dating from the 8th to 5th century BCE, have been identified.

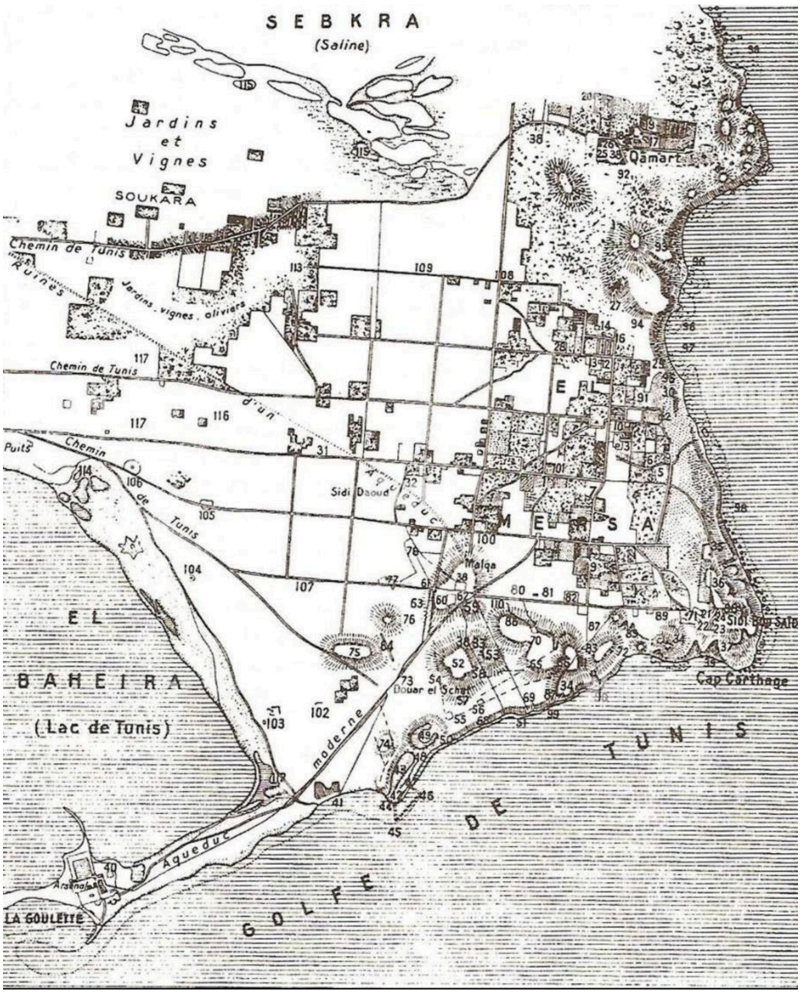

Supplementary Figure 1: **Topography of the archaeological site of Carthage** (taken from Falbe, 1833).

Description of cemeteries (see **Supplementary Table 13**):
The necropolis of **Dermech** extends from the coastal area to the slopes of the hill of Bordj-Djedid. In this area, the main burial types found in Carthage and in much of the western Punic funerary world have been identified. The oldest burials, generally dated to the end of the 8th century BCE, consist of simple rectangular pits or with the individual covered by slabs of local stone. The inhumed person was laid in a supine position (a position also maintained in the other tomb types). Burials in monolithic sarcophagi and chamber burials with a pit are also typologically present. The latter present typological variants: chamber with well, chamber with niche and well, chamber with sarcophagus and well, chamber with well and access openings, well with built chamber, and two superimposed chambers with well. The necropolis remains in use until the 4th century BCE. (Gauckler, 1915; Gras et al., 2000).

The necropolis of **Douïmès,** located south of the Dermech area, extends to the slopes of the Junone hill and was investigated along the E-W belt. Regarding burial typology, we again find burials in rectangular pits - simple, with two side niches, and with side steps - cist tomb, monolithic sarcophagus, chamber with access shaft, chamber with niche and corridor. The presence of burials with cremation in ceramic vessels is also assumed (Gaspar, 1979). The chronological horizon sees a period of use of the area as a necropolis ranging from the 7th to the 5th century BCE. (Delattre, 1897a, 1987b, 1987c, 1987d).

The hill of **Junone** is located between the necropolises of Douimes and Byrsa. The burial types include a rectangular pit with a niche in the wall, a rectangular pit covered with slabs on the inside, a monolithic sarcophagus, a small indicated chamber with an access shaft, and two small indicated chambers, one opposite the other at the bottom of the shaft. The pits are covered by slabs and are 3 and 3.5 m deep. The orientation of the burials is not certain. The site's chronology is 7th century, early 6th century BCE (Delattre, 1890, 1907, 1921; Gaspar, 1979; Merlin, 1918).

The necropolis of **Byrsa/San Luis** is located between *Decumanus* II South and *Cardo* East along the southwestern slope of the hill of the same name. There are burials in rectangular pits, in double rectangular pits, in pits with two lateral support steps at the bottom, in pits with amphorae for the burial of children, in rectangular cists, in chambers with a well, in chambers with a sarcophagus, niches and a well, and in chambers with niches and a corridor. Again, the orientation does not seem to follow any precise rules. The chronology determined on the basis of pottery is between the 6th and 4th centuries BCE. (Delattre, 1890, 1896; Gaspar, 1979; Gras et al., 2000; Lancel et al., 1979, 1982).
On Byrsa Hill, near the entrance to the National Museum of Carthage, a Punic burial crypt was discovered in 1994 with the remains of a young man with grave goods, dated to the late 6th century BCE, who had rare European mitochondrial haplogroup (U5b2c1) linking his maternal ancestry to Phoenician-influenced localities somewhere on the northern coast of the Mediterranean, the Mediterranean islands or the Iberian Peninsula (Matisoo-Smith, et al, 2016).

Another necropolis stands in the **Rabs** area, from which it takes its name. The burial types found here are a chamber with a pit, a chamber with a bench and excavated pit, two side chambers, one at the bottom and the other in the middle of the pit, and three overlapping chambers with a pit. They are among the burials with the greatest excavation depth, which is probably consequential to the thick layer of clay soil covering the rocky base. Again, it is not

possible to determine the orientation of the burials. As far as the period of use of the necropolis is concerned, this is between the 4th and 3rd century BCE. (Delattre, 1906; Gaspar, 1979).

The necropolis of **Ard-et-Touibi** is located in the area immediately west of the Antonine baths. The tombs are located at the northern end of a rocky platform on the esplanade that extends west of the Antonine baths. The characteristics types are: rectangular tomb, rectangular tomb with recess inside, a chamber with the well, chamber with niche and well, bed with sarcophagus and well, two chambers with well, and two beds with sarcophagus and well. Most burials are dated to the 4th century BC (Gaspar, 1979; Poissont and Lantier, 1927).

The **Bordj-Djedid** necropolis is located on the hill of the same name. The burials were excavated in the tuff. The most characteristic types are the rectangular pit, the simple pit, the chamber with pit, the chamber with pit and pit, and the chamber with corridor. The chronology of the necropolis ranges from the 5th to the 2nd century BCE (Delattre, 1899b, 1890, 1908; Gaspar, 1979; Gauckler, 1915 a, 2015 b).

The necropolis of **D'ard-el-Kheraib** is located in the western part of Bordj-Djedid. The characteristic burial types are: single pit tombs, with a side chamber in the center of the pit, a side chamber with a pit, a chamber with a sarcophagus and a pit, two chambers with a pit, two side chambers at the base of a pit, and three chambers with an access shaft. Most of the burials are oriented NE-SW, although the orientation is not respected in all cases. It covers a time span from the 5th century to the end of the 4th century BCE. (Gaspar, 1979; Merlin and Drappier, 1909).

The necropolis of **Ard-el-Morali** is located in the lower part of the hill of the same name. The most relevant burial type is the two-chamber tomb with an access shaft. The chronology dates back to the 4th century BCE (Gaspar, 1979; Merlin, 1918).

The **Bou-Mnijel** necropolis is also located on the Bordj-Djedid hill, west of the fort and north of the large cisterns. The most significant types are rectangular tombs and chamber tombs with shafts. They are placed chronologically in the second half of the 4th century BCE (Gaspar, 1979; Merlin, 1918).

The necropolis of the **Theatre** consists of a series of tombs located in the upper part of the hill. The burial typology consists of a single model, namely chamber tombs with a pit. The chambers and pits are dug into the rock, closed in some cases by a slab. Chronologically, they date back to the 4th century BCE (Drappier, 1911; Gaspar, 1979; Nigro et al., 2022).

The **Santa Monica** necropolis is located to the N-NE of the Bordj-Djedid hill. The graves are of the pit, chamber with pit, chamber with one or two pits and pit, chamber with sarcophagus and pit, two-chamber with bench, pit and pit, three- and four-chamber with pit, chamber with pits and access corridor, and chamber with pits and bench with corridor type. The necropolis remains in use from the 4th to the 2nd century BCE (Delattre, 1899a, 1899b, 1902, 1903, 1905; Gaspar, 1979; Gauckler, 1915 a, 2015 b).

The skeletons analyzed in the present study should all have come from the French mission's excavations at Birsa Hill in the late 1970s (Lancel et al., 1982), and the calibrated absolute chronologies carried out on these materials also support this attribution (**Supplementary Table 14**).

Supplementary Table 13: Chronology and Reference list of Carthage cemeteries.

NECROPOLIS	CHRONOLOGY	REFERENCES
**Dermech**	IV century BCE	Gauckler, 1915; Gras et al., 2000
**El-Alia**		Gauckler, 1897; Novak, 1895
**Douïmès**	VII - V century BCE	Delattre, 1897a, 1987b, 1987c, 1987d
**Junone**	VII century, beginning of VI century BCE	Delattre, 1890, 1907, 1921; Gaspar, 1979; Merlin, 1918
**Byrsa/San Luis**	VI - IV sec. BCE	Delattre, 1890, 1896; Gaspar, 1979; Gras et al., 2000; Lancel et al., 1979, 1982
**Rabs**	IV - III century BCE	Delattre, 1906; Gaspar, 1979
**Ard-et-Touibi**	IV century BCE	Gaspar, 1979; Poissont and Lantier, 1927
**Bordj-Djedid**	V - II century BCE	Delattre, 1899b, 1890, 1908; Gaspar, 1979; Gauckler, 1915 a, 2015 b
**D'ard-el-Kheraib**	End of IV century BCE	Gaspar, 1979; Merlin and Drappier, 1909
**Ard-el-Morali**	IV century BCE	Gaspar, 1979; Merlin, 1918
**Bou-Mnijel**	Half of IV century BCE	Gaspar, 1979; Merlin, 1918
**Teatro**	IV century BCE	Drappier, 1911; Gaspar, 1979; Nigro et al., 2022
**Santa Monica**	IV-II century BCE	Delattre, 1899a, 1899b, 1902, 1903, 1905; Gaspar, 1979; Gauckler, 1915 a, 2015 b

Supplementary Table 14: Overview of individuals excavated from Carthage and analyzed in this study (see also Supplementary Tables 3-4).

Site	Burial Code	Date	mt haplogroup	sex	Assessment
Carthage	2	800-300 BCE	H1bo	F	Pass
Carthage	3	800-300 BCE	H5a	F	Questionable
Carthage	15	373-199 calBCE (2215±20 BP, PSUAMS-11780); 370-197 calCE (2210±20 BP, PSUAMS-11007)	K1a+195	F	Pass
Carthage	25	450-150 BCE	H2a2	F	Pass
Carthage	27	800-300 BCE	H1e7	M	Pass
Carthage	28	409-365 calCE (2315±20 BP, PSUAMS-11008)	P	M	Pass
Carthage	33	800-300 BCE	H5a	M	Pass
Carthage	36	800-300 BCE	K1a	F	Pass
Carthage	40	800-300 BCE	H3	F	Pass
Carthage	41	800-300 BCE	JT	M	Questionable
Carthage	42	800-300 BCE	K1a4	M	Pass
Carthage	43	800-300 BCE	H	M	Pass
Carthage	44	800-300 BCE	V15	M	Pass
Carthage	62	800-300 BCE	H1e1	M	Questionable
Carthage	63	800-300 BCE	K1	M	Questionable
Carthage	64	800-300 BCE	H+195	F	Pass
Carthage	72	800-300 BCE	H2a2	U	Questionable
Carthage	71	409-365 calBCE (2315±20 BP, PSUAMS-11033)	U4b1b1	M	Pass
Carthage	73	800-300 BCE	T2b3	M	Pass
Carthage	81	800-300 BCE	K1a30	M	Pass
Carthage	87	800-300 BCE			
Carthage	88	800-300 BCE	T2b37	M	Pass
Carthage	89	800-300 BCE	U6a7c1	F	Pass
Carthage	91	800-300 BCE	T2g1	F	Pass
Carthage	102	450-150 BCE	R	F	Pass
Carthage	Amphora Burial	500-300 BCE	J2a2d	M	Pass

References

Bergeron M. E. (2011) Death, gender, and sea shells in Carthage, Pallas Revue d'Études Antiques, 86, pp. 169-189. https://doi.org/10.4000/pallas.2143

Beschaouch, A. (1993), *La légende de Carthage*, éd. Découvertes Gallimard, Paris.

Bondì S.F., Botto S.F., Garbati G., Oggiano I., 2009. *Fenici e Cartaginesi. Una civiltà mediterranea*, Ist. Poligrafico dello Stato, Roma.

Delattre, A.L. (1890) *Les tombeaux puniques de Carthage*, Imprimerie Mougin-Rusand, Lyon.

Delattre, A.L. (1896) *La necropole punique de la colline de Saint-Louis*, Imprimerie Mougin-Rusand, Lyon.

Delattre, A.L. (1997a) *Carthage. La nécropole punique de Douïmès. Fouilles de 1893-1894*, Imprimerie E. Petithenry, Paris.

Delattre, A.L. (1897b) La nécropole punique de Douïmès à Carthage. Fouilles de 1895-1896, Mémoires de la Société Nationale des Antiquaires de France, LVI, pp. 255-395).

Delattre, A.L. (1897c) Un mois de fouilles dans la nécropole punique de Douïmès à Carthage (Fév. 1895), Rev. Tun., IV, pp. 170-177.

Delattre, A.L. (1897d) *Carthage. Quelques tombeaux de la nécropole punique de Douïmès (1892-1894)*, Imprimerie Mougin-Rusand, Lyon.

Delattre, A.L. (1899a) *Carthage. La nécropole punique voisine de la Colline de Sainte Monique*, Imprimerie E. Petithenry, Paris.

Delattre, A. L. (1899b) Fouilles exécutées á Carthage, pendant le premier trimestre de 1899, dans la necropole punique située entre Bordj-Djedid et la colline de Saint Monique. Comptes rendus des séances de l'Académie des Inscriptions et Belles-Lettres, 43e année, N. 3, pp. 308-322.

Delattre, A.L. (1902) Rapport sur les fouilles de la nécropole punique voisine de Sainte-Monique. Découverte d'un cinquième sarcophage de marbre blanc, Comptes rendus des séances de l'Académie des Inscriptions et Belles-Lettres, 44e année, N. 4, pp. 443-450.

Delattre, A.L. (1903) Carthage. Nécropole punique voisine de Sainte-Monique. Deux sarcophages anthropoïdes en marbre blanc, Comptes rendus des séances de l'Académie des Inscriptions et Belles-Lettres, 47e année, N. 1, pp. 23-33.

Delattre, A.L. (1905) La necropole voisine de Saint Monique. Groupe de figurines, Comptes rendus des séances de l'Académie des Inscriptions et Belles-Lettres, 49e année, N. 1, pp. 125-134.

Delattre, A. L. (1906) *La nécropole des Rabs Prêtres et Prêtresses de Carthage - Deuxième année des fouilles*, Imprimerie Paul Feron-Vrau, pp 5-42.

Delattre, A. L. (1907). Fouilles de Carthage, Bulletin Archéologique du Comité des Travaux Historiques B.A.C., pp. 443-447.

Delattre, A. L. (1908). Fouilles dans le flare Sud de la colline de Bordj-Djedid a Carthage. Comptes rendus des séances de l'Academie des Inscriptions et Belles Lettres, 52e année, N. 9, pp. 592-601.

Delattre, A. L. (1921) Tombeaux puniques de la colline de Junon à Carthage (1920-1921), Comptes rendus des séances de l'Académie des Inscriptions et Belles-Lettres 65e année, N. 2, pp. 95-100.

Docter, R.F. (2002) Catthage Bir Massouda: Excavations by the Universiteit van Amsterdam (UVA) in 2000 abd 2001 (1), CEDAC, Carthage, 21, pp. 29-34. DOI: 10.2143.BAB.78.0.503921.

Docter R.F. (2007). Published Settlement Contexts of Punic Carthage, Carthage Studies, 1, 2007, pp. 37-76.

Docter, R. F., Niemeyer, H. G., Nijboer, A. J., Van Der Plicht, J. (2005) Radiocarbon Dates of animal bones in the earliest levels of Carthage. In Oriente e Occidente: metodi e discipline a confronto. Riflessioni sulla cronologia dell'età del ferro in Italia. Edited by G. Bartoloni, F. Delpino, R. De Marinis, P. Gastaldi (eds.), Mediterranea. Quaderni Annuali dell´Istituto di Studi sulle Civilta Italiche e del Mediterraneo Antico del Consiglio Nazionale delle Ricerche, pp. 557-577.

Docter R.F., Chelbi, F, Maraoni Telmini, B., Niemeyer, H. G., De Wulf, A. (2007) Punic Carthage: Two decades of archaeological investigations. In: José Luis López Castro (ed.), *Las ciudades fenicio–púnicas en el Mediterráneo Occidental*, Editorial Universidad de Almería, pp. 85-104.

Docter R.F., Chelbi, F., Maraoui Telmini, B., Nijboer, A. J., Van Der Plicht, J., Van Neer, W., Mansel, K., Garsallah, S. (2008) New radiocarbon dates from Carthage: Bridging the gap between history and archaeology? in C. Sagona (ed.), Beyond the Homeland. Markers in Phoenician Chronology, Ancient Near Eastern Studies, Supplement Series 28, Peeters Press, Leuven, pp. 379-422.

Drappier, L., (1911): La necropole punique du Theatre de Carthage, Revue Tunisienne, 1911, pp. 3-27.

Falbe C. T. (1833) *Recherches sur l'emplacement de Carthage*, Impr. Royale, Paris.

Gaspar, A. T. 1979 Las tumbas fenicias y púnicas del Mediterráneo occidental: (estudio tipológico), Servicio de Publicaciones de la Universidad, Sevilla 1979.

Gauckler P. (1915a) *Nécropoles Puniques de Carthage, I*, Paris.

Gauckler P. (1915b) *Nécropoles Puniques de Carthage, II*, Paris.

Gras, M., Rouliard, P., Teixidor, J. (2000) *L'Universo Fenicio*, Giulio Einaudi editore, Torino.

Lancel S., Carrié J.-M., Deneauve, Pierre Gros, J., Sanviti, N., Thuillier, J.-P., Villedieu, F., Saumagne, C. (1979) Mission archéologique française à Carthage. Byrsa I. Rapports préliminaires des fouilles (1974-1976), Collezione de l'école Français de Rome, Roma.

Lancel. S, Morel J.P., Thuillier J.P. (1982) Mission archeologique française a Carthage, Birsa II Rapport preliminaires sur les fouilles 1977-1978: niveaux et vestiges puniques, Collezione de l'école Français de Rome 41, Roma.

Matisoo-Smith, E.A., Gosling, A.L., Boocock, J., Kardailsky, O., Kurumilian, Y., Roudesli-Chebbi, S., Badre, L., Morel, J-P., Sebaï, L.L., Zalloua, P.A. (2016) A European Mitochondrial Haplotype Identified in Ancient Phoenician Remains from Carthage, North Africa, PLoS ONE, 11(5): e0155046. doi:10.1371/journal.pone.0155046

Merlin, A. (1918) Fouilles de tombeaux puniques a Carthago. Tombeaux de la colline de Junon, Bulletin Archéologique du Comité B.A.C. pp. 218-334.fanta

Merlin A., Drappier L. (1909) La nécropole punique d'Ard el Khéraïb, Notes et Documents des antiquites et des Arts, III, pp. 5-61.

Morel, J.-P. (2011). Mission archéologique de Carthage-Byrsa. Les Nouvelles De l'Archéologie, 123, pp. 39–43. https://doi.org/10.4000/nda.1472.

Moscati, S. (1988) *I Fenici*, Bompiani, Milano.

Nigro, L., Cappella, F., Coppa, A., Genchi, F., Fantar, M., & Achour, M. (2021). Cartagine: la città e le necropoli rapporto preliminare sulla prima campagna di scavi (2021) dell'Institut National du Patrimoine e dell'Università di Roma «La Sapienza». Vicino Oriente, 25, 1. https://doi.org/10.53131/VO2724-587X2021_1

Nigro, L., Cappella, F., Achour, M., & Fantar, M. Nuovi scavi a Cartagine. rapporto preliminare sulla seconda campagna di scavi (2022) dell'Institut National du Patrimoine e dell'Università di Roma «La Sapienza». Vicino Oriente, 26, 135–162. https://doi.org/10.53131/VO2724-587X2022_8

Norman, N. J., & Haeckl, A. E. (1993). The Yasmina necropolis at Carthage, 1992. Journal of Roman Archaeology, 6, pp. 238–250. https://doi.org/10.1017/S1047759400011570

Poinssot, L. - Lantier, R. (1927) Fouilles à Carthage: Bulletin Archéologique du Comité des Travaux Historiques B.A.C., pp. 437-474.

Kerkouane (Cap Bon, Tunisia)

Alfredo Coppa, Francesco La Pastina, Michaela Lucci

General Location and Chronology: The city located on the eastern side of Cap Bon was founded in the sixth century BCE and is currently one of the most known Punic settlements (Fantar M.H., 1984, 1987, 1988, 2000; Bondì et al. 2009; Miles, 2010).

A plan of the Necropolis of Kerkouane (Arg El-Ghazouani area) is depicted in Fantar M., 2002 Recherches sur l'architecture funéraire punique du Cap Bon (see Fig. 3., page 59 there). See aerial photo in **Supplementary Figure 2**.

Excavation history: Thanks to the research launched in 1953 to ensure its recovery. The absence of modern structures made it possible to investigate the city facilities as they must have looked when abandoned, namely in the third century BC. Regarding the funerary areas, the city boasted a system of four Necropolises, all extra-urban as usual. The most important sector is Arg El-Ghazouani, northwest of the city, the only one systematically investigated and still being excavated (Acquaro et al., 1973; Faster M., 2002, 2003).

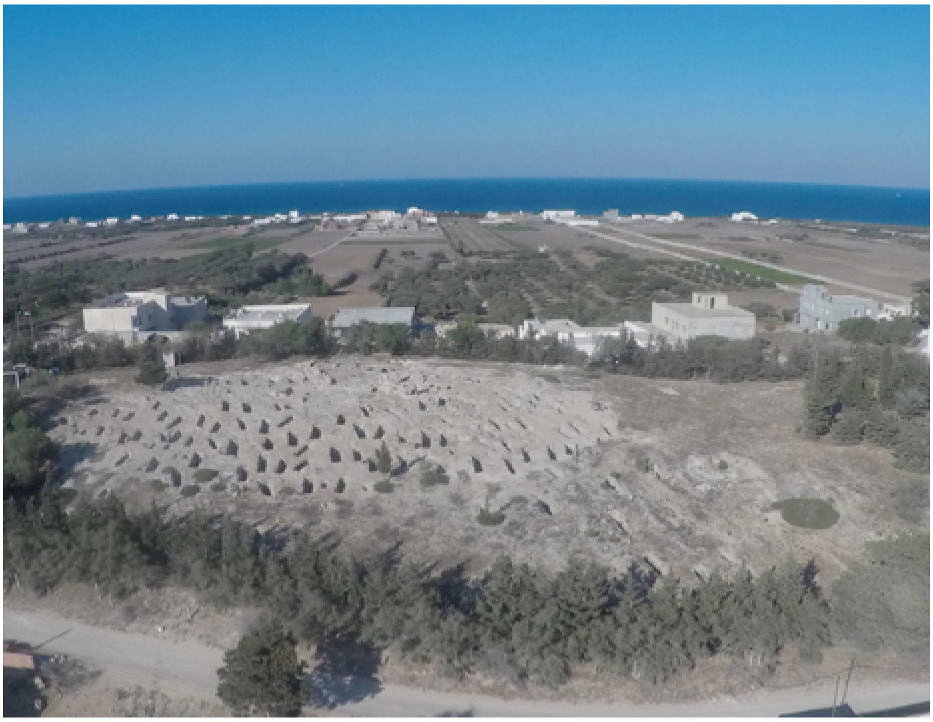

Supplementary **Figure 2: Aerial drone photo of the Necropolis of Kerkouane (Arg El-Ghazouani area).** Aerial drone photo taken by Prof. Raimondo Zucca.

Description of cemeteries: The necropolis includes tombs dating from the sixth to the third century and is located on a hill overlooking the sea on the east and northeast sides, in the tombs, dug directly into the rocky bank, the burial ritual is prevalent. About fifty grave burials and 150 specimens of the single-chamber type are known, with access via dromos. The latter occupies the entire corridor width; however, it is not uncommon for the steps to be set on both sides of the entrance or a single side. There is no shortage of cases where the stairs occupy the entire corridor and narrow downwards (Fantar M., 2002, 2003). The first article

on the genomics of some individuals from the necropolis was recently published (Moots et al., 2023).

Supplementary Table 15: Overview of 11 individuals whose genomes were published by Moots et al. (2023).

Kerkouane	Tomb 1/97 Ind. D	735-413 calBCE (2435±15 BP, UCIAMS-235940)
Kerkouane	Tomb 2/00 Cranio 4B	658-407 calBCE (2415±15 BP, UCIAMS-237819)
Kerkouane	Tomb 2/06 Ind. A	763-542 calBCE (2485±15 BP, UCIAMS-235908)
Kerkouane	Tomb 4/87 Ind. C	734-408 calBCE (2425±20 BP, PSUAMS-11779)
Kerkouane	Tomb 4/87 Ind. D	750-416 calBCE (2450±15 BP, UCIAMS-237820)
Kerkouane	Tomb 5/08	515-392 calBCE (2370±20 BP, PSUAMS-11778)
Kerkouane	Tomb 6 Ind. 2	723-404 calBCE (2415±20 BP, PSUAMS-11775)
Kerkouane	Tomb 6 Skeleton 2	717-404 calBCE (2410±20 BP, PSUAMS-11776)
Kerkouane	Tomb 15/C2 Ind. A	364-197 calBCE (2205±20 BP, PSUAMS-11774)
Kerkouane	Tomb 15/C2 Ind. 2	538-399 calBCE (2390±20 BP, PSUAMS-11777)
Kerkouane	Tomb 15/C	744-413 calBCE (2440±15 BP, UCIAMS-237821)

References

Acquaro E., Bartoloni P., Ciasca A., Fantar M.H., 1973 Prospezione Archeologica al Capo Bon, I collezione di studi fenici 2, Roma: Consiglio nazionale delle ricerche: 1-80

Bondì, S.F., Botto M., Garbati G., Oggiano I., 2009. “ Fenici E Cartaginiesi, Una Civiltà Mediterranea”, Libreria Dello Stato, Istituto Poligrafico E Zecca Dello Stato.

Fantar M.H., 1984 Kerkouane, cité punique du cap Bon (Tunisie), I, Cadre géographique et historique. La découverte, Tunis.

Fantar, M.H., 1988. “Le Cavalier Marin de Kerkouane.” Africa, Quaderni 1: 19–32.

Fantar M.H., 1987, Kerkouane, une cité punique au Cap Bon, Tunis, Maison tunisienne de l’édition.

Fantar, M. H., 2000, L’urbanisme et l’architecture puniques: le cas de Kerkouane, Fenicios y Territorio actas del II Seminario Internacional sobre Temas Fenicios, A. González Prats, ed., Diputación Provincial de Alicante e Instituto Alicantino de Cultura Juan Gil-Albert, Alicante: 71-83.

Fantar M., 2002 Recherches sur l’architecture funéraire punique du Cap Bon, Roma 2002

Fantar, M. 2003. “Espaces Culturels à Kerkouane.” Comptes Rendus Des Séances de l’Académie Des Inscriptions et Belles-Lettres 147 (2): 817–24.

Miles, R., 2010. Carthage Must Be Destroyed: The Rise and Fall of an Ancient Mediterranean Civilization. London: Allen Lane.

Moots, Hannah M., Margaret Antonio, Susanna Sawyer, Jeffrey P. Spence, Victoria Oberreiter, Clemens L. Weiß, Michaela Lucci, et al., A Genetic History of Continuity and Mobility in the Iron Age Central Mediterranean. *Nature Ecology & Evolution*, August. https://doi.org/10.1038/s41559-023-02143-4..

Khenkela Cave (Constantine, Algeria)

Alfredo Coppa and Michaela Lucci

General Location and Chronology:

Khenkela is related to the Chabor Cave burial ground located one kilometer from Khenchela, at the edge of Aurès, in the foothills of the Amamra Mountains (07°08’42’’ EAST - 35°25’39’’ NORTH). The necropolis had been initially attributed to the Neolithic period, but the C14 dating of the only burial that yielded a positive ancient DNA result was calibrated to 816-791 calBCE (2635±15 BP, PSUAMS-5278), which places the necropolis in the Early Iron Age.

Excavation history:

The site was discovered in 1876 by M. Jullien who was a military lieutenant of the 3rd Rifle Regiment with a passion for archaeology, probably stationed in the area. During excursions, he had first identified a series of mounds, one of which he excavated but which yielded extremely fragmentary remains, and then a series of burial caves excavated in the Chabor Mountains. One of these caves was excavated and yielded the remains of some twenty-five skeletons, seven more or less complete skulls, traces of a hearth, three fragments of coarse flint, pottery, and animal bones, but no traces of metal, from which the Neolithic attribution of the cave probably derives.

Description of cemeteries:

The description we have of the find indicates that the bodies were placed at the bottom of a cave and then covered with stones, but it is not clear whether the burial took place at the same time or at later times. The skeletons are referable to 25 individuals, and seven more or less complete skulls were found, possibly also referable to the 25 individuals. In the catalog of the Musée de l'Homme, 16 inventory numbers are attributed to the locality of Khenchela, of which 11 skulls (6 skulls and 5 calvaria) are all gifts from Jullien, but only 7 are specified as coming from the Chabor Cave, but only for 5 of them is it specified that they are Neolithic skulls. The remaining inventory numbers refer to femurs and tibiae from the Chabor Cave.
The skull that yielded positive results, inventory number 6150, both by the sequence of the inventory number and the date, seems to be part of this same group of skulls, although the other 7 seem to be those referable to Jullien in his two articles (Jullien, 1876, 1877), which are the same article presented as a letter to the "Société d'Anthropologie de Paris" Meeting of 6 April 1876 and article in 1877. The seven complete skulls gave the following average results: dolichocephalic type, developed progratism, sphenoidal angle 136°.

References

Jullien, M. 1876. *Sur les gisements de silex et les tombeaux mégalitiques de Khenchela*. Bulletins de la Société d'anthropologie de Paris, II° Série. Tome 11, Séance du 6 avril 1876: 162-165.

Jullien, M. 1877. *Découvertes en Algérie de silex taillés et de dolmens*. Matériaux pour l'Histoire Primitive et Naturelle de l'Homme, Revue Mensuelle Illustrée. Douzième Volume, 2e Série. – Tome VIII: 144-146.

Section 2 - Dataset description

After screening bone material from 398 individuals (**Supplementary Table 1-2**), we obtained genome-wide data meeting standards for ancient DNA authenticity for 210 unique individuals (**Supplementary Table 3**), 196 of which were sampled from Phoenician or Punic sites, and the remaining 14 were sampled from other relevant archaeological contexts. One Iron Age individual sampled from inland Algeria (Khenkela) was used in the analysis to model North African ancestry. Seven individuals were sampled from a Bronze Age archaeological context in the Phoenician/Punic site of Motya in Sicily and were used as part of our model for pre-Phoenician Sicilian ancestry. Six individuals sampled from Monte Falcone, Sicily, were combined with previously published individuals from the same site (Reitsema et al. 2022) and used to model indigenous Sicilian ancestry in the Iron Age (**Extended Data Figure 9a**). The remaining 196 individuals were sampled from 14 Phoenician and Punic settlements along the Mediterranean coast (see **Figure 1a** for geographic locations and **Supplementary Table 16** below for a summary). Ten individuals from the Punic site of Kerkouane in North Africa, for which we generated in-solution enrichment data, had been shotgun-sequenced independently by a recently published study (Moots et al. 2023). In our study, we analyzed the newly generated sequences for these individuals (as well as 17 new genomes from additional individuals from the same site), which had substantially higher sequencing coverage on average and, importantly, were generated using the same technical methodology as other samples in our dataset, thus reducing concerns about bias due to different data generation methods. For 174 of the 210 individuals in our dataset, we obtained data from more than 20,000 SNPs; the remaining 36 were not used in our genome-wide analysis due to the sparsity of their data.

Because several of the sampled Punic sites were occupied by the Romans starting from the mid-3rd century BCE (Sardinia, Sicily) and 2nd century BCE (North Africa, Iberia), it was important for us to obtain direct radiocarbon dates on many of the skeletal samples we analyzed for ancient DNA to confidently associate sampled individuals with Phoenician or Punic contexts. We thus generated 111 direct accelerator mass spectrometry-based dates on bone for 99 of these individuals (**Supplementary Table 4**). For eleven individuals, we had bones analyzed by two different labs, with the two date ranges obtained from the different labs being highly concordant in all cases. For these individuals, we used the R_combine method to combine date ranges. For 14 additional individuals, we obtained direct radiocarbon dates reported in other sources. Thus, overall, we had direct radiocarbon dates for 113 individuals, out of which 106 were sampled from Phoenician and Punic sites (three of which had fewer than 20,000 SNPs and were thus not analyzed). Three additional individuals who were not directly dated (two from Villaricos and one from Lilybaeum) were inferred to be biologically close relatives of directly dated individuals, and we associated them with the same time range.

For 20 individuals sampled from Punic sites, radiocarbon dates suggest dating to the time period of Roman hegemony. The majority of these individuals (15) were from various sites in Sicily, and the remainder were from Tharros (3), Villaricos (1), and Cadiz (1). We analyzed and discussed these samples separately (**Extended Data Figure 1** and **Extended Data Figure 9**). Because of the high rate of samples from Sicilian Punic sites whose estimated radiocarbon dates post-dated the Roman-Punic wars, we excluded 15 samples from Sicily that did not have direct radiocarbon dates (and had sufficient sequencing

coverage). In contrast, the individuals sampled from Akhziv, Kerkouane, and Carthage were assumed to be Phoenician or Punic even if they were not directly dated because all radiocarbon dates from those sites were consistent with a Phoenician or Punic context and strong archeological associations (see **Archaeological Site Descriptions**). In particular, Kerkouane was abandoned in the mid-third century BCE, and Carthage was destroyed in the mid-second century BCE. The cemeteries we have samples from were not reused by later Roman settlers. The 13 individuals from Iberia and Sardinida without radiocarbon dates were analyzed as part of a group of samples with somewhat reduced confidence in a Punic context (see below).

Interpreting radiocarbon dates of the first millennium BCE is complicated by two prominent plateaus in the radiocarbon calibration curve: the so-called Hallstatt plateau 800-400 BCE and a subsequent, shorter plateau 400-200 BCE (**Supplementary Figure 3**). We thus partitioned samples into four groups based on their calibrated date ranges. We grouped all samples whose calibrated date range was from 900 BCE - 360 BCE, which spans the Hallstatt plateau. We similarly grouped samples whose date ranges were contained in the range 450 BCE - 170 BCE (late Punic period) and 200 BCE - 600 CE (Roman period). The boundaries of these ranges extend beyond the plateaus of the calibration curve to ensure that every sample fully fits into one of the groups. Three individuals were radiocarbon dated to 450 BCE - 360 BCE and could thus be associated with either of the two early time ranges. We associated all three individuals with the latter. Ten individuals had broad date ranges that overlapped the two later date ranges, so we associated them with a fourth group (400 BCE - 50 BCE). We note that individuals in this group may be associated with a Punic context but might have also been influenced by Roman expansion. We thus consider these individuals separately in the analysis, together with the 13 individuals from Iberia and Sardinia without radiocarbon dates (see above).

Supplementary Table 16 below summarizes the 196 newly sequenced individuals sampled from Phoenician and Punic sites (excluding the seven individuals sampled from a Bronze Age archaeological context in Motya). A dark gray background indicates high confidence in the archaeological context (108 individuals), whereas light gray indicates somewhat reduced confidence due to uncertainty in the sample date (23). The blue background indicates individuals associated with the subsequent Roman period (20 individuals total). Individuals with a white background were omitted from our genetic analysis due to low coverage (28 genomes) or low confidence in their Punic context (17 genomes). In our analysis, we also considered whole genome data from nine previously published individuals sampled from a Punic context in Ibiza (native name: Eivissa) (P. Zalloua et al. 2018) and Villamar and Monte Sirai in Sardinia (Marcus et al. 2020). The individual from Ibiza and the six individuals from Villamar were all dated to a time range in 450 BCE - 170 BCE. One individual from Monte Sirai was dated to a time range contained in 800 BCE - 400 BCE, and another did not have a radiocarbon date. Thus, our analyzed Phoenician/Punic dataset had a total of 116 individuals with high confidence in the archaeological context (108 newly sequenced and eight previously published) and 24 individuals with somewhat reduced confidence due to uncertainty in the sample date (23 newly sequenced and one previously published). To these, we added 26 newly sequenced individuals from other relevant contexts: 20 individuals associated with the Roman period, one Iron Age individual sampled from inland Algeria (Khenkela), and five Bronze Age individuals from Motya, Sicily.

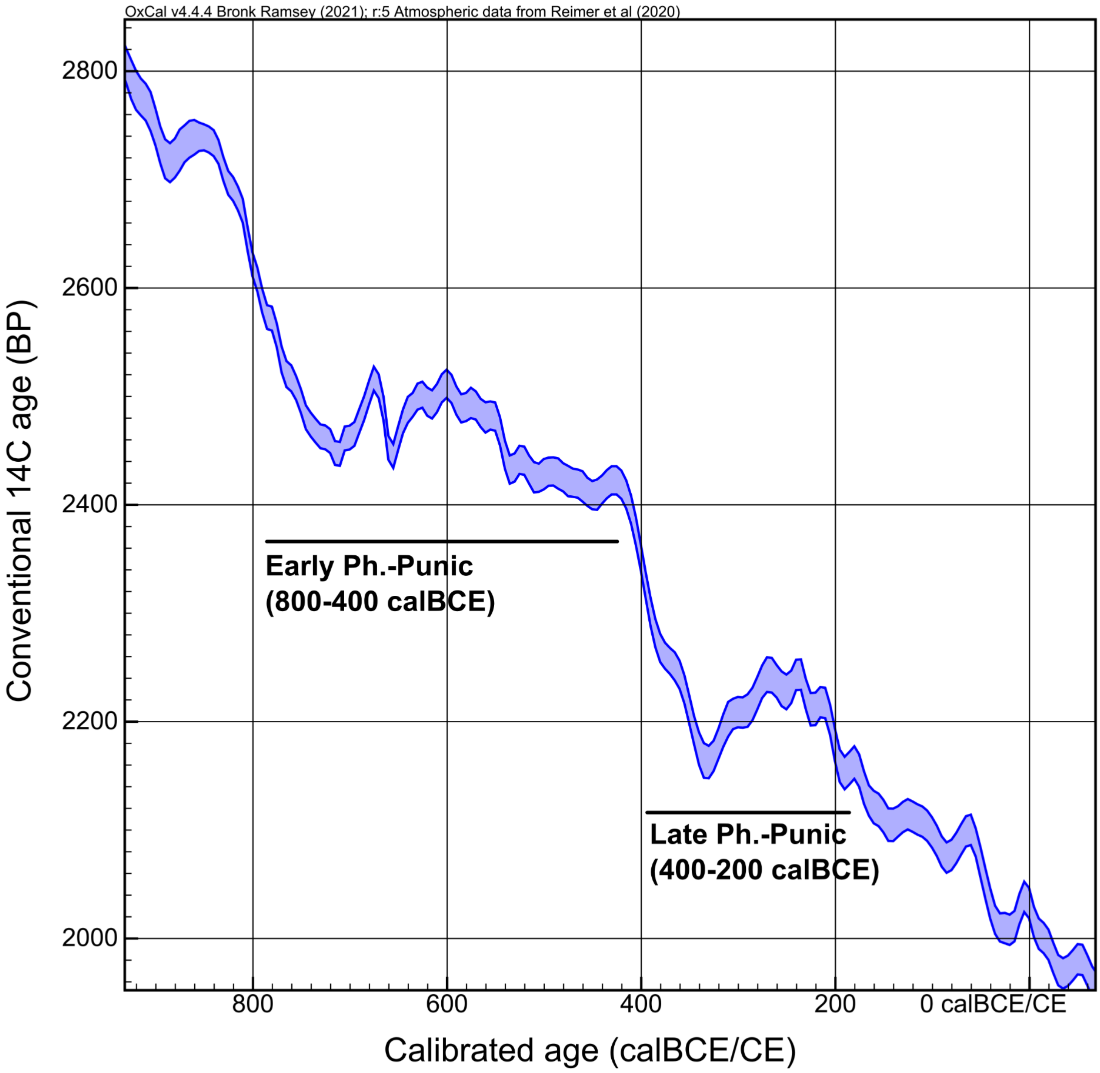

Supplementary Figure 3: Radiocarbon calibration curve of the first millennium BCE, depicting the two main time ranges we used. Modified from OxCal v.4.4.4. (Ramsey 2009) showing the IntCal20 calibration curve (Ramsey 2009; Reimer et al. 2020). A raw ^{14}C age (on the Y-axis, measured in BP) is translated to a calibrated age (calBCE/calCE, on the X-axis). The calibration curve has a plateau from 800-400 BCE (the “Hallstatt Plateau”) followed by a shorter plateau from 400-200 BCE. We used these two plateaus to group samples in our data set.

Supplementary Table 16: Period categories for 196 individuals sampled from Phoenician and Punic sites.

Region	Site (total samples)	> 20,000 SNPs. ^{14}C date range*:					< 20,000 SNPs
		900 BCE - 360 BCE	450 BCE - 170 BCE	400 BCE - 50 BCE	200 BCE - 600 CE	No ^{14}C date	
Levant	Akhziv (16)	2				11†	3
North Africa	Kerkouane (28(1))	15	3 (2)			9†	1
	Carthage (21)		5 (2)			12†	4
Sicily	Motya (12)	7		1		3	1
	Birgi (12)	7	1	1	3		
	Lilybaeum (33)		2	5	11 (3)	11	4
	Selinunte (9)	2	5			1	1
	Palermo (3)		1		1		1
Sardinia	Tharros (22	6	4	3	3	4	2
Iberia	Cadiz (5)	1	1		1		2
	Malaga (8)		3			4	1
	Villaricos (12)		7 (4)		1	2 (5) / 1	1
	Ibiza (12)		4			3	5
	Granada (3)					1 (6)	2
Total	196	40	36	10	20	62	28

* 95% confidence interval calibrated date range. The boundaries of the four date ranges were set to ensure that every sample fits into one of the groups (see text).

† Added to high-confidence Phoenician-Punic context (based on consistent ^{14}C dates from the site)

(1) Ten individuals from Kerkouane were separately sequenced by (Moots et al. 2023).

(2) Two individuals from Kerkouane and one from Carthage were radiocarbon-dated between 450 BCE and 360 BCE, which falls within the overlapping period of two time ranges. We associated them with the later time range.

(3) Two individuals from Lilybaeum were inferred to be 3rd-degree relatives based on sharing long genomic segments. One was radiocarbon dated to the Roman period, so we also associated the second individual with the same time range.

(4) Five individuals from Villaricos tomb 774 were inferred to be biologically closely related based on the sharing of long genomic segments and pairwise genetic diversity (Figure 5). Two individuals are radiocarbon dated to ca. 400-200 calBCE and one to 752-416 BCE. We associated all five individuals with the 450-170 BCE time interval.

(5) We could cross-reference two individuals from Villaricos to a comprehensive list of Punic tombs (Astruc 1951). One individual was missing from this list and therefore originates likely from a later burial, so we excluded it from our main analysis.

[6] The individual sampled from Granada with the highest sequence coverage (I22082) had only 29,000 sequenced SNPs, so we excluded Granada from the set of sites in the main analyses.

Section 3 - Modeling ancestry and admixture using ADMIXTURE and qpADM

ADMIXTURE ancestry models

We applied *ADMIXTURE* in unsupervised mode to 122 Phoenician and Punic individuals from our data set that had at least 100,000 sequenced SNPs, together with 24 additional individuals from related populations (**Methods** and **Supplementary Table 12**). We considered models with K=2..5 latent ancestry components; the best-fit model for each value of K is depicted in **Extended Data Figure 2**. When using K=2 latent ancestry components, one component (red) associates with North African ancestry (the four North African individuals in our reference set), and another component (blue) associates with Mediterranean or European ancestry. Individuals from the Levant appear as mixtures of these two components. When using K=3 latent ancestry components, the Mediterranean component splits into two: one corresponding to eastern-associated ancestry (green) and another associated with central-western Mediterranean ancestry (blue). Individuals from Anatolia appear to have a mixture of the eastern and western components. Individuals from Greece and Sicily appear to have mixtures of the central-western Mediterranean component and the Levantine component.

When using K=4 latent ancestry components, the eastern ancestry component splits into two: one component associated with individuals from the eastern Mediterranean (purple) and one component associated with Iran (green). When using K=5 latent ancestry components, the added ancestry component (orange) appears to mostly capture noise, since it is inferred in individuals from many different unrelated sites (including the Iron Age individual from Algeria). When measuring model fit using the ΔK score of Evanno et al. (2005), we find that the model with K=3 provides the best fit, with ΔK higher for K=3 (ΔK=2.524356) than for K=4 (ΔK=1.844891; **Extended Data Figure 2**).

Overall, the *ADMIXTURE* analysis reveals patterns that are qualitatively similar to those that emerge from examining the 2D PCA. Specifically, Phoenician individuals from Akhziv are inferred to be more similar in their ancestry to individuals from the Levant and Iran, whereas individuals from Punic sites share most of their ancestry with Bronze Age individuals from the central or western Mediterranean. In addition, we observe North African-associated ancestry in many individuals from different Punic sites in all four regions examined outside of the Levant.

Ancestry modeling using *ADMIXTURE* is limited in its capabilities to describe the complex ancestry patterns of this diverse population. Because of the unsupervised nature of the method, the interpretation of the inferred ancestry components is also not straightforward. For example, while the red components in **Extended Data Figure 2** clearly correspond to North African ancestry, it is likely that this ancestry component contains some other Mediterranean ancestry as well. This is because we likely have very few samples in the analyzed data set with ancestry derived entirely from non-admixed North African indigenous groups. Similarly, the green component inferred for the K=3 model also likely

corresponds to some mixture of Levantine and central Mediterranean ancestry. As a result, we expect the proportion of North African (or Levantine) ancestry to be lower than the fraction associated with the red (or green) ancestry components. In particular, the prevalent eastern Mediterranean ancestry inferred for individuals in our dataset (green component) is not reflected by locations of the PCA projections for most individuals in our data set (excluding individuals from Akhziv, two individuals from Sicily, and one from Tharros (**Extended Data Figure 1**)). The supervised approach of *qpAdm*, which allows formal testing of the fits of models to data, allowed us to rigorously explore the range of demographic scenarios consistent with the data and more robustly infer Levantine ancestry.

Using *qpAdm* to infer admixture models for individuals

We tested a range of admixture models with *qpAdm* and applied them to each of the 140 Phoenician and Punic individuals in our dataset. We started by considering a broad ancestry model, using 23 ancient individuals from 14 groups to model background ancestry (as "right pops"), and all $255=2^8-1$ non-empty subsets of the eight potential proxy sources (**Supplementary Table 7; Methods**). Many individuals were inferred to have multiple valid admixture models (with non-negative admixture proportions and P-value above 0.05) that were difficult to reconcile with each other. In particular, individuals could be modeled with high proportions of Levantine ancestry (using the proxy population Levant MLBA), or alternatively with no Levantine ancestry. **Supplementary Figure 4** below demonstrates this for 12 individuals from different sites. All individuals in this set, other than I22252 from Akhziv can be modeled either using large fractions of Levantine ancestry (dark red) or without it. This ambiguity was observed for many individuals, making it difficult to differentiate between different ancestry patterns of individuals from different sites.

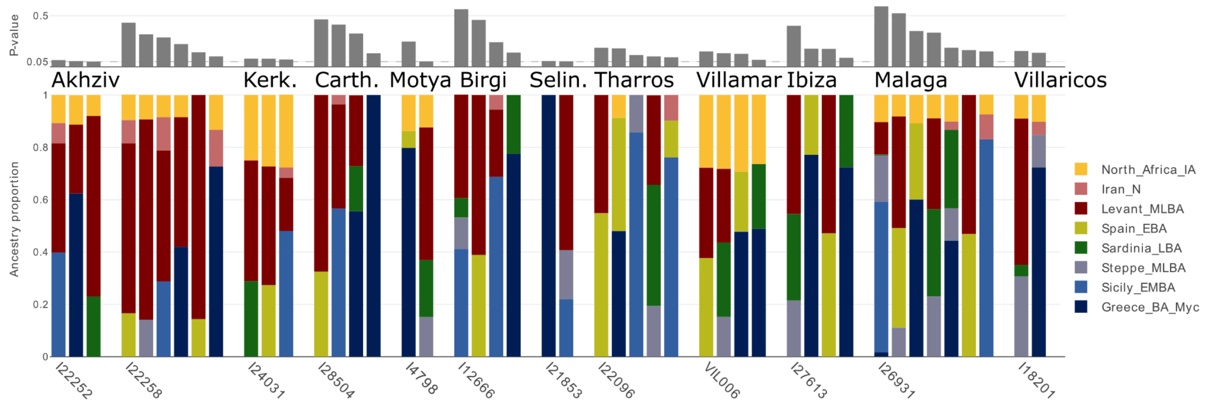

Supplementary Figure 4: Parsimonious models inferred using *qpAdm* under the broad ancestry model for a select subset of individuals. We selected 12 individuals from 11 different Phoenician and Punic sites to show that when only the basic 14 groups are used to model background ancestry (right populations), we can fit "eastern ancestry" models as well as "western ancestry" models for the same individual. Individual IDs are indicated below the vertical bars and site names above them.

We thus decided to consider two separate and distinct types of ancestry models. In the **western ancestry** models, we excluded Levant MLBA from the set of proxy sources and added it to the set of 14 right populations, and considered the 127 non-empty subsets of the seven remaining groups as potential source (left) populations. In the **eastern ancestry**

models, we excluded the five central and western Mediterranean sources and added them to the set of 14 right populations, and we considered the seven non-empty subsets of Levant MLBA, Iran N, and North Africa IA as potential source (left) populations. We tested each of the 140 individuals in our Phoenician/Punic data set for each of the 127 western ancestry models and each of the seven eastern ancestry models. Using a P-value threshold of 0.05, we obtained valid ancestry models for 135 out of the 140 individuals (96.4%) in the data set. Our expectation was that samples that cluster in the PCA near Bronze Age individuals from the Levant would have valid eastern ancestry models, and the remaining samples would have valid western ancestry models. Thus, we examined the types of valid models we get for each individual based on its location in the 2D PCA. The results of this examination are summarized in **Supplementary Table 17** below.

Supplementary Table 17: Numbers of individuals inferred with valid eastern and western ancestry models. We separately consider the 16 individuals clustered in the 2D PCA near Bronze Age individuals from the Levant (**Figure 1**), from the remaining 124 Punic individuals in our data set.

**Types of valid ancestry model**	**Samples that cluster in the PCA near Bronze Age Levant samples (n=16)**	**All other samples (n=124)**
Only western models	0	**109**
Western and eastern models	3 (I22258, I11806, I22271)	14 (I35329, I24205, I24206, I24036, I22093, VIL004, VIL009, I21854, I22090, I22094, I18193, I27610, I27075, I27077)
Only eastern models	**9**	0
No valid eastern or western ancestry models	4 (I11794, I11804, I22251, I12665)	1 (I22122)

First, we see that 118 of the 135 individuals for which we obtained valid ancestry models (87%) had a valid model exclusively of the type we expected according to the PCA (highlighted in bold with gray background). The remaining 17 individuals could be modeled using both ancestry models. Eleven of these individuals had low-coverage sequence data (fewer than 100,000 SNPs), which likely resulted in weak statistical power for rejecting admixture models. Four additional individuals (I24205, I24206, I24036, and I18193) were inferred to have more than 70% North African ancestry, resulting in weak statistical power for inferring the source of contribution for the remaining ancestry. The remaining two individuals (I22258 and I11806) were from Akhziv and had reasonable sequencing coverage (101,000 SNPs and 355,000 SNPs, respectively). The western ancestry models inferred for these individuals attribute more than 70% of their ancestry to the Greece BA (Myc) proxy source, which is highly unlikely given their similarity to Levantine individuals and other individuals from Akhziv in the PCA (Figure 1b) and in the *ADMIXTURE* analysis (**Extended Data Figure 2**). We hypothesize that these western models were not rejected by *qpAdm* due to a lack of resolution in the collection of right populations. This hypothesis is consistent with our observations from the preliminary analysis considering broad ancestry models (see above).

To simplify the presentation of different ancestry models fit for every individual in our dataset, we used a *parsimonious* approach, whereby we report the models with the fewest

sources that also provide a fit to the data (see **Methods**). The average number of parsimonious models per individual was 2.8. All parsimonious valid models we inferred are depicted in **Extended Data Figure 3**. To provide a more complete view of ancestry in our dataset, we tested whether the five individuals for which no valid eastern or western ancestry model was found could be fit using a broad ancestry model (using only the base set of 14 groups as right populations). Individual I22122 from Tharros could not be modeled even using this more relaxed approach. However, we could fit broad ancestry models to the remaining four individuals, which cluster in the PCA near Bronze Age Levant samples (see **Extended Data Figure 3** for inferred models). Three of these individuals were modeled with no contribution from the five western proxy sources, and one individual (I11794 from Akhziv) was inferred to have a small contribution (<10%) from western source proxies. Thus, we conclude that the detailed admixture models inferred using *qpAdm* support the observation from the 2D PCA of genetic separation between eastern Phoenician individuals and western Punic individuals.

Ambiguities observed in individual admixture models across the dataset

Many individuals in our data set were fitted with more than one valid parsimonious admixture model (**Extended Data Figure 3**), and some patterns of model ambiguity appear to recur across the data set. However, other aspects of the fitted models are stable. In particular, the proportion of inferred North African ancestry is consistent across all valid models inferred for every individual. This allowed us to provide a reliable inference about North African ancestry in our sample set (see below). One major contributor to modeling ambiguity is the amount of data per individual. We observed that samples with fewer than 100,000 sequenced SNPs were often associated with very different parsimonious models, because of the lack of statistical power to confidently reject models using *qpAdm* (see, e.g., I27610 from Ibiza and I21854 from Selinunte). Another source of ambiguity has to do with differentiating between eastern and western ancestries, particularly between the contribution of the Levant MLBA proxy and the Greece BA (Myc) proxy. This was observed in two high-coverage individuals from Akhziv (see above), and also in our analysis using broad ancestry models.

Within eastern ancestry models there is very little ambiguity, mostly because the three proxy sources used in these models are highly differentiated. On the other hand, western ancestry models are associated with much more ambiguity, mostly within the western Mediterranean proxies (Sardinia/Iberia LBA) and also within the two proxies representing Sicilian-Aegean ancestry (Greece BA (Myc) and Sicily EBA). A concrete example is provided by the six different parsimonious models inferred for individual I22115 from Tharros (see **Extended Data Figure 3**). These models suggest that this individual has a significant proportion of Sicilian-Aegean ancestry), and a significant amount of western Mediterranean ancestry. However, our modeling approach could not reliably identify the exact sources. Similar patterns are observed for other individuals in our data set. Thus, when presenting representative ancestry models in **Figure 2**, we exclude the low-coverage samples, and we group source pairs that cannot be distinguished reliably (Greece and Sicily; Sardinia and Iberia).

North African ancestry in Punic individuals

To provide a more comprehensive summary of North African ancestry, we computed estimates of North African ancestry based on the 2D PCA plot (**Figure 1**) and compared

these estimates to the ones obtained by *qpAdm*. This analysis included a total of 123 Punic individuals, excluding the 13 individuals from Akhziv, three additional individuals that cluster next to them in the PCA (I12665 and I21856 from Sicily and I22119 from Sardinia), and one individual for which we did not obtain valid ancestry models using *qpAdm* (I22122). As noted above, there appears to be little ambiguity regarding the proportions of North African ancestry across different valid models inferred by *qpAdm* for each individual. We determined a conservative *qpAdm*-based estimate of North African ancestry for each individual based on the *smallest* proportion of North African ancestry inferred for it in a valid and parsimonious *qpAdm* model (**Extended Data Figure 3**). The PCA-based estimate of North African ancestry was determined based on the location of a given individual along a cline from the Sicilian-Aegean cluster to the North African cluster. This cline was defined using the straight line segment between the Iron Age individual from Khenkela, Algeria (I12433) to the edge of the cluster defined by Bronze Age samples from Sicily (light blue line in **Supplementary Figure 5**). The PCA-based estimate of North African ancestry was set to be the distance of the projected point from the straight line's top-left edge divided by the line's total length. Individuals whose projection falls above the top-left point were assigned a zero PCA-based estimate of North African ancestry. This computation was implemented using a linear formula given the two first PC coordinates of a given individual (PC_1, PC_2):

$$\text{PCA-based estimate} = \max\left\{0\,;\ 16.02 \times PC_1 - 13.37 \times PC_2 - 0.3261\right\}.$$

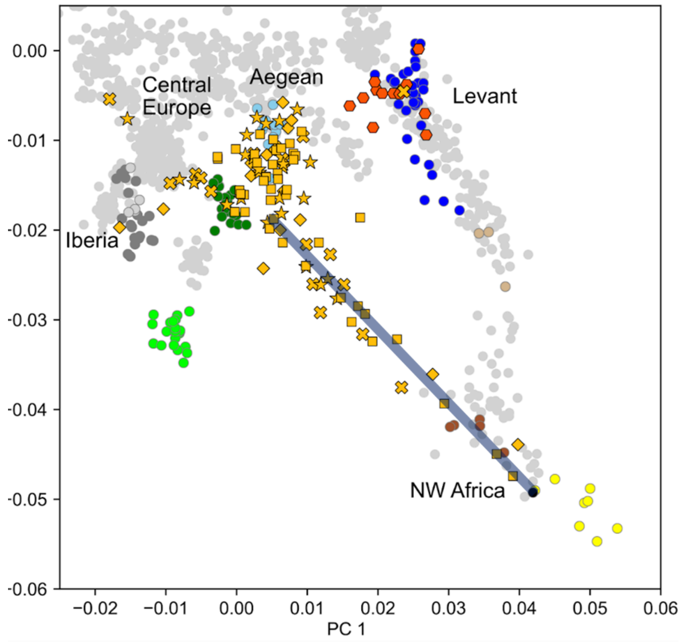

Supplementary Figure 5: The 2D PCA from Figure 1, with a straight line depicting the cline of North African ancestry. The line (blue) is stretched from the Iron Age individual from Khenkela, Algeria (I12433) to the edge of the cluster defined by Bronze Age samples from Sicily. The PCA-based estimate of North African ancestry is defined as the relative location of the projection of an individual in this plot onto this straight line.

The PCA- and *qpAdm*-based estimates of North African ancestry for the 123 Punic individuals are shown in **Extended Data Figure 4**. Comparing the two sets of estimates, we see that both approaches lead to similar estimates of North African ancestry, with the model-based approach of *qpAdm* being more sensitive when the ancestry proportions are low (<30%). A detailed examination of the inferred proportions of North African ancestry in different sites is described in the following sections.

Ancestry patterns observed in Akhziv

The 13 individuals from Akhziv have relatively homogeneous ancestries and are genetically very similar to earlier populations in the Levant, as indicated by the PCA plot. Eight of them could be modeled as 100% Levant MLBA (one of them using a broad ancestry model). Four remaining individuals were inferred to have more than 78% of their ancestry associated with the Levant MLBA proxy source, and the remaining ancestry was typically attributed to either Iran N or North Africa IA. One individual (I12251) was inferred to have as much as 40% Iranian ancestry (using a broad ancestry model), and one individual (I11794) was inferred to have a small fraction of western ancestry (also in a broad ancestry model).

Ancestry patterns observed in Kerkouane and Carthage

Individuals sampled from Kerkouane have mainly Sicilian-Aegean ancestry, with a broad range of North African ancestry. For ten individuals we inferred no significant North African ancestry in *qpAdm* as well as the PCA-based approach (see above). Three individuals were inferred to have more than 67% North African ancestry by both approaches, and the remaining 14 individuals populated the remaining range. In Carthage, we estimated lower proportions of North African ancestry: in 14 out of the 17 sampled individuals, we inferred less than 15% North African ancestry (by both approaches), and the remaining three individuals were inferred to have 20% - 50% North African ancestry. As in Kerkouane, the primary source of ancestry is Sicilian-Aegean. In Carthage, we also see a small yet significant contribution from the Iran (N) source. For six individuals, all valid *qpAdm* models contained a positive contribution from this proxy source. We note that Iranian ancestry could originate from gene flow from Anatolia (Skourtanioti et al. 2020; Lazaridis et al. 2022). Interestingly, very few individuals in our data set from other sites show this ancestry pattern, most of which were sampled in Carthage.

Ancestry patterns observed in Sicily

Ancestry patterns in Sicily until the 2nd century BCE are similar to what we see in Kerkouane but with smaller proportions of inferred North African ancestry. Of the 25 individuals sampled from Sicily dated to 800 BCE - 170 BCE, 12 were inferred to have no North African ancestry. Among the remaining 13 individuals, only two had more than 20% inferred North African ancestry (I22232 from Motya and I21194 from Selinunte). We did not observe any notable shifts in proportions of North African ancestry between the two time ranges, before and after 400 BCE. As a point for comparison, we also applied the same *qpAdm* analysis to 23 suitable individuals sampled from indigenous sites in Sicily: 19 individuals from the Iron Age site of Polizzello (Reitsema et al. 2022) and four individuals sampled from Monte Falcone, Baucina, which was active between the 9th and 4th centuries BCE (Reitsema et al. 2022). The inferred models are depicted in **Extended Data Figure 9a**. We see very similar patterns of ancestry in these two sites as observed in the Punic sites, with a clear absence of North African ancestry. Indeed, none of these 23 individuals required North African ancestry for a valid model. This suggests that North African ancestry entered Sicily through connections between its Punic settlements and those in North Africa (see also **Figure 4**).

We also examined patterns of ancestry in Phoenician/Punic sites in Sicily after the 2nd century BCE, which were likely influenced by Roman expansion following the Punic wars. To this end, we examined the seven individuals dated to 400 BCE - 50 BCE (**Extended Data Figure 9b**), as well as 15 individuals dated to time ranges postdating 200

BCE (**Extended Data Figure 9c**). In 6 out of this set of 22 individuals, we inferred a significant contribution from Levantine ancestry, with four individuals inferred to have more than 80% Levantine ancestry. Two of these individuals (I12665 and I21856) were radiocarbon dated to 400 BCE - 50 BCE and thus possibly overlap with Punic influence in these sites. However, the expansion of Levantine ancestry appears to be associated with the Roman control of Sicily. We also see low, yet significant, levels of Iranian ancestry in 7 individuals, which is likely associated with some gene flow from Anatolia (see explanation above for individuals from Carthage). Two individuals from Lilybaeum dated to 400 BCE - 50 BCE (I21859 and I8577) were inferred to have substantial proportions (>40%) of western Mediterranean ancestry (Iberia/Sardinia), consistent with their location in the 2D PCA plot (toward the left; see **Extended Data Figure 1**). We note that two individuals postdating 200 BCE were not fit with admixture models, and other individuals were fit with relatively low P-values (**Extended Data Figure 9c**), suggesting additional sources of ancestry not adequately modeled by the framework we developed for Phoenician and Punic individuals. Overall, our findings suggest that Roman expansion into Sicily introduced diverse ancestries that were not prevalent during the Punic era. Still, there is some continuity in ancestry, as many individuals were inferred to have high proportions of Sicilian-Aegean ancestry, and North African ancestry was quite prevalent as late as the 2nd century CE. These findings are consistent with shifts in ancestry reported in ancient DNA studies of Roman populations (Antonio et al. 2019; Zaro et al. 2021). In particular, three individuals sampled near Rome dating to 400 BCE - 50 BCE were previously reported to have high proportions of Levantine ancestry (Antonio et al. 2019; Ravasini et al. 2024), similar to what we observe in some of the individuals from Sicily during this time period.

Ancestry patterns observed in Sardinia

Generally, the ancestry patterns observed in Sardinian samples are similar to those observed in Sicilian samples. Sicilian-Aegean ancestry is dominant, with a substantial contribution from North African ancestry. Notably, we do not observe an excess in ancestry originating in Bronze Age Sardinian populations. This is consistent with differences between Bronze Age and Punic samples from Sardinia in the PCA plot (**Extended Data Figure 1**). Interestingly, North African ancestry appears to have arrived in Sardinia later than it did in Sicily. Five of the seven individuals dated before 400 BCE (six from Tharros, one from Monte Sirai) were inferred to have no North African ancestry, and the remaining two individuals were inferred to have less than 10% of their ancestry originating in North Africa. On the other hand, out of the ten individuals dated between 400 BCE and 200 BCE (six from Villamar and four from Tharros), five were inferred to have more than 20% North African ancestry. This is particularly striking in Tharros, where five individuals dated before 400 BCE with an average estimate of North African ancestry below 5%, and seven individuals dated after 400 BCE with an average estimate of North African ancestry above 25%. This would suggest that North African ancestry was introduced into the maritime settlement of the port of Tharros (at least individuals buried in the northern necropolis) after the 5th century BCE, at a time when it was already established in Punic sites in Sicily (Birgi and Motya) and Kerkouane.

Ancestry patterns observed in Iberia

Ancestry patterns in Iberia are consistent with what we see in other regions, with a dominant contribution from the Sicilian-Aegean source. As in Sardinia, this contrasts with the ancestry

patterns observed in Bronze Age populations, which are notably different. Indeed, we do not observe a higher contribution of western Mediterranean ancestry in most Punic individuals we sampled. Notable exceptions are I8135 from Cadiz and I27618 from Ibiza, for which we infer more than 80% ancestry derived from a Bronze Age Iberian population. On the other hand, the five family members from tomb 774 in Villaricos (**Figure 5**) were inferred to have Sicilian-Aegean ancestry and a small fraction (<10%) of North African ancestry. Regarding the contribution of North African ancestry to other individuals in our Iberian sample set, two individuals from Villaricos (I18189 and I18193) were inferred to have large proportions (65% and 88%), but for the remaining individuals, we inferred contributions below 25%. Our data set contains only one individual dated before 400 BCE (I12517 from Cadiz), for which no North African ancestry was inferred. Thus, with our current sample set we cannot conclude anything with confidence regarding the time when North African ancestry arrived in Iberia.

Features and limitations of our admixture models

The *qpAdm* admixture models we considered have a few key properties that allow an effective analysis. First, they provide valid models for almost all (96%) of Phoenician and Punic individuals in our data set, allowing us to characterize ancestry throughout the dataset. The models also clearly differentiate between individuals with a Levantine component in their ancestry (eastern models) and those without it (western models). Thus, we were able to conclude that individuals who did not have a dominant Levantine ancestry component could be modeled with no Levantine ancestry (and with Levant_MLBA in the set of right populations). Another important feature of our model is using a newly sequenced Iron Age individual from Khenkela (Algeria) to model North African ancestry. Together with the use of Neolithic Individuals from Tunisia as a background (right) population, this enabled informative estimates of North African ancestry in our data set. As a result, ancestry associated with this Algeria IA proxy source likely reflects a source population that is more closely related to the Iron Age Algerian individual than it is to earlier Neolithic populations. The fact that we were able to model African ancestry in our data set using this approach implies that there is no significant contribution from divergent African ancestries to Punic genetic diversity. Moreover, the comparison with the PCA-based estimates (**Extended Data Figure 4**) suggests that our model has a high sensitivity for detecting small amounts of North African ancestry, and the fact that no North African ancestry was inferred for Iron Age individuals from Sicily sampled in non-Phoenician/Punic sites (**Extended Data Figure 9a**) suggests that it does not result in spurious inference.

Our modeling approach also has limitations. First, it does not allow us to reliably model a combination of Levantine ancestry with a western ancestry component. The broad ancestry model, which considers all eight groups and potential proxy sources, produced informative admixture models for one individual in Akhziv (I11794) and three Roman-era individuals from Sicily (**Extended Data Figure 9c**). However, generally, this model could not effectively distinguish between Levantine ancestry and Greek ancestry (see discussion above on model ambiguity). Thus, while analysis suggests that we can model most individuals without Levantine ancestry, we cannot completely rule out the possibility that some of them may be better modeled using admixture between a Levantine source and a Sicilian-Aegean source. Another limitation of our current modeling approach is that it does not provide accurate information regarding the source of the Sicilian-Aegean ancestry, which we find to be dominant in our data set. The source appears to be closely related to Bronze

Age populations in Greece and/or Sicily, but the exact relationship between the source and these populations is unclear from our genetic analysis alone. This lack of resolution is mostly due to the sparse sampling of Bronze Age individuals from the eastern Mediterranean. In particular, we have no sampling from the Mediterranean coast of Asia Minor or Cyprus, both of which plausibly could be origins for this ancestry component.

Section 4 - Prevalence of J2a and J2b Y chromosome haplogroups

To assess the hypothesis that specific J2 Y haplogroups trace back to a “Phoenician footprint across the Mediterranean” (P. A. Zalloua et al. 2008), we measured the frequency of both J2a and J2b Y haplogroups in the published aDNA record, grouping individuals based on the cluster labels of the Allen Ancient DNA resource (AADR). We considered groups with at least four males with sufficient sequencing coverage. We then analyzed the Y haplogroups of males in these groups (see **Methods**) to the level of the first three letters in the ISOGG19 nomenclature. **Supplementary Tables 186** and **19** below list statistics for the groups with the highest frequency of J2a and J2b.

J2a appears relatively frequently in Aegean Bronze Age groups (in many groups observed at >30% frequency) and notably in the classic Greek sample from Himera, Sicily (2 out of 7 males with inferred J2a). In contrast, when combining Levantine Bronze and Iron Age clusters (Lebanon_IA, Lebanon_MBA, Israel_MLBA, Israel_IA), J2a only appears in 3 of 32 males and similarly low frequencies in our Punic dataset (8 of 58 males).

J2b reaches exceptionally high frequencies in various Balkan and Greek Bronze and Iron Age sites (>75% of the Y haplogroup calls in Montenegro_MLBA, Greece_Mygdalia_LBA, Croatia_MBA_Cetina, Croatia_EIA, and Croatia_MBA). In the combined Levantine Bronze and Iron Age clusters, this haplogroup only appears in 2 of 32 males, and we observe a similarly low J2b frequency in our Punic dataset (3 of 58 males).

The dominant Y haplogroup in the Levant Bronze and Iron Age cluster is not J2 but J1a (16 of 32 males), which we find in only 4 of 52 Punic males in our dataset. This signal of substantially different Y haplogroup patterns is consistent with the autosomal signal of little genetic ancestry in Punic individuals deriving from the Levant.

Supplementary Table 18: Archaeological cluster labels with highest frequencies of J2a Y haplogroups (and subgroups). At the bottom, we added four relevant groups from the Bronze and Iron Age Levant as well as the set of all Punic males.

AADR Cluster Label	Nr. Y haplotypes	Nr. J2a Calls	Fraction J2a
Iran_ShahrISokhta_BA1	5	4	0.8
Turkey_WestByzantine	4	3	0.75
Greece_Crete_HgCharalambos_EMBA	18	13	0.7222222222
Uzbekistan_Bustan_BA	4	2	0.5
Spain_Islamic	4	2	0.5
NorthMacedonia_IA	4	2	0.5
Turkey_Arslantepe_LateC	12	5	0.4166666667
Greece_Crete_Chania_LBA	10	4	0.4
Turkey_EarlyByzantine_2	5	2	0.4
Italy_IsolaSacra_RomanImperial.SG	5	2	0.4
Turkey_TellAtchana_MLBA	8	3	0.375
Turkey_SoutheastByzantine	8	3	0.375
Turkey_Ottoman_ArabGraves	6	2	0.3333333333
Turkey_Byzantine	6	2	0.3333333333
Kyrgyzstan_TianShan_Saka.SG	6	2	0.3333333333
Italy_LA.SG	9	3	0.3333333333
Germany_Medieval_Jewish	7	2	0.2857142857
Italy_Sicily_Himera_480BCE_Greek	7	2	0.2857142857
Kazakhstan_Medieval_Nomad.SG	4	1	0.25
Pakistan_Katelai_IA	12	3	0.25
Turkmenistan_Gonur_BA_1	4	1	0.25
Croatia_Zadar_Roman.SG	4	1	0.25
Iran_C_TepeHissar	4	1	0.25
Iran_Hasanlu_IA	8	2	0.25
Mongolia_Khuvsgul_XiongnuLateMedieval_2	4	1	0.25
Italy_Imperial.SG	16	4	0.25
Bulgaria_EBA	5	1	0.2
Croatia_Popova_MN.SG	5	1	0.2
Italy_Basilicata_Venosa	5	1	0.2
Italy_Sicily_EBA	5	1	0.2
Lebanon_IA	8	0	0.0
Lebanon_MBA	2	0	0.0
Israel_MLBA	20	3	0.15
Israel_IA	2	0	0.0
All Punic	58	8	0.14

Supplementary Table 19: Archaeological cluster labels with highest frequencies of J2b Y haplogroups (and subgroups). All cluster labels with at least five males and one J2b call are shown (and not only the most frequent occurrences as in the J2a table, where space is a limiting factor).

AADR Cluster Label	Nr. Y haplotypes	Nr. J2b Calls	Fraction J2b
Montenegro_MLBA	5	5	1
Greece_Mygdalia_LBA.rel	4	4	1
Croatia_MBA_Cetina	5	4	0.8
Croatia_EIA	8	6	0.75
Croatia_MBA	4	3	0.75
Italy_Basilicata_Venosa	5	3	0.6
Italy_Sardinia_BA_Nuragic	6	3	0.5
Slovenia_EIA	7	2	0.2857142857
Germany_Anderten_Saxon_Medieval	7	2	0.2857142857
Albania_BA_IA	4	1	0.25
Uzbekistan_Bustan_BA	4	1	0.25
Italy_Medieval_EarlyModern.SG	10	2	0.2
Italy_IA_Republic.SG	5	1	0.2
India_RoopkundB	6	1	0.1666666667
Pakistan_Loebanr_IA	16	2	0.125
Italy_Tuscany_Grosseto_Etruscan	9	1	0.1111111111
Turkey_Alalakh_MLBA	9	1	0.1111111111
India_RoopkundA	11	1	0.09090909091
Italy_Imperial.SG	16	1	0.0625
Israel_MLBA	18	1	0.05555555556
England_EarlyMedieval_Saxon	24	1	0.04166666667
Hungary_Conqueror_Elite.SG	33	1	0.0303030303
Sweden_Viking.SG	75	1	0.01333333333
Lebanon_IA	8	0	0.0
Lebanon_MBA	2	1	0.5
Israel_MLBA	20	1	0.05
Israel_IA	2	0	0.0
All Punic	58	3	0.052

Section 5 - Y Haplogroup and Autosomal Diversity in the Ancient Mediterranean

We list the Y haplogroup and autosomal diversity values depicted in **Figure 3** in two tables, **Supplementary Table 20** and **Supplementary Table 21**, respectively**,** using the Diversity values calculated as described in **Methods**.

We also wished to explore how much of the increased PCA and Y haplogroup diversity we document in Punic sites was driven by North African ancestry. North African ancestry is highly differentiated from Western Eurasian ancestries north of the Mediterranean, on the level typical for differing continental groups (measured pairwise F_{ST} values typically >0.05, see **Supplementary Figure 6**), likely due to the prior barrier of gene flow posed by the Mediterranean Sea (Fregel et al. 2018; van de Loosdrecht et al. 2018). Therefore, since North African ancestry is present in a wide range of proportions across Punic individuals, it could be a primary driving factor for the increased diversity we record in **Figure 3**.

We investigated this by calculating “Non-African” genetic diversity for Punic sites. For autosomal (PCA-based) diversity, we did this by removing all Punic individuals with North African ancestry proportions >10%, as inferred by qpAdm (see **Extended Data Figure 4**). While the ancestry diversity in PCA declined (mainly in North African sites Kerkouane and Carthage), it was still relatively elevated in Lilybaeum and Tharros (**Extended Data Figure 6b**). For Y haplogroup diversity, we filtered the haplogroups E1a and L, which are widespread in modern Africa. This filtered one male from Villaricos and one from Kerkouane. Consequently, the Y haplogroup diversity in these two sites slightly decreased but remained higher than nearly all earlier context groups, and the Y haplogroup diversity of other sites remained the same (**Extended Data Figure 6a**).

These patterns suggest that while North African ancestry contributes substantially to the total increased diversity in Punic sites (particularly in North African sites), other Mediterranean ancestry components are also highly diverse compared to earlier Mediterranean and Western Eurasian ancient individuals.

Supplementary Table 20: Y Diversity per Site. Sites are sorted by age.

Archaeological Site	Mean Age BP	Nr. Males	Y Diversity	Plot Label
Northwest Anatolia, Marmara, Barcın	8272.5	9	3.27273	Aegean
Saxony-Anhalt, Mittelelbe-Saaleregion, Derenburg-MeerenstiegII	7100	13	5.57143	Central Europe
Baden-Württemberg, Stuttgart-Mühlhausen I	7096	8	1.00000	Central Europe
Asparn Schletz	6950	44	3.21769	Central Europe
Yonne, Gurgy "les Noisats"	6450	13	1.41818	Central Europe
Calvados, Fleury-sur-Orne	6326	9	2.76923	Central Europe
Upper Galilee, Peki'in	5950	7	1.40000	Levant
Malatya Province, Arslantepe	5227	12	5.07692	Aegean
Marne, Mont-Aimé hypogée II	5162	5	1.66667	Central Europe
Hesse, Niedertiefenbach	5143	23	1.00000	Central Europe
Mandubi Zelaia	5050	5	1.00000	Iberia
Catalonia, Barcelona, Cova de la Guineu	4900	6	5.00000	Iberia
Alicante, Villena, Cueva de las Lechuzas	4750	6	2.50000	Iberia
Burgos, Atapuerca, El Mirador Cave	4550	5	1.66667	Iberia
Central Greece, Euboea	4421	5	10.00000	Aegean
Esperstedt	4225	9	1.71429	Central Europe
Irlbach LKR	4200	6	1.00000	Central Europe
Crete, Lasithi	4050	21	2.35955	Aegean
Southern Singen	3900	8	1.00000	Central Europe
Murcia, Totana, La Bastida	3825	6	1.50000	Iberia
Sardinia BA	3819.5	5	2.50000	BA/IA Context
Murcia, Pliego, La Almoloya	3801.5	28	1.00000	Iberia
Kleinaitingen - Gewerbegebiet Nord	3750.5	10	1.00000	Central Europe
Hatay Province, Tell Atchana/Alalakh	3689.5	7	10.50000	Aegean
Hatay, Tell Atchana (Alalakh)	3550	8	7.00000	Aegean
Canaanite MLBA	3500	16	3.75000	BA/IA Context
Jezreel Valley, Megiddo	3500	15	3.28125	Levant
Western Greece / Peloponnese, Achaea	3473	5	1.66667	Aegean
Crete, Chania	3225	10	5.00000	Aegean
Sardinia, Perdasdefogu, NUO, S’Orcu ‘e Tueri	3148	5	3.33333	Italy
Sicily Polizzello IA	2650	7	1.00000	BA/IA Context
Sicily, Polizzello	2650	7	1.00000	Italy
Akhziv	2600	6	2.50000	Phoenician
Değirmendere (Aegean, Muğla, Yatağan)	2565	5	1.66667	Aegean
Kerkouane	2500	8	14.00000	Punic
Sicily, Selinunte, Manuzza	2482	6	15.00000	Punic
Sicily, Himera	2430	19	12.21429	Italy
Sardinia, Tharros	2400	6	15.00000	Punic
Beirut	2385	7	3.50000	Levant
Aude, Le Cailar "Place de la Saint-Jean"	2300	5	1.66667	Central Europe
Tuscany, Grosseto, Casenovole	2300	5	1.66667	Italy

Tunis, Carthage	2300	8	14.00000	Punic
Tarquinia Monterozzi	2253	8	2.80000	Italy
Marne, Chemin de Coupetz, Faux Vesigneul	2200	8	1.33333	Central Europe
Lazio, Viterbo, Tarquinia	2151	10	1.55172	Italy
Via Paisiello (Necropoli Salaria)	1850	5	10.00000	Italy
Monterotondo	1814	5	5.00000	Italy
Isola Sacra	1750	7	5.25000	Italy
Sarrebourg, Marxberg Necropolis	1609	7	10.50000	Central Europe
Crypta Balbi	1450	5	10.00000	Italy
Mausoleo di Augusto	1450	5	10.00000	Italy
North Rhine-Westphalia, Alt-Inden	1350	16	3.07692	Central Europe
Samantaş (Aegean, Muğla, Yatağan)	1346	6	3.75000	Aegean
Niederstotzingen, Southern Germany	1345	8	1.33333	Central Europe
Piedmont, Collegno	1345	15	2.33333	Italy
Basilicata, Potenza, Venosa	1250	5	3.33333	Italy
Lower Saxony, Drantum	1200	5	10.00000	Central Europe
Lower Saxony, Anderten	1150	9	4.00000	Central Europe
Lower Saxony, Dunum	1050	8	1.86667	Central Europe
Schleswig-Holstein, Schleswig	850	12	3.14286	Central Europe
Sidon	800	9	3.60000	Levant
Tilbeşar Höyük (Southeast, Gaziantep)	750	5	3.33333	Aegean
Stratonikeia-West Church (Aegean, Muğla, Yatağan)	650	5	10.00000	Aegean
Erfurt, Ackerhof	625	8	4.00000	Central Europe
Villa Magna	595	7	5.25000	Italy
Cancelleria	514	6	15.00000	Italy
Çapalıbağ, Yeşilbağcılar-YTEUAS (Aegean, Muğla, Yatağan)	475	6	15.00000	Aegean

Supplementary Table 21: PCA Diversity per Site. Sites are sorted by age.

Archaeological Site	Mean Age BP	Nr. Individuals	PCA Diversity	Plot Label
Barcın (Northwest Anatolia, Marmara)	8247.5	14	0.0044683	Aegean
Baden-Württemberg, Stuttgart-Mühlhausen I	7096	16	0.0043145	Central Europe
"France, Yonne, Gurgy ""les Noisats"" Family A	6450	11	0.0035034	Central Europe
Calvados, Fleury-sur-Orne	6350.5	10	0.0045128	Central Europe
Upper Galilee, Peki'in	5950	17	0.0044670	Levant
Çorum Province, Çamlıbel Tarlası	5499	12	0.0042342	Aegean
Samsun Province, İkiztepe	5394	10	0.0070494	Aegean
Malatya Province, Arslantepe	5219.5	14	0.0101088	Aegean
Barcelona, Cova de la Guineu	4900	10	0.0052136	Iberia
Alicante, Villena, Cueva de las Lechuzas	4750	10	0.0030587	Iberia
Burgos, Atapuerca, El Mirador Cave	4550	14	0.0055301	Iberia
Esperstedt	4225	15	0.0142666	Central Europe
Southern Germany, Singen	3915	14	0.0073383	Central Europe
Königsbrunn - Obere Kreuzstraße (Baugebiet 110)	3914.5	10	0.0068104	Central Europe
Murcia, Pliego, La Almoloya	3825	66	0.0048777	Iberia
Sardinia BA	3775	11	0.0027715	BA/IA Context
Kleinaitingen - Gewerbegebiet Nord	3748	17	0.0073665	Central Europe
Hatay Province, Tell Atchana/Alalakh	3722.5	22	0.0108724	Aegean
Hatay, Tell Atchana (Alalakh)	3550	11	0.0161188	Aegean
Canaanite MLBA	3550	35	0.0059595	BA/IA Context
Megiddo, Jezreel Valley	3500	23	0.0116997	Levant
Mycenean BA	3264	12	0.0027096	BA/IA Context
Mecklenburg-Vorpommern, Tollense battlefield	3200	12	0.0132850	Central Europe
Sicily Polizzello IA	2650	19	0.0025901	BA/IA Context
Akhziv	2600	13	0.0085469	Phoenician
Birgi	2582	8	0.0118982	Punic
Değirmendere (Aegean, Muğla, Yatağan)	2565	12	0.0039191	Aegean
Selinunte	2500	7	0.0223019	Punic
Kerkouene	2500	27	0.0275862	Punic
Motya	2485	8	0.0208319	Punic
Iberia IA	2464.5	16	0.0043896	BA/IA Context
Tharros	2400	14	0.0344549	Punic
Beirut	2385	12	0.0109643	Levant
Tuscany, Grosseto, Casenovole	2350	10	0.0072003	Italy
Carthage	2300	17	0.0149675	Punic
Tarquinia Monterozzi	2238.5	10	0.0292618	Italy
Villaricos	2219.5	8	0.0357184	Punic
Girona, Empuries, necropolis Centre de Visitants	2202.5	10	0.0233261	Iberia
Marne, Chemin de Coupetz, Faux Vesigneul	2200	11	0.0081775	Central Europe
Lilybaeum	2160	7	0.0353476	Punic
Lazio, Viterbo, Tarquinia	2151	17	0.0093392	Italy
Isola Sacra	1750	20	0.0247987	Italy

Sarrebourg, Marxberg Necropolis	1671.5	10	0.0223703	Central Europe
Bavaria, Altenerding- Klettham	1455	10	0.0171928	Central Europe
Bavaria, Straubing- Bajuwarenstraße	1437.5	16	0.0209526	Central Europe
North Rhine-Westphalia, Alt-Inden	1350	17	0.0170205	Central Europe
Piedmont, Collegno	1345	22	0.0199196	Italy
Basilicata, Potenza, Venosa	1250	14	0.0138563	Italy
Lower Saxony, Drantum	1200	16	0.0094288	Central Europe
Lower Saxony, Anderten	1150	13	0.0073848	Central Europe
Schleswig-Holstein, Schleswig	850	15	0.0070246	Central Europe
Villa Magna	595	11	0.0123497	Italy
Çapalıbağ, Yeşilbağcılar-YTEUAS (Aegean, Muğla)	475	12	0.0113241	Aegean

Section 6 - Genetic differentiation (FST) between different groups of Mediterranean individuals.

We used pairwise F_{ST} to measure pairwise genetic differentiation between groups of individuals we considered in different parts of our analysis (see **Supplementary Figure 6**). F_{ST} is a standard measure for genetic differentiation that provides a simple way to measure genetic similarity between groups and is also largely independent of the SNP set (Bhatia et al. 2013). We calculated F_{ST} using the function *average_patterson_fst* from the Python package scikit-allel (v1.2.1), reporting the standard error estimated via block-jackknife over blocks of 1000 markers. We used pseudo-haploid data for ancient populations, i.e., one allele picked randomly for each SNP covered with at least one sequencing read.

Notably, we find that absolute genetic differentiation between potential sources of Sicilian and Aegean sources is comparably tiny (e.g., F_{ST} =0.0057±0.0005 for “Greece BA Mycenaean” and “Italy Sicily IA Polizello” and F_{ST} =0.0108±0.0010 for “Greece BA Mycenaean” and “Italy Sicily MBA”), highlighting the challenges in distinguishing these ancestries as sources in ancient genomes of Punic sites.

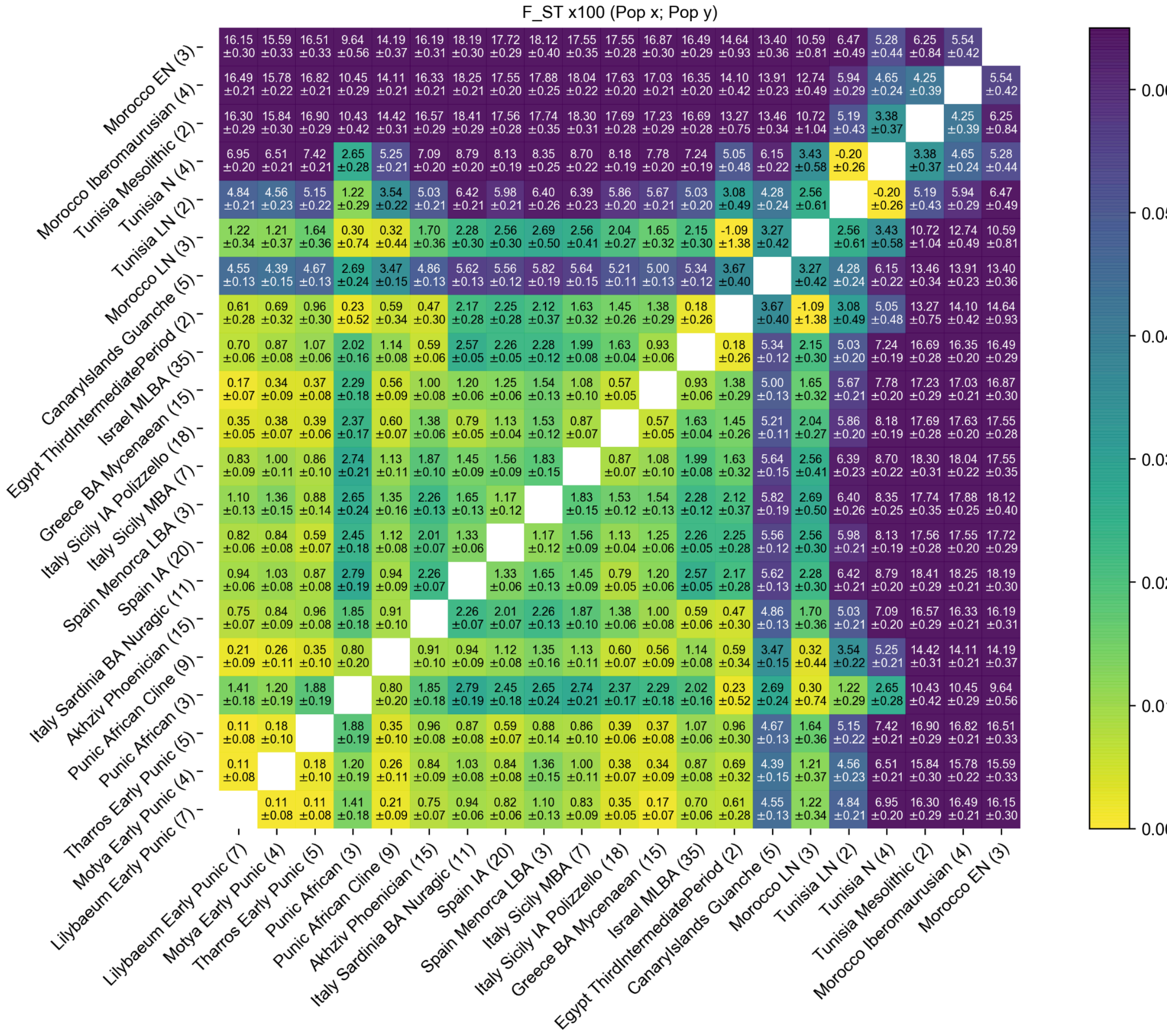

Supplementary Figure 6: Matrix plot of genetic differentiation between relevant ancient groups measured by F_{ST}. We calculated genetic differentiation between groups of individuals (denoted on the x and y axis, sample number given in brackets) as described above. We labeled each group according to its geographic location and time. We partitioned Punic individuals from the early time period (800-400 BCE) by site, removing two individuals that were outliers in the PCA. We further split Punic individuals from the late time period (400-200 BCE) into two groups according to their fraction of North African ancestry inferred based on their location on the 2D PCA (see **Extended Data Figure 4**): individuals with more than 50% North African ancestry were associated with the "Punic African" group and individuals with North African ancestry between 20% and 50% were associated with the "Punic African cline". We specify the mean F_{ST} value (multiplied by 100) and standard error for each pair of populations. Note that we grouped early individuals from the sites Birgi and Lilybaeum, two sites in close proximity, into one cluster, "Lilybaeum Early Punic".

Supplementary References for Sections 2-6

Antonio, Margaret L., Ziyue Gao, Hannah M. Moots, Michaela Lucci, Francesca Candilio, Susanna Sawyer, Victoria Oberreiter, et al. 2019. "Ancient Rome: A Genetic Crossroads of Europe and the Mediterranean." *Science*, November. https://doi.org/10.1126/science.aay6826.

Astruc, Miriam. 1951. *La necropolis de Villaricos*.

Bhatia, Gaurav, Nick Patterson, Sriram Sankararaman, and Alkes L. Price. 2013. "Estimating and Interpreting FST: The Impact of Rare Variants." *Genome Research* 23 (9): 1514–21.

Fregel, Rosa, Fernando L. Méndez, Youssef Bokbot, Dimas Martín-Socas, María D. Camalich-Massieu, Jonathan Santana, Jacob Morales, et al. 2018. "Ancient Genomes from North Africa Evidence Prehistoric Migrations to the Maghreb from Both the Levant and Europe." *Proceedings of the National Academy of Sciences of the United States of America* 115 (26): 6774–79.

Lazaridis, Iosif, Songül Alpaslan-Roodenberg, Ayşe Acar, Ayşen Açıkkol, Anagnostis Agelarakis, Levon Aghikyan, Uğur Akyüz, et al. 2022. "The Genetic History of the Southern Arc: A Bridge between West Asia and Europe." *Science* 377 (6609): eabm4247.

Loosdrecht, Marieke van de, Abdeljalil Bouzouggar, Louise Humphrey, Cosimo Posth, Nick Barton, Ayinuer Aximu-Petri, Birgit Nickel, et al. 2018. "Pleistocene North African Genomes Link Near Eastern and Sub-Saharan African Human Populations." *Science* 360 (6388): 548–52.

Marcus, Joseph H., Cosimo Posth, Harald Ringbauer, Luca Lai, Robin Skeates, Carlo Sidore, Jessica Beckett, et al. 2020. "Genetic History from the Middle Neolithic to Present on the Mediterranean Island of Sardinia." *Nature Communications* 11 (1): 939.

Moots, Hannah M., Margaret Antonio, Susanna Sawyer, Jeffrey P. Spence, Victoria Oberreiter, Clemens L. Weiß, Michaela Lucci, et al. 2023. "A Genetic History of Continuity and Mobility in the Iron Age Central Mediterranean." *Nature Ecology & Evolution*, August. https://doi.org/10.1038/s41559-023-02143-4.

Ramsey, Christopher Bronk. 2009. "Bayesian Analysis of Radiocarbon Dates." *Radiocarbon* 51 (1): 337–60.

Ravasini, Francesco, Cecilia Conati Barbaro, Christiana Lyn Scheib, Kristiina Tambets, Mait Metspalu, Fulvio Cruciani, Beniamino Trombetta, and Eugenia D'Atanasio. 2024. "The Arrival of the Near Eastern Ancestry in Central Italy Predates the Onset of the Roman Empire." *bioRxiv*. https://doi.org/10.1101/2024.10.07.617003.

Reimer, Paula J., William E. N. Austin, Edouard Bard, Alex Bayliss, Paul G. Blackwell, Christopher Bronk Ramsey, Martin Butzin, et al. 2020. "The IntCal20 Northern Hemisphere Radiocarbon Age Calibration Curve (0–55 Cal kBP)." *Radiocarbon* 62 (4): 725–57.

Reitsema, Laurie J., Alissa Mittnik, Britney Kyle, Giulio Catalano, Pier Francesco Fabbri, Adam C. S. Kazmi, Katherine L. Reinberger, et al. 2022. "The Diverse Genetic Origins of a Classical Period Greek Army." *Proceedings of the National Academy of Sciences of the United States of America* 119 (41): e2205272119.

Skourtanioti, Eirini, Yilmaz S. Erdal, Marcella Frangipane, Francesca Balossi Restelli, K. Aslıhan Yener, Frances Pinnock, Paolo Matthiae, et al. 2020. "Genomic History of Neolithic to Bronze Age Anatolia, Northern Levant, and Southern Caucasus." *Cell* 181 (5): 1158–75.e28.

Zalloua, Pierre A., Daniel E. Platt, Mirvat El Sibai, Jade Khalife, Nadine Makhoul, Marc Haber, Yali Xue, et al. 2008. "Identifying Genetic Traces of Historical Expansions: Phoenician Footprints in the Mediterranean." *American Journal of Human Genetics* 83 (5): 633–42.

Zalloua, Pierre, Catherine J. Collins, Anna Gosling, Simone Andrea Biagini, Benjamí Costa, Olga Kardailsky, Lorenzo Nigro, Wissam Khalil, Francesc Calafell, and Elizabeth Matisoo-Smith. 2018. "Ancient DNA of Phoenician Remains Indicates Discontinuity in

the Settlement History of Ibiza." *Scientific Reports* 8 (1): 17567.
Zaro, Valentina, Maria A. Spyrou, Stefania Vai, Guido A. Gnecchi-Ruscone, Alessandra Modi, Alexander Peltzer, Angela Mötsch, et al. 2021. "The Origin and Legacy of the Etruscans through a 2000-Year Archeogenomic Time Transect." *Science Advances*, September. https://doi.org/10.1126/sciadv.abi7673.
